# Supplementary material for: Accessing ladder-shape azetidine-fused indoline pentacycles through intermolecular regiodivergent aza-Paternò–Büchi reactions
Source: Nat Commun. 2024 Feb 16;15:1431. doi: 10.1038/s41467-024-45687-0 (PMC10873392; doi:10.1038/s41467-024-45687-0)
Supplement: Supplementary file 1 — Supplementary Information [file 41467_2024_45687_MOESM1_ESM.pdf]

Supplementary Information

Accessing ladder-shape azetidine-fused indoline pentacycles through intermolecular regiodivergent aza-Paternò-Büchi reactions

Jianjian Huang[#1], Tai-Ping Zhou[#1], Ningning Sun[1], Huaibin Yu[2], Xixiang Yu[2], Rong-Zhen Liao*, Weijun Yao[3], Zhifeng Dai[3,4], Guojiao Wu[1], Fangrui Zhong*[1]

1 Hubei Engineering Research Center for Biomaterials and Medical Protective Materials, Hubei Key Laboratory of Bioinorganic Chemistry & Materia Medica, School of Chemistry and Chemical Engineering, Huazhong University of Science and Technology (HUST), 1037 Luoyu Road, Wuhan 430074, China.

2 Zhengzhou Research Institute, Harbin Institute of Technology, Zhengzhou 450000, China.

3 School of Chemistry and Chemical Engineering, Zhejiang Sci-Tech University, Hangzhou 310018, China.

4 Longgang Institute of Zhejiang Sci-Tech University, Wenzhou, 325802, China

[#] These authors contributed equally to this work

e-Mail: rongzhen@hust.edu.cn or chemzfr@hust.edu.cn

Table of Contents

Supplementary Information 1

1. General information 3

2. Photocatalysts overview 4

3. Experimental Section 5

3.1 Reaction condition optimizations 5

3.2 General procedure for intermolecular aza-Paternò-Büchi reaction 9

3.3 Characterization data of azetidine products 9

3.4 Gram-scale reaction 35

3.5 Mechanistic studies 35

3.5.1 UV/Vis absorption spectra 35

3.5.2 Electrochemical measurements 36

3.5.3 Stern-Volmer quenching studies 37

3.5.4 Photocycloaddition via direct excitation 38

3.5.5 Triplet energy quenching experiments 38

4.Computational results 39

4.1 Calculation details 39

4.2 Optimized structures 43

4.3 Tables of energies 44

4.4 PMI analysis for 3D score 45

5. Supplementary references 46

6. NMR spectra 48

1. General information

Unless otherwise noted, all chemicals and reagents were obtained from commercial suppliers and used without further purification unless otherwise stated. Substrates were synthesized according to the literature procedures[1-9]. Reactions were monitored by TLC. ^{1}H NMR and ^{13}C NMR spectra were recorded on 400 MHz or 600 MHz Bruker spectrometers. Chemical shifts of ^{1}H NMR were reported in part per million relative to the $CDCl_3$ residual peak (δ 7.26) or DMSO-d_6 (δ 2.50). Chemical shifts of ^{13}C NMR were reported relative to $CDCl_3$ (δ 77.16) or DMSO-d_6 (δ 39.52). The abbreviations used are as follows: s (singlet), d (doublet), t (triplet), quart. (quartet), quint. (quintet), m (multiplet), br (broad). Multiplets that arise from accidental equality of coupling constants of magnetically non-equivalent protons are marked as virtual (*virt.*). High-resolution mass spectra (HRMS) data were measured on an ESI-micro TOF. Reactions were monitored by TLC analysis using silica gel 60 Å F-254 thin layer plates and compounds were visualized with a UV light at 254 nm or 365 nm. Further visualization was achieved by staining with or $KMnO_4$ followed by heating on a hot plate. Flash column chromatography was performed on silica gel 60 Å, 10-40 μm. The absorption spectrum of substrate and photosensitizer was detected on the instrument Varioskan LUX (Thermo). Photocatalysts are commercially available.

Photochemical setup: Visible light-mediated reactions were carried out using Photoreactions performed with WATTCAS photoreactor (WP-TEC-1020LC). The reactor temperature was monitored throughout the reaction with a temperature probe placed adjacent to the reaction vessel and maintained at 30ºC by cooling with low-temperature warehouse.

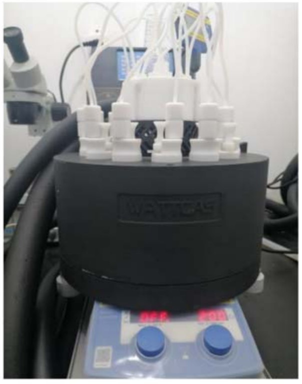

Supplementary Fig. 1. The reaction setup and emission spectrum of the lamp.

2. Photocatalysts overview

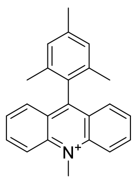

[MesAcr](ClO_4)
E_T = 44.7 kcal mol^{-1}
$E_{1/2}$ $^{[PC]^*/[PC]^-}$ = +0.57 V

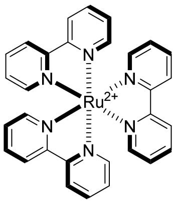

[Ru(bpy)$_3$](PF_6)$_2$
E_T = 46.5 kcal mol^{-1}
$E_1/_2$ $^{[PC]+/[PC]^*}$ = -0.81 V
$E_{1/2}$ $^{[PC]^*/[PC]^-}$ = +0.77 V

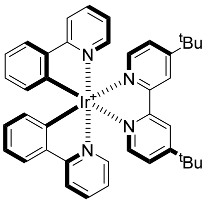

[Ir(ppy)$_2$(dtbbpy)](PF_6)

E_T = 49.2 kcal mol^{-1}
$E_1/_2$ $^{[PC]+/[PC]^*}$ = -0.96 V
$E_{1/2}$ $^{[PC]^*/[PC]^-}$ = +0.66 V

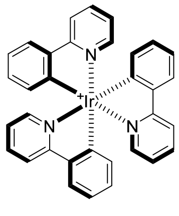

fac-[Ir(ppy)$_3$]
E_T = 58.1 kcal mol^{-1}
$E_1/_2$ $^{[PC]+/[PC]^*}$ = -1.73 V
$E_{1/2}$ $^{[PC]^*/[PC]^-}$ = +0.31 V

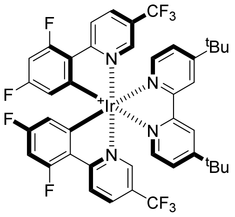

[Ir(dF(CF$_3$)ppy)$_2$(dtbbpy)](PF_6)
E_T = 61.8 kcal mol^{-1}
$E_1/_2$ $^{[PC]+/[PC]^*}$ = -0.89 V
$E_{1/2}$ $^{[PC]^*/[PC]^-}$ = +1.21 V

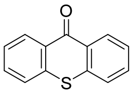

TXO(thioxanthone) E_T
= 65.5 kcal mol^{-1} $E_1/_2$
$^{[PC]+/[PC]^*}$ = -1.11 V
$E_{1/2}$ $^{[PC]^*/[PC]^-}$ = +1.18 V

Supplementary Fig. 2. Overview of photocatalysts used in this study. All potentials (given versus the saturated calomel electrode (SCE)) and triplet state energy (E_T) values were obtained from the literature[10-11].

3. Experimental Section

3.1 Reaction condition optimizations

Supplementary Table 1. Substrate evaluation for photochemical [2+2] cycloaddition

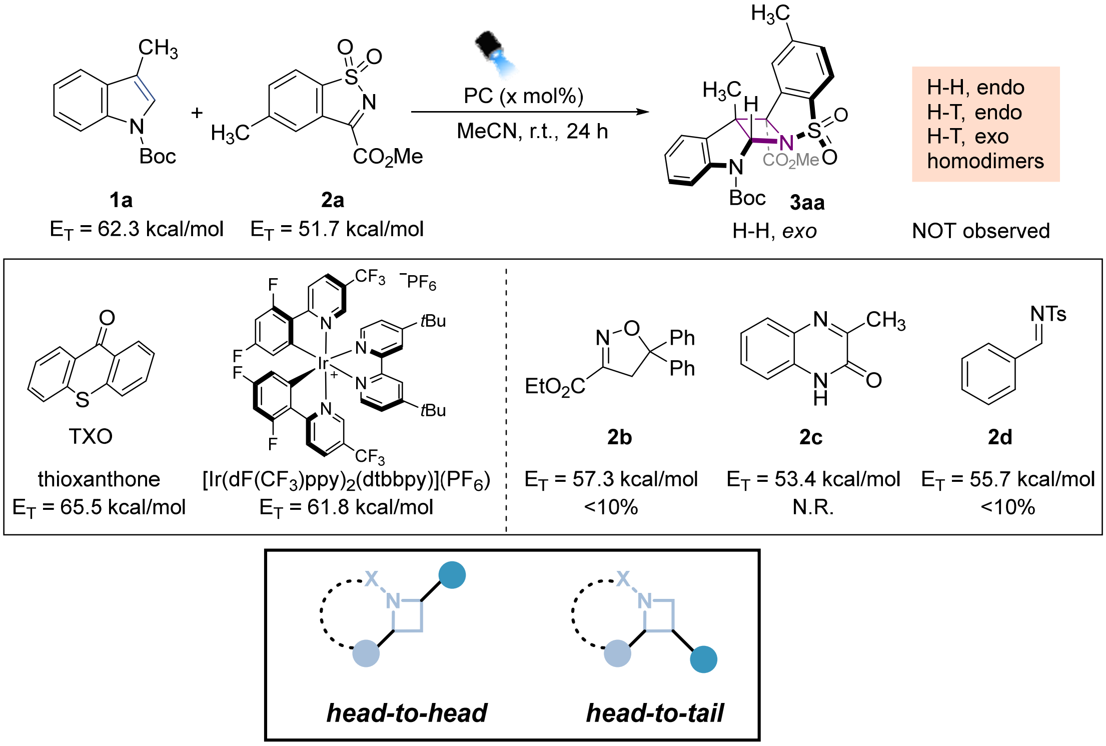

Entry[a]	2	PC (x mol%)	Yield (%)[b]
1	**2a**	TXO (5%)	75
2	**2a**	TXO (10%)	82[c]
3	**2a**	TXO (5%)	0[d]
4	**2b**	TXO (5%)	N. D.
5	**2c**	TXO (5%)	0
6	**2d**	TXO (5%)	N. D.
7	**2a**	$Ir(dF(CF_3)ppy]_2(dtbbpy)PF_6$(2%)	23
8	**2b**	$Ir(dF(CF_3)ppy]_2(dtbbpy)PF_6$(2%)	N. D.
9	**2c**	$Ir(dF(CF_3)ppy]_2(dtbbpy)PF_6$(2%)	N. D.
10	**2d**	$Ir(dF(CF_3)ppy]_2(dtbbpy)PF_6$(2%)	N. D.

[a]Reaction conditions: A solution of **1a** (0.2 mmol), **2a** (0.1 mmol), and photosensitizer in MeCN (2 mL) was irradiated by purple LEDs (λ_{max} = 405 nm, for entries 1-6) or blue LEDs (λ_{max} = 455 nm, for entries 7-10) at room temperature under nitrogen for 24 h. [b]Isolated yield of **3aa**. [c]Catalyst

loading (10 mol%) was used with 405 nm LEDs. [d]In dark. Ts = Tosyl; Boc = *tert*-butoxycarbonyl. N. D. = not determined.

Note: The nomenclature used in our paper to describe the regiochemistry of azetidines is based on Schindler review (Chem. Sci., 2020, 11, 7553): Regioisomers, for which the nitrogen is in a 1,2-relationship with the substituent of the highest priority originating from the alkene component, are referred to as head-to-head. In contrast, head-to-tail regioisomers feature a 1,3-relationship.

Supplementary Table 2. Examination of photocatalysts

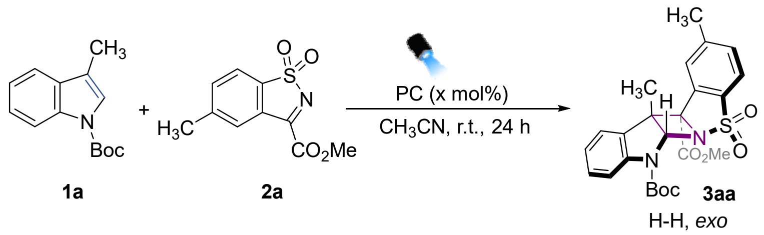

Entry[a]	PC (x mol%)	Yield (%)[b]
1	TXO (5%)	75
2	TXO (10%)	82
3	$Ir(dF(CF_3)ppy]_2(dtbbpy)PF_6$(2%)	23
4	$Ir(ppy)_3$ (2%)	8
5	$Ir(ppy)_2$ (dtbbpy)PF_6 (2%)	0
6	$Ru(bpy)_3Cl_2$ (2%)	0
7	[Mes-Acr](ClO_4) (10 %)	0
8	4CzIPN (5%)	0

[a]Reaction conditions: A solution of indole **1a** (0.2 mmol), imine **2a** (0.1 mmol), and photocatalyst (PC) in MeCN (2 mL) was irradiated by purple LEDs (λ_{max} = 405 nm, for entries 1-2) or blue LEDs (λ_{max} = 455 nm, for entries 3-8) at room temperature under nitrogen for 24 h. [b]Isolated yield of **3aa**. Boc = *tert*-butoxycarbonyl.

Supplementary Table 3. The effect of solvent

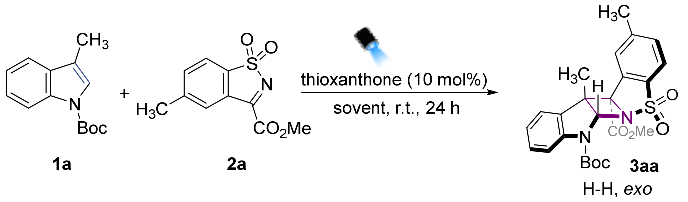

Entry[a]	Sovlent	Yield (%)[b]
1	acetone	63
2	EtOAc	75
3	CH_2Cl_2	72
4	DMF	68
5	$PhCF_3$	79
6	MeOH	41
7	HFIP	trace
**8**	**MeCN**	**82**

[a]Reaction conditions: A solution of indole **1a** (0.2 mmol), imine **2a** (0.1 mmol), and thioxanthone (10 mol%) in sovlent (2 mL) was irradiated by purple LEDs (λ_{max} = 405 nm) at room temperature under nitrogen for 24 h. [b]Isolated yield of **3aa**.

Supplementary Table 4. The effect of indole equivalents

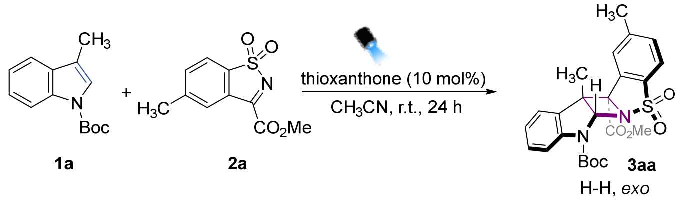

Entry[a]	Equivalents of **1a**	Yield (%)[b]
1	1	62
2	1.5	77
3	2	82
4	5	83

[a]Reaction conditions: A solution of indole **1a**, **2a** (0.1 mmol), and thioxanthone (10 mol%) in MeCN (2 mL) was irradiated by purple LEDs (λ_{max} = 405 nm) at room temperature under nitrogen for 24 h. [b]Isolated yield of **3aa**.

Results of other substrates tested in this study

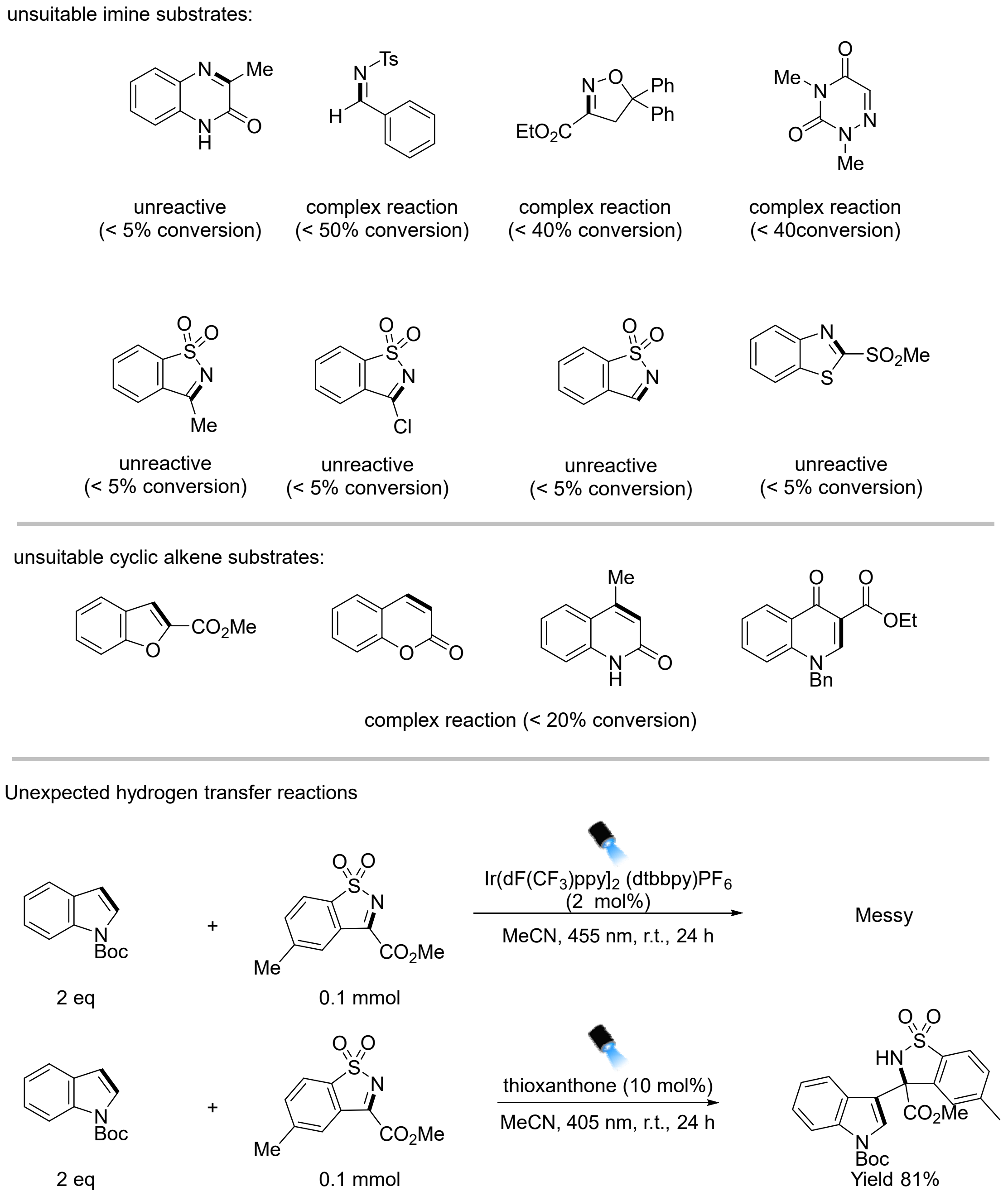

Supplementary Fig. 3. Additional substrates evaluated in the developed intermolecular aza Paternò-Büchi reaction.

3.2 General procedure for intermolecular aza-Paternò-Büchi reaction

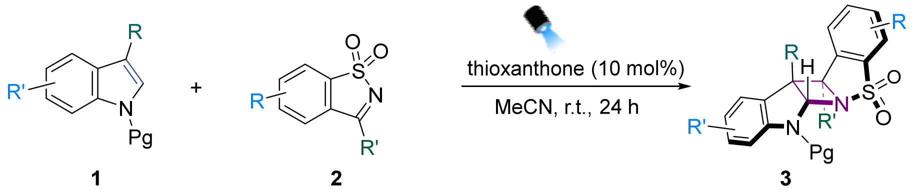

To a 10 mL glass reaction tube containing a stirring bar was added indole **1a** (0.2 mmol), imine **2a** (0.1 mmol) and thioxanthone (0.01 mmol) in MeCN (2 mL). The reaction mixture was deoxygenated by bubbling N_2 for 10 min, and was then illuminated under LEDs (λ_{max} = 405 nm, 10 W) at room temperature for 24 h. The crude mixture was directly purified through flash-column chromatography using silica gel and eluent containing hexane/ethyl acetate to obtain the desired products.

3.3 Characterization data of azetidine products

Product 3aa

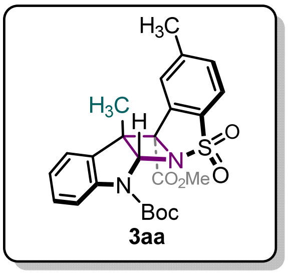

A white solid, 38.6 mg, 82% yield.

TLC: R_f = 0.55 (Hexane/EtOAc = 3:1) [UV].

^{1}H NMR (400 MHz, $CDCl_3$): δ 7.97 (br s, 1H), 7.74 (d, J = 8.1 Hz, 1H), 7.49 (d, J = 8.1 Hz, 1H), 7.38 – 7.32 (m, 1H), 7.30 – 7.26 (m, 1H), 7.10 – 7.05 (m, 1H), 5.89 (s, 1H), 3.47 (s, 3H), 2.56 (s, 3H), 1.62 (s, 9H), 1.40 (s, 3H).

^{13}C NMR (101 MHz, $CDCl_3$): δ 168.2, 150.9, 144.7, 143.2, 135.1, 134.8, 132.3, 130.8, 130.1, 127.5, 124.7, 123.1, 122.2, 116.2, 82.9, 82.7, 80.4, 54.1, 52.9, 28.3, 21.9, 20.3.

HRMS (ESI): Calcd for $C_{24}H_{27}N_2O_6S$ $[M+H]^+$: 471.1584; Found 471.1584.

Product 3ba

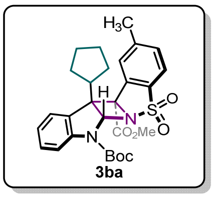

A white solid, 45.6 mg, 87% yield.

TLC: R_f = 0.57 (Hexane/EtOAc = 3:1) [UV].

^{1}H NMR (400 MHz, $CDCl_3$): δ 7.98 (br s, 1H), 7.84 (s, 1H), 7.73 (d, *J* = 8.1 Hz, 1H), 7.49 (d, *J* = 8.1Hz, 1H), 7.40 – 7.31 (m, 2H), 7.09 – 7.01 (m, 1H), 5.90 (s, 1H), 3.45 (s, 3H), 2.55 (s, 3H), 2.33 – 2.20 (m, 1H), 1.77 – 1.69 (m, 2H), 1.62 (s, 9H), 1.51 – 1.25 (m, 5H), 0.84 – 0.68 (m, 1H).

^{13}C NMR (101 MHz, $CDCl_3$): δ 168.6, 150.9, 144.7, 135.3, 135.1, 132.2, 130.1, 127.4, 126.4, 122.8, 122.1, 116.3, 82.9, 81.3, 81.1, 60.9, 52.9, 41.9, 28.4, 27.9, 27.5, 25.7, 25.5, 21.9.

HRMS (ESI): Calcd for $C_{28}H_{33}N_2O_6S$ $[M+H]^+$: 525.2054; Found 525.2047.

Product 3ca

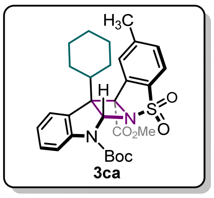

A white solid, 45.2 mg, 84% yield.

TLC: R_f = 0.58 (Hexane/EtOAc = 3:1) [UV].

^{1}H NMR (400 MHz, $CDCl_3$): δ 7.99 (s, 1H), 7.80 (s, 1H), 7.76 (d, *J* = 8.1 Hz, 1H), 7.53 – 7.49 (m, 1H), 7.40 –7.33 (m, 2H), 7.10 – 7.02 (m, 1H), 5.89 (s, 1H), 3.50 (s, 3H), 2.55 (s, 3H), 2.47 (d, *J* = 12.1 Hz, 1H), 1.91 – 1.81 (m, 1H), 1.70 – 164 (m, 1H), 1.61 (s, 9H), 1.57 – 1.42 (m, 3H), 1.32 – 1.24 (m, 1H), 1.11 – 0.98 (m, 3H), 0.57 – 0.44 (m, 1H).

^{13}C NMR (101 MHz, $CDCl_3$): δ 168.6, 150.7, 144.8, 144.5, 135.2, 132.3, 130.0, 127.5, 127.3, 122.5, 122.4, 116.8, 82.8, 81.6, 80.9, 60.9, 53.0, 41.9, 28.3, 27.9, 25.8, 21.9.

HRMS (ESI): Calcd for $C_{29}H_{35}N_2O_6S$ $[M+H]^+$: 539.2210; Found 539.2210.

Product 3da

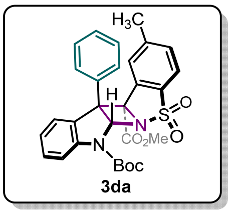

A white solid, 48.8 mg, 88% yield.

TLC: R_f = 0.62 (Hexane/EtOAc = 3:1) [UV].

^{1}H NMR (400 MHz, $CDCl_3$): δ 8.02 (br s, 1H), 7.62 – 7.60 (t, *J* = 8.1 Hz, 2H), 7.41 (d, *J* = 8.1 Hz, 2H), 7.38 – 7.24 (m, 6H), 7.14 – 7.07 (m, 1H), 6.64 (s, 1H), 3.59 (s, 3H), 2.25 (s, 3H), 1.66 (s, 9H).

^{13}C NMR (101 MHz, $CDCl_3$): δ 168.5, 150.7, 144.1, 142.9, 135.1, 134.8, 134.6, 131.9, 130.2, 128.7, 128.6, 128.3, 126.8, 126.5, 123.2, 121.6, 116.9, 83.2, 81.0, 80.9, 61.4, 53.2, 28.3, 21.5.

HRMS (ESI): Calcd for $C_{29}H_{28}N_2NaO_6S$ $[M+Na]^+$: 555.1560; Found 555.1562.

Product 3ea

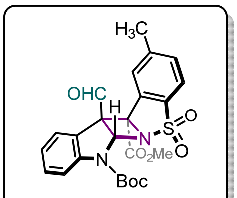

3ea

A white solid, 44.6 mg, 92% yield.

TLC: R_f = 0.48 (Hexane/EtOAc = 3:1) [UV].

^{1}H NMR (400 MHz, $CDCl_3$): δ 9.79 (s, 1H), 8.02 (br s, 1H), 7.73 (d, J = 8.1 Hz, 1H), 7.63 (s, 1H), 7.54 – 7.40 (m, 3H), 7.18 – 7.11 (m, 1H), 6.51 (s, 1H), 3.58 (s, 3H), 2.52 (s, 3H), 1.62 (s, 9H).

^{13}C NMR (101 MHz, $CDCl_3$): δ 190.9, 167.0, 150.5, 145.4, 144.6, 134.6, 133.0, 131.6, 127.6, 126.2, 123.4, 122.4, 116.9, 83.6, 79.7, 77.6, 64.4, 53.5, 28.3, 21.9.

HRMS (ESI): Calcd for $C_{24}H_{25}N_2O_7S$ [M+H]+: 485.1377; Found 485.1382.

Product 3fa

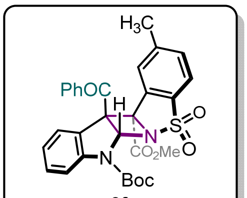

3fa

A white solid, 49.9 mg, 89% yield.

TLC: R_f = 0.51 (Hexane/EtOAc = 3:1) [UV].

^{1}H NMR (400 MHz, $CDCl_3$): δ 8.14 (s, 1H), 8.08 (br s, 1H), 7.61 (d, J = 8.1 Hz, 1H), 7.52 – 7.29 (m, 8H), 7.12 – 7.05 (m, 1H), 6.61 (s, 1H), 3.56 (s, 3H), 2.56 (s, 3H), 1.63 (s, 9H).

^{13}C NMR (101 MHz, $CDCl_3$): δ 192.9, 166.8, 150.7, 144.7, 143.1, 134.7, 134.1, 133.9, 133.8, 132.5, 131.5, 130.8, 128.9, 127.5, 126.1, 123.7, 121.3, 117.5, 83.6, 81.5, 80.9, 66.3, 53.3, 28.3, 21.9.

HRMS (ESI): Calcd for $C_{30}H_{29}N_2O_7S$ $[M+H]^+$: 561.1690; Found 561.1702.

Product 3ga

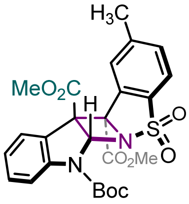

3ga

A white solid, 47.9 mg, 93% yield.

TLC: R_f = 0.51 (Hexane/EtOAc = 3:1) [UV].

^{1}H NMR (400 MHz, $CDCl_3$): δ 7.98 (br s, 1H), 7.72 (d, J = 8.1 Hz, 2H), 7.67 (d, J = 8.1 Hz, 1H), 7.49 (d, J = 8.1 Hz, 1H), 7.41 (t, J = 8.1 Hz, 1H), 7.11 (t, J = 8.1 Hz, 1H), 6.50 (s, 1H), 3.61 (s, 3H), 3.53 (s, 3H), 2.53 (s, 3H), 1.63 (s, 9H).

^{13}C NMR (101 MHz, $CDCl_3$): δ 166.5, 165.6, 150.6, 144.9, 144.1, 134.7, 133.9, 132.7, 131.3, 127.1, 126.9, 123.5, 123.2, 121.9, 116.4, 83.4, 79.6, 79.2, 61.4, 53.3, 52.9, 28.3, 21.8.

HRMS (ESI): Calcd for $C_{25}H_{27}N_2O_8S$ $[M+H]^+$: 515.1483; Found 515.1483.

Product 3ha

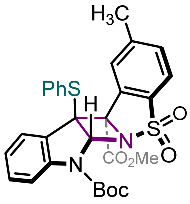

3ha

A white solid, 31.7 mg, 54% yield.

TLC: R_f = 0.55 (Hexane/EtOAc = 3:1) [UV].

^{1}H NMR (400 MHz, $CDCl_3$): δ 7.95 (s, 1H), 7.77 (d, J = 8.1 Hz, 1H), 7.71 (br s, 1H), 7.55 (d, J = 8.1 Hz, 1H), 7.43 (d, J = 8.1 Hz, 1H), 7.27 – 7.21 (m, 1H), 7.20 – 7.13 (m, 1H), 7.09 – 6.95 (m, 5H), 6.18 (s, 1H), 3.53 (s, 3H), 2.62 (s, 3H), 1.56 (s, 9H).

^{13}C NMR (101 MHz, $CDCl_3$): δ 167.2, 150.3, 144.6, 143.9, 135.4, 134.5, 133.9, 132.7, 130.5, 129.5, 128.8, 128.2, 127.9, 127.6, 126.5, 123.2, 122.1, 116.4, 84.3, 82.9, 80.7, 61.7, 53.2, 28.2, 22.0.

HRMS (ESI): Calcd for $C_{29}H_{28}N_2NaO_6S_2$ $[M+Na]^+$: 587.1281; Found 587.1286.

Product 3ia

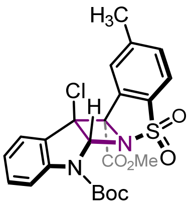

A white solid, 42.7 mg, 87% yield.

TLC: R_f = 0.58 (Hexane/EtOAc = 3:1) [UV].

^{1}H NMR (400 MHz, $CDCl_3$): δ 8.00 (br s, 1H), 7.86 (s, 1H), 7.74 (d, J = 8.1 Hz, 1H), 7.56 – 7.50 (m, 2H), 7.48 – 7.40 (m, 1H), 7.20 – 7.12 (m, 1H), 6.18 (s, 1H), 3.57 (s, 3H), 2.58 (s, 3H), 1.62 (s, 9H).

^{13}C NMR (101 MHz, $CDCl_3$): δ 166.7, 150.2, 144.8, 143.2, 134.1, 133.8, 132.9, 131.9, 128.9, 127.3, 126.2, 123.8, 121.8, 116.7, 84.5, 83.7, 81.4, 67.6, 53.4, 28.2, 21.9.

HRMS (ESI): Calcd for $C_{23}H_{24}ClN_2O_6S$ $[M+H]^+$: 491.1038; Found 491.1039.

Product 3ja

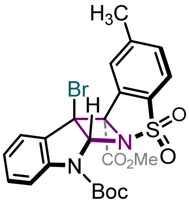

A white solid, 45.6 mg, 82% yield.

TLC: R_f = 0.62 (Hexane/EtOAc = 3:1) [UV].

^{1}H NMR (400 MHz, $CDCl_3$): δ 7.98 (s, 1H), 7.88 (s, 2H), 7.73 (d, J = 8.1 Hz, 1H), 7.57 – 7.51 (m, 2H), 7.44 – 7.37 (m, 1H), 7.21 – 7.12 (m, 1H),, 6.26 (s, 1H), 3.57 (s, 3H), 2.59 (s, 3H), 1.62 (s, 9H).

^{13}C NMR (101 MHz, $CDCl_3$): δ 166.6, 150.2, 144.6, 142.7, 135.0, 133.9, 132.9, 131.8, 128.9, 128.7, 126.9, 123.8, 121.7, 116.6, 84.6, 83.7, 81.3, 57.4, 53.4, 28.2, 22.0.

HRMS (ESI): Calcd for $C_{23}H_{23}BrN_2NaO_6S$ $[M+Na]^+$: 557.0352; Found 557.0359.

Product 3ka

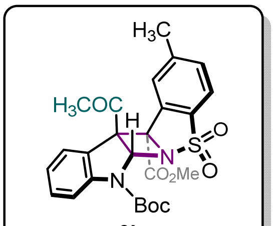

3ka

A white solid, 47.4 mg, 91% yield.

TLC: R_f = 0.53 (Hexane/EtOAc = 3:1) [UV].

^{1}H NMR (400 MHz, $CDCl_3$): δ 8.01 (br s, 1H), 7.77 (s, 1H), 7.70 (d, *J* = 8.1 Hz, 1H), 7.52 (d, *J* = 8.1 Hz, 1H), 7.47 (d, *J* = 8.1 Hz, 1H), 7.45 – 7.39 (m, 1H), 7.11 (t, *J* = 8.2 Hz, 1H), 6.51 (s, 1H), 3.55 (s, 3H), 2.52 (s, 3H), 2.23 (s, 3H), 1.63 (s, 9H).

^{13}C NMR (101 MHz, $CDCl_3$): δ 198.6, 167.2, 150.6, 145.0, 144.3, 134.4, 133.6, 132.8, 131.4, 128.2, 126.3, 124.1, 123.3, 121.9, 116.9, 83.6, 79.9, 79.2 66.5, 53.3, 28.3, 28.1, 21.9.

HRMS (ESI): Calcd for $C_{25}H_{26}N_2NaO_7S$ $[M+H]^+$: 521.1353; Found 521.1368.

Product 3la

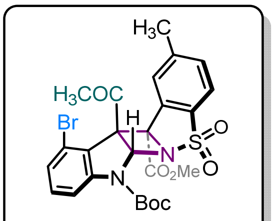

3la

A white solid, 45.1 mg, 78% yield.

TLC: R_f = 0.51 (Hexane/EtOAc = 3:1) [UV].

^{1}H NMR (400 MHz, $CDCl_3$): δ 8.10 – 7.98 (m, 2H), 7.65 (d, *J* = 8.1 Hz, 1H), 7.48 – 7.42(m, 1H), 7.32 (t, *J* = 8.1 Hz, 1H), 7.28 – 7.24 (m, 1H), 6.24 (s, 1H), 3.48 (s, 3H), 2.57 (s, 3H), 1.93 (s, 3H), 1.66 (s, 9H).

^{13}C NMR (101 MHz, $CDCl_3$): δ 198.6, 167.2, 150.6, 145.0, 144.3, 134.4, 133.6, 132.8, 131.4, 128.2, 126.3, 124.1, 123.3, 121.9, 116.9, 83.6, 79.9, 79.2, 66.5, 53.3, 28.3, 28.1, 21.9.

HRMS (ESI): Calcd for $C_{25}H_{26}BrN_2O_7S$ $[M+H]^+$: 577.0639; Found 577.0646.

Product 3ma

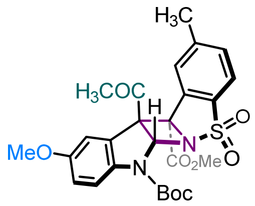

3ma

A white solid, 29.2 mg, 55% yield.

TLC: R_f = 0.55 (Hexane/EtOAc = 3:1) [UV].

^{1}H NMR (400 MHz, $CDCl_3$): δ 7.92 (br s, 1H), 7.79 (s, 51H), 7.71 (d, J = 8.1 Hz, 55H), 7.47 (d, J = 8.1 Hz, 1H), 7.08 (d, J = 2.6 Hz, 1H), 6.97 (dd, J = 8.9, 2.6 Hz, 1H), 6.49 (s, 1H), 3.83 (s, 3H), 3.60 (s, 3H), 2.53 (s, 3H), 2.19 (s, 3H), 1.63 (s, 9H).

^{13}C NMR (101 MHz, $CDCl_3$): δ 13C NMR (101 MHz, CDCl3) δ 198.58, 167.06, 156.01, 150.62, 144.93, 137.81, 134.50, 133.68, 132.74, 128.46, 125.38, 121.86, 117.52, 116.51, 112.19, 83.29, 79.78, 79.72, 66.42, 55.90, 53.35, 28.32, 28.05, 21.86.

HRMS (ESI): Calcd for $C_{26}H_{29}N_2O_8S$ $[M+H]^+$: 529.1639; Found 529.1641.

Product 3na

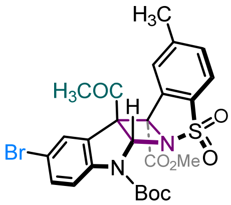

3na

A white solid, 42.6 mg, 74% yield.

TLC: R_f = 0.52 (Hexane/EtOAc = 3:1) [UV].

^{1}H NMR (400 MHz, $CDCl_3$): δ 7.92 (s, 1H), 7.72 (d, *J* = 8.7 Hz, 2H), 7.66 (d, *J* = 2.1 Hz, 1H), 7.54 (dd, *J* = 8.7, 2.1 Hz, 1H), 7.49 (d, *J* = 8.7 Hz, 1H), 6.48 (s, 1H), 3.64 (s, 3H), 2.53 (s, 3H), 2.22 (s, 3H), 1.63 (s, 9H).

^{13}C NMR (101 MHz, $CDCl_3$): δ 198.1, 166.8, 150.3, 145.2, 143.5, 134.4, 134.3, 133.2, 132.9, 129.3, 128.1, 126.2, 122.0, 118.2, 115.6, 84.1, 79.8, 79.4, 65.9, 53.5, 28.3, 28.3, 21.9.

HRMS (ESI): Calcd for $C_{25}H_{26}BrN_2O_7S$ $[M+H]^+$: 577.0639; Found 577.0642.

Product 3oa

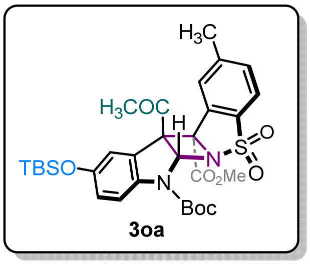

A white solid, 32.1 mg, 51% yield.

TLC: R_f = 0.61 (Hexane/EtOAc = 3:1) [UV].

^{1}H NMR (400 MHz, $CDCl_3$): δ 7.88 (br s, 1H), 7.77 (s, 1H), 7.71 (d, *J* = 7.9 Hz, 1H), 7.47 (d, *J* = 7.9 Hz, 1H), 7.04 (d, *J* = 2.5 Hz, 1H), 6.90 (dd, *J* = 7.9, 2.5 Hz, 1H), 6.48 (s, 1H), 3.61 (s, 3H), 2.52 (s, 3H), 2.22 (s, 3H), 1.63 (s, 9H), 1.02 (s, 9H), 0.24 (s, 3H), 0.22 (s, 3H).

^{13}C NMR (101 MHz, $CDCl_3$): δ 198.5, 167.2, 151.7, 150.6, 145.0, 134.4, 133.8, 132.8, 128.2, 122.8, 121.9, 118.3, 117.4, 83.3, 79.7, 79.5, 53.4, 28.3, 30.0, 25.7, 21.9, 18.2, -4.4, -4.5.

HRMS (ESI): Calcd for $C_{31}H_{41}N_2O_8SSi$ $[M+H]^+$: 629.2347 ; Found 629.2360.

Product 3pa

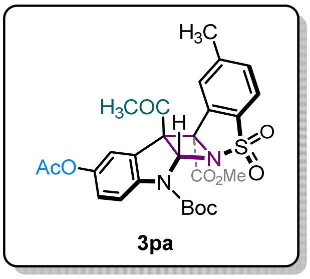

A white solid, 43.4 mg, 78% yield.

TLC: R_f = 0.46 (Hexane/EtOAc = 3:1) [UV].

^{1}H NMR (400 MHz, $CDCl_3$): δ 8.02 (br s, 1H), 7.72 (d, *J* = 8.1 Hz, 2H), 7.48 (d, *J* = 8.1 Hz, 1H), 7.31 (d, *J* = 2.4 Hz, 1H), 7.12 (dd, *J* = 8.1, 2.4 Hz, 1H), 6.51 (s, 1H), 3.63 (s, 3H), 2.52 (s, 3H), 2.32 (s, 3H), 1.63 (s, 9H).

^{13}C NMR (101 MHz, $CDCl_3$): δ 198.3, 169.4, 167.2, 150.5, 146.3, 145.2, 141.9, 134.34 133.5, 132.9, 127.9, 124.4, 121.9, 120.3, 117.2, 83.8, 79.7, 79.5, 66.2, 53.7, 28.3, 21.9, 21.0.

HRMS (ESI): Calcd for $C_{27}H_{29}N_2O_9S$ $[M+H]^+$: 557.1588; Found 557.1584.

Product 3qa

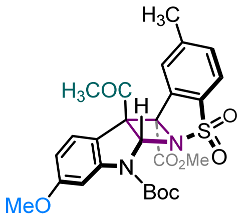

3qa

A white solid, 39.6 mg, 72% yield.

TLC: R_f = 0.57 (Hexane/EtOAc = 3:1) [UV].

^{1}H NMR (400 MHz, $CDCl_3$): δ 7.75 (s, 1H), 7.70 (d, J = 7.9 Hz, 1H), 7.66 (br s, 1H), 7.46 (d, J = 7.9 Hz, 1H), 7.39 (d, J = 7.9 Hz, 1H), 6.65 (dd, J = 8.6, 2.4 Hz, 1H), 6.49 (s, 1H), 3.86 (s, 3H), 3.60 (s, 3H), 2.52 (s, 3H), 2.24 (s, 3H), 1.64 (s, 9H).

^{13}C NMR (101 MHz, $CDCl_3$): δ 198.7, 167.4, 162.5, 150.6, 145.9, 145.0, 134.5, 133.7, 132.7, 128.1, 126.8, 121.9, 115.6, 110.3, 102.1, 83.6, 80.0, 79.8, 66.0, 55.7, 53.4, 28.3, 27.9, 21.9.

HRMS (ESI): Calcd for $C_{26}H_{28}N_2NaO_8S$ $[M+H]^+$: 551.1459; Found 551.1468.

Product 3ra

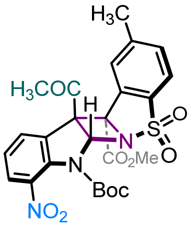

3ra

A white solid, 46.2 mg, 85% yield.

TLC: R_f = 0.49 (Hexane/EtOAc = 3:1) [UV].

^{1}H NMR (400 MHz, $CDCl_3$): δ 8.0 – 7.5 (m, 1H), 7.89 – 7.84 (m, 1H), 7.74 (d, J = 8.1 Hz, 1H), 7.69 (s, 1H), 7.52 (d, J = 8.1 Hz, 1H), 7.33 (t, J = 8.1 Hz, 1H), 6.60 (s, 1H), 3.56 (s, 3H), 2.53 (s, 3H), 2.21 (s, 3H), 1.55 (s, 9H).

^{13}C NMR (101 MHz, $CDCl_3$): δ 197.7, 166.8, 150.4, 145.5, 140.6, 137.2, 134.5, 133.2, 133.2, 130.9, 129.9, 127.9, 126.8, 124.6, 122.3, 85.3, 80.9, 79.3, 66.6, 53.5, 28.2, 27.9, 21.9..

HRMS (ESI): Calcd for $C_{25}H_{26}N_3O_9S$ $[M+H]^+$: 544.1384; Found 544.1389.

Product 3sa

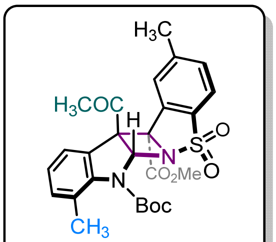

3sa

A white solid, 45.1 mg, 88% yield.

TLC: R_f = 0.48 (Hexane/EtOAc = 3:1) [UV].

^{1}H NMR (400 MHz, $CDCl_3$): δ 7.79 (s, 1H), 7.70 (d, *J* = 7.9 Hz, 1H), 7.47 (d, *J* = 7.9 Hz, 1H), 7.36 (d, *J* = 7.9 Hz, 1H), 7.29 (d, *J* = 7.9 Hz, 1H), 7.15 (t, *J* = 7.9 Hz, 1H), 6.57 (s, 1H), 3.53 (s, 3H), 2.51 (s, 3H), 2.39 (s, 3H), 2.22 (s, 3H), 1.55 (s, 9H)

^{13}C NMR (101 MHz, $CDCl_3$): δ 198.6, 167.0, 151.7, 145.0, 142.8, 134.6, 133.9, 133.9, 132.8, 130.6, 128.5, 127.6, 125.2, 123.2, 122.0, 83.0, 81.2, 79.5, 67.6, 53.2, 28.1, 27.5, 21.9, 20.5.

HRMS (ESI): Calcd for $C_{26}H_{29}N_2O_7S$ $[M+H]^+$: 513.1690 ; Found 513.1691.

Product 3ta

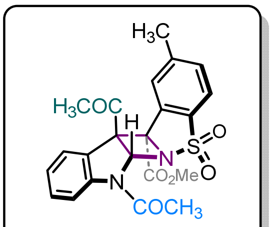

3ta

A white solid, 40.5 mg, 92% yield.

TLC: R_f = 0.38 (Hexane/EtOAc = 1:1) [UV].

^{1}H NMR (400 MHz, DMSO-*d6*): δ 8.18 (d, *J* = 8.1 Hz, 1H), 7.99 (d, *J* = 8.1 Hz, 1H), 7.80 (s, 1H), 7.67 – 7.63 (m, 1H), 7.56 – 7.48 (m, 2H), 7.30 – 7.23 (m, 1H), 6.68 (s, 1H), 3.58 (s, 3H), 2.50 (s, 3H), 2.44 (s, 3H), 2.15 (s, 3H).

^{13}C NMR (101 MHz, DMSO-*d6*): δ 199.6, 169.2, 166.3, 145.7, 144.4, 133.9, 133.7, 131.6, 128.8, 126.6, 125.1, 122.5, 118.4, 83.4, 80.1, 70.5, 53.8, 28.6, 24.2, 21.8.

HRMS (ESI): Calcd for $C_{22}H_{21}N_2O_6S$ $[M+H]^+$: 441.1115; Found 441.1110.

Product 3ua

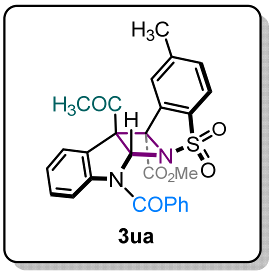

A white solid, 47.7 mg, 95% yield.

TLC: R_f = 0.40 (Hexane/EtOAc = 1:1) [UV].

^{1}H NMR (400 MHz, DMSO-*d6*): δ 7.85 (br s, 1H), 7.80 (d, *J* = 8.0 Hz, 1H), 7.71 – 7.65 (m, 2H), 7.60 – 7.55 (m, 4H), 7.55 – 7.47 (m, 3H), 7.34 – 7.28 (m, 1H), 6.50 (s, 1H), 3.57 (s, 3H), 2.47 (s, 3H), 2.28 (s, 3H).

^{13}C NMR (101 MHz, DMSO-*d6*): δ 199.4, 169.3, 166.3, 145.7, 144.5, 135.3, 134.1, 133.6, 133.2, 131.5, 131.3, 129.3, 127.9, 127.9, 127.1, 126.3, 125.3, 122.4, 118.1, 80.5, 80.1, 66.4, 53.8, 29.0, 21.8.

HRMS (ESI): Calcd for $C_{27}H_{23}N_2O_6S$ $[M+H]^+$: 503.1271; Found 503.1271.

Product 3va

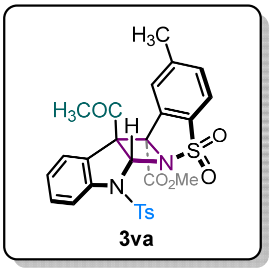

A white solid, 49.7 mg, 90% yield.

TLC: R_f = 0.35 (Hexane/EtOAc = 3:1) [UV].

^{1}H NMR (400 MHz, $CDCl_3$): δ 7.90 – 7.87 (m, 2H), 7.72 (d, *J* = 8.0 Hz, 1H), 7.69 (s, 1H), 7.62 (d, *J* = 8.0 Hz, 1H), 7.54 – 7.44 (m, 3H), 7.30 – 7.26 (m, 2H), 7.22 – 7.14 (m, 1H), 6.58 (s, 1H), 3.51 (s, 3H), 2.51 (s, 3H), 2.38 (s, 3H), 2.04 (s, 3H).

^{13}C NMR (101 MHz, $CDCl_3$): δ 198.2, 166.7, 145.3, 144.9, 143.4, 135.6, 134.3, 133.6, 132.9, 131.6, 129.9, 128.3, 127.6, 126.8, 125.8, 124.7, 122.1, 116.8, 81.2, 79.6, 67.3, 53.3, 27.9, 21.9, 21.6.

HRMS (ESI): Calcd for $C_{27}H_{25}N_2O_7S_2$ $[M+H]^+$: 553.1098 ; Found 553.1100.

Product 3wa

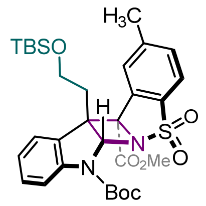

3wa

A white solid, 27.6 mg, 45% yield.

TLC: R_f = 0.56 (Hexane/EtOAc = 5:1) [UV].

^{1}H NMR (400 MHz, $CDCl_3$): δ 8.09 – 7.96 (m, 1H), 7.76 (d, *J* = 8.1 Hz, 1H), 7.71 (s, 1H), 7.49 (d, *J* = 8.1 Hz, 1H), 7.35 (t, *J* = 8.1 Hz, 1H), 7.18 (d, *J* = 8.1 Hz, 1H), 7.06 (t, *J* = 7.9 Hz, 1H), 6.14 (s, 1H), 3.54 – 3.46(m, 1H), 3.44 (s, 3H), 3.27 – 3.17 (m, 1H), 2.56 (s, 3H), 2.17 – 2.06 (m, 1H), 1.90 – 1.78 (m, 1H), 1.67 – 1.61 (m, 9H), 0.77 – 0.69 (m, 9H), -0.19 – -0.22 (m, 3H), -0.23 (d, *J* = 1.6 Hz, 3H).

^{13}C NMR (101 MHz, $CDCl_3$): δ 168.3, 150.9, 144.5, 135.0, 134.8, 132.3, 130.1, 127.6, 125.1, 122.5, 122.2, 116.2, 82.9, 82.7, 81.0, 59.1, 52.9, 35.6, 28.4, 25.9, 21.9, 18.2, -5.7, -6.0.

HRMS (ESI): Calcd for $C_{31}H_{43}N_2O_7SSi$ $[M+H]^+$: 615.2555; Found 615.2565.

Product 3xa

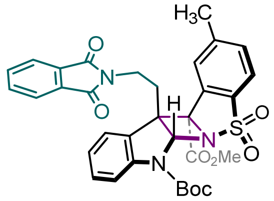

3xa

A white solid, 47.8 mg, 76% yield.

TLC: R_f = 0.51 (Hexane/EtOAc = 3:1) [UV].

^{1}H NMR (400 MHz, $CDCl_3$): δ 7.94 – 7.80 (m, 1H), 7.75 (d, *J* = 8.1 Hz, 1H), 7.73 – 7.64 (m, 5H), 7.49 (d, *J* = 8.1 Hz, 1H), 7.31 – 7.25 (m, 1H), 7.15 (t, *J* = 8.1 Hz, 1H), 6.93 (t, *J* = 7.5 Hz, 1H), 6.11 (s, 1H), 3,51 – 3.44 (m, 1H), 3.45 (s, 3H), 3,25 – 3.22 (m, 1H), 2.55 (s, 3H), 1.90 – 1.81 (m, 1H), 1.64 (s, 9H).

^{13}C NMR (101 MHz, $CDCl_3$): δ 167.9, 167.5, 150.6, 145.0, 134.8, 134.3, 133.9, 132.6, 131.7, 130.1, 127.5, 125.1, 123.2, 123.0, 122.4, 116.5, 83.1, 80.9, 80.8, 56.1, 53.0, 32.9, 30.7, 28.3, 22.0.

HRMS (ESI): Calcd for $C_{33}H_{32}N_3O_8S$ $[M+H]^+$: 630.1905; Found 630.1913.

Product 3ya

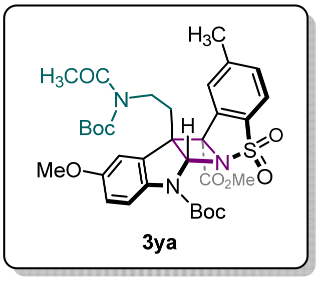

A white solid, 43.6 mg, 65% yield.

TLC: R_f = 0.61 (Hexane/EtOAc = 3:1) [UV].

^{1}H NMR (400 MHz, $CDCl_3$): δ 7.85 (d, J = 8.1 Hz, 1H), 7.72 (d, J = 8.1 Hz, 1H), 7.66 (s, 1H), 7.48 (d, J = 8.1, 1H), 6.92 – 6.83 (m, 2H), 6.00 (s, 1H), 3.81 (s, 3H), 3.49 (s, 3H), 3.42 – 3.29 (m, 1H), 3.19 – 3.07 (m, 1H), 2.53 (s, 3H), 2.39 –2.26 (m, 4H), 1.59 (s, 9H),1.57 – 1.47 (m, 1H), 1.37 (s, 9H).

^{13}C NMR (101 MHz, $CDCl_3$): δ 172.7, 167.7, 156.0, 152.4, 150.7, 144.9, 137.4, 134.8, 134.4, 132.5, 128.7, 127.5, 124.5, 122.2, 116.9, 115.7, 110.9, 83.5, 82.8, 81.5, 80.5, 56.1, 55.8, 53.1, 39.2, 31.5, 28.3, 27.9, 26.7, 21.9.

HRMS (ESI): Calcd for $C_{33}H_{42}N_3O_{10}S$ $[M+H]^+$: 672.2585; Found 672.2584.

Product 3kb

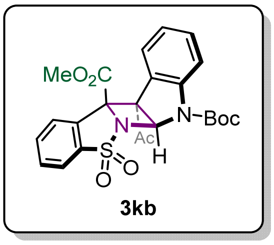

A white solid, 42.6 mg, 88% yield.

TLC: R_f = 0.53 (Hexane/EtOAc = 3:1) [UV].

^{1}H NMR (400 MHz, $CDCl_3$): δ 8.09 – 7.93 (m, 2H), 7.82 (d, J = 6.5 Hz, 1H), 7.76 – 7.63 (m, 2H), 7.51 (d, J = 6.5 Hz, 1H), 7.45 – 7.38 (m, 1H), 7.17 – 7.07 (m, 1H), 6.50 (s, 1H), 3.54 (s, 3H), 2.22 (s, 3H), 1.62 (s, 9H).

^{13}C NMR (101 MHz, $CDCl_3$): δ 198.5, 166.9, 150.5, 144.3, 137.0, 133.8, 133.2, 131.8, 131.5, 128.3, 126.2, 124.1, 123.4, 122.2, 116.9, 83.6, 80.1, 79.3, 66.5, 53.4, 28.3, 28.1..

HRMS (ESI): Calcd for $C_{24}H_{25}N_2O_7S$ $[M+H]^+$: 485.1377 Found 485.1384.

Product 3kc

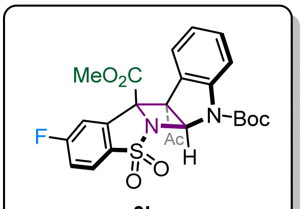

A yellow solid, 37.8 mg, 72% yield.

TLC: R_f = 0.61 (Hexane/EtOAc = 3:1) [UV].

^{1}H NMR (400 MHz, $CDCl_3$): δ 8.03 (br s, 1H), 7.81 (dd, J = 8.6, 2.3 Hz, 1H), 7.74 (dd, J = 8.6, 2.3 Hz, 1H), 7.48 – 7.41 (m, 2H), 7.37 (td, J = 8.6, 2.3 Hz, 1H), 7.12 (td, J = 8.6, 1.1 Hz, 1H), 6.51 (s, 1H), 3.56 (s, 3H), 2.20 (s, 3H), 1.63 (s, 9H).

^{13}C NMR (101 MHz, $CDCl_3$): δ 198.5, 166.7 (d, J = 19.4 Hz), 164.2, 150.5, 144.3, 136.6 (d, J = 9.9 Hz), 133.1 (d, J = 2.6 Hz), 131.7, 126.0, 124.2 (d, J = 9.9 Hz), 123.9, 123.49, 119.9 (d, J = 24.6 Hz), 117.00, 115.9 (d, J = 25.3 Hz), 83.8, 79.5, 79.5 (d, J = 2.2 Hz), 66.5, 53.5, 28.3, 27.9.

HRMS (ESI): Calcd for $C_{24}H_{23}FN_2NaO_7S$ $[M+Na]^+$: 525.1102; Found 525.1108

Product 3kd

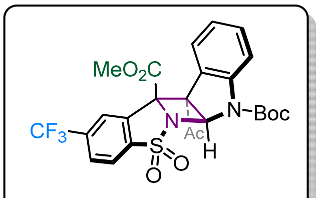

A white solid, 29.3 mg, 53% yield.

TLC: R_f = 0.57 (Hexane/EtOAc = 3:1) [UV].

^{1}H NMR (400 MHz, $CDCl_3$): δ 8.39 (s, 1H), 8.05 (br s, 1H), 7.99 – 7.91 (m, 2H), 7.50 – 7.42 (m, 2H), 7.14 (td, J = 7.6, 1.1 Hz, 1H), 6.50 (s, 1H), 3.58 (s, 3H), 2.19 (s, 3H), 1.65 (s, 9H).

^{13}C NMR (101 MHz, $CDCl_3$): δ 198.3, 166.5, 150.4, 144.3, 140.2, 135.7, 135.5(q, J = 30.1 Hz), 131.8, 128.9(q, J = 270.9 Hz), 125.9 (q, J = 3.8 Hz), 124.32, 123.6, 122.9, 121.52, 117.1, 83.9, 79.9, 79.6, 66.6, 53.6, 28.3, 27.6.

HRMS (ESI): Calcd for $C_{25}H_{24}F_3N_2O_7S$ $[M+H]^+$: 553.1251; Found 553.1259.

Product 3ke

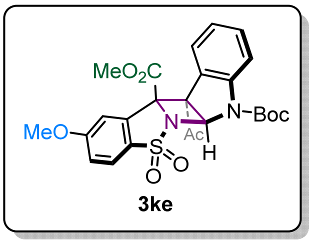

A white solid, 44.2 mg, 86% yield.

TLC: R_f = 0.51 (Hexane/EtOAc = 3:1) [UV].

^{1}H NMR (400 MHz, $CDCl_3$): δ 8.02 (br s, 1H), 7.72 (d, J = 8.6 Hz, 1H), 7.51 (d, J = 8.6 Hz, 1H), 7.46 – 7.38 (m, 2H), 7.16 (dd, J = 8.6, 2.3 Hz, 1H), 7.13 – 7.08 (m, 1H), 6.53 (s, 1H), 3.95 (s, 3H), 3.55 (s, 3H), 2.23 (s, 3H), 1.64 (s, 9H).

^{13}C NMR (101 MHz, $CDCl_3$): δ 198.6, 167.1, 163.8, 150.6, 144.3, 135.9, 131.5, 129.0, 126.2, 124.1, 123.3, 118.9, 116.9, 111.9, 83.6, 79.6, 79.4, 66.4, 56.1, 53.4, 28.3, 28.1.

HRMS (ESI): Calcd for $C_{25}H_{27}N_2O_8S$ $[M+H]^+$: 515.1483; Found 515.1486.

Product 3kf

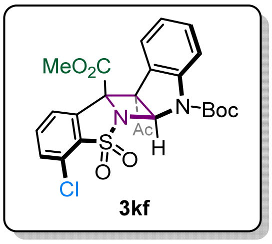

A white solid, 21.7 mg, 42% yield.

TLC: R_f = 0.37 (Hexane/EtOAc = 3:1) [UV].

^{1}H NMR (400 MHz, $CDCl_3$): δ 8.03 (br s, 1H), 7.96 (d, J = 7.7 Hz, 1H), 7.68 – 7.58 (m, 2H), 7.52 – 7.39 (m, 2H), 7.16 – 7.08 (m, 1H), 6.55 (s, 1H), 3.55 (s, 3H), 2.21 (s, 3H), 1.64 (s, 9H).

^{13}C NMR (101 MHz, $CDCl_3$): δ 198.5, 166.7, 150.5, 144.2, 135.9, 135.0, 134.8, 132.4, 131.6, 129.9, 126.9, 126.2, 123.9, 123.5, 116.9, 83.9, 79.6, 79.0, 66.5, 53.5, 28.3, 28.0.

HRMS (ESI): Calcd for $C_{24}H_{24}ClN_2O_7S$ $[M+H]^+$: 519.0987; Found 519.0991.

Product 3kg

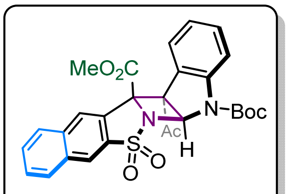

A white solid, 47.6 mg, 89% yield.

TLC: R_f = 0.38 (Hexane/EtOAc = 3:1) [UV].

^{1}H NMR (400 MHz, $CDCl_3$): δ 8.47 (s, 1H), 8.37 (s, 1H), 8.15 – 7.92 (m, 3H), 7.75 – 7.66 (m, 2H), 7.62 (d, J = 7.8 Hz, 1H), 7.49 – 7.41 (m, 1H), 7.19 – 7.09 (m, 1H), 6.59 (s, 1H), 3.59 (s, 3H), 2.27 (s, 3H), 1.65 (s, 9H).

^{13}C NMR (101 MHz, $CDCl_3$): δ 198.6, 167.5, 150.6, 144.3, 135.3, 134.0, 133.7, 131.4, 129.4, 129.2, 129.1, 128.8, 128.7, 128.5, 126.3, 124.3, 123.4, 123.0, 117.0, 83.6, 79.9, 79.3, 66.8, 53.4, 28.3, 28.1.

HRMS (ESI): Calcd for $C_{28}H_{27}N_2O_7S$ $[M+H]^+$: 535.1533; Found 535.1543.

Product 3kh

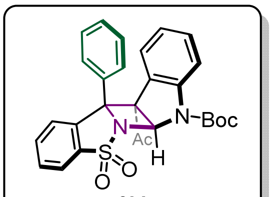

A white solid, 43.7 mg, 87% yield.

TLC: R_f = 0.51 (Hexane/EtOAc = 3:1) [UV].

^{1}H NMR (400 MHz, $CDCl_3$): δ 8.04 (d, J = 8.1 Hz, 1H), 7.88 (s, 1H), 7.80 – 7.70 (m, 3H), 7.68 – 7.62 (m, 1H), 7.55 (t, J = 7.1 Hz, 1H), 7.30 – 7.13 (m, 4H), 6.98 (d, J = 7.1 Hz, 1H), 6.80 – 6.72 (m, 1H), 6.59 (s, 1H), 2.00 (s, 3H), 1.69 (s, 9H).

^{13}C NMR (101 MHz, $CDCl_3$): δ 202.0, 150.8, 143.4, 140.3, 137.7, 135.9, 133.6, 130.6, 130.5, 129.5, 129.3, 128.8, 128.4, 128.0, 127.1, 125.6, 125.2, 123.0, 122.2, 116.3, 83.5, 83.4, 80.1, 68.1, 28.4, 28.4.

HRMS (ESI): Calcd for $C_{28}H_{27}N_2O_5S$ $[M+H]^+$: 503.1635; Found 503.1638.

Product 3ki

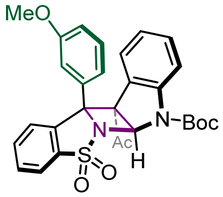

3ki

A white solid, 37.1 mg, 67% yield.

TLC: R_f = 0.55 (Hexane/EtOAc = 3:1) [UV].

^{1}H NMR (400 MHz, $CDCl_3$): δ 8.03 (d, J = 8.2 Hz, 1H), 7.89 (s, 1H), 7.78 (d, J = 8.2 Hz, 1H), 7.69 – 7.62 (m, 1H), 7.62 – 7.54 (m, 1H), 7.38 (d, J = 8.2 Hz, 1H), 7.27 – 7.16 (m, 3H), 7.03 (d, J = 8.2 Hz, 1H), 6.84 – 6.78 (m, 1H), 6.73 – 6.66 (m, 1H), 6.58 (s, 1H), 3.73 (s, 3H), 2.00 (s, 3H), 1.69 (s, 9H).

^{13}C NMR (101 MHz, $CDCl_3$): δ 201.9, 159.5, 150.8, 143.5, 140.2, 139.2, 135.9, 133.5, 130.6, 130.4, 129.5, 128.9, 127.1, 125.3, 123.1, 122.1, 117.9, 116.3, 113.7, 111.3, 83.5, 83.3, 80.0, 68.1, 55.3, 28.4, 28.3.

HRMS (ESI): Calcd for $C_{29}H_{29}N_2O_6S$ $[M+H]^+$: 555.1560; Found 555.1571.

Product 3kj

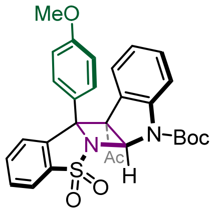

3kj

A white solid, 48.9 mg, 92% yield.

TLC: R_f = 0.55 (Hexane/EtOAc = 3:1) [UV].

^{1}H NMR (400 MHz, $CDCl_3$): δ 7.99 (d, J = 8.1 Hz, 1H), 7.89 (br s, 1H), 7.76 (d, J = 8.1 Hz, 1H), 7.69 – 7.59 (m, 3H), 7.59 – 7.51 (m, 1H), 7.25 – 7.20 (m, 1H), 7.00 – 6.95 (m, 1H), 6.82 – 6.74 (m, 3H), 6.56 (s, 1H), 3.70 (s, 3H), 1.99 (s, 3H), 1.68 (s, 9H).

^{13}C NMR (101 MHz, $CDCl_3$): δ 202.2, 159.2, 150.8, 143.4, 140.6, 135.8, 133.5, 130.5, 130.3, 129.8, 128.7, 127.1, 127.0, 125.4, 123.1, 122.2, 116.3, 113.7, 83.5, 83.2, 80.0, 67.9, 55.2, 28.3, 25.6.

HRMS (ESI): Calcd for $C_{29}H_{29}N_2O_6S$ $[M+H]^+$: 555.1560; Found 555.1566.

Product 3kk

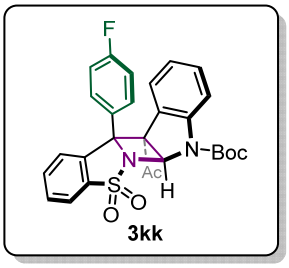

A white solid, 40.7 mg, 75% yield.

TLC: R_f = 0.59 (Hexane/EtOAc = 3:1) [UV].

^{1}H NMR (400 MHz, $CDCl_3$): δ 8.00 (d, *J* = 8.1 Hz, 1H), 7.90 (br s, 1H), 7.78 (d, *J* = 8.1 Hz, 1H), 7.75 – 7.64 (m, 3H), 7.61 – 7.54 (m, 1H), 7.26 – 7.21 (m, 1H), 6.97 – 6.90 (m, 3H), 6.82 – 6.77 (m, 1H), 6.58 (s, 1H), 1.99 (s, 3H), 1.68 (s, 9H).

^{13}C NMR (101 MHz, $CDCl_3$): δ 201.9, 162.3 (d, *J* = 247.6 Hz), 150.7, 143.4, 140.1 (d, *J* = 10.9 Hz), 139.6, 137.3, 135.9, 133.6(d, *J* = 8.5 Hz), 130.6 (d, J = 12.5 Hz), 128.6, 127.7 (d, *J* = 8.4 Hz), 126.9, 125.2, 123.1, 122.2, 116.4, 115.3 (d, *J* = 21.6 Hz), 83.5, 82.8, 80.1, 68.12, 28.32.

HRMS (ESI): Calcd for $C_{28}H_{25}FN_2NaO_5S$ $[M+Na]^+$: 543.1360; Found 543.1368.

Product 3kl

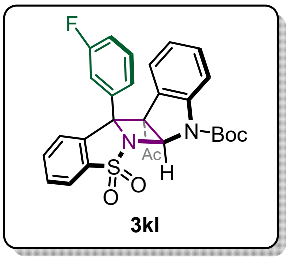

A white solid, 49.4 mg, 91% yield.

TLC: R_f = 0.60 (Hexane/EtOAc = 3:1) [UV].

^{1}H NMR (400 MHz, $CDCl_3$): δ 8.00 (d, *J* = 8.1 Hz, 1H), 7.89 (br s, 1H), 7.79 – 7.69 (m, 3H), 7.60 – 7.54 (m, 1H), 7.28 – 7.21 (m, 1H), 6.97 – 6.90 (m, 3H), 6.83 – 6.76 (m, 1H), 6.58 (s, 1H), 1.97 (s, 3H), 1.68 (s, 9H).

^{13}C NMR (101 MHz, $CDCl_3$): δ 202.1 162.3 (d, *J* = 247.6 Hz), 150.7, 143.4, 140.1, 135.9, 133.6 (d, *J* = 8.5 Hz), 130.6 (d, *J* = 15.8 Hz), 129.7 (d, *J* = 8.4 Hz), 128.7, 127.7 (d, *J* = 8.4 Hz), 126.9, 125.2, 123.2, 122.2, 116.4, 115.8 (d, *J* = 21.6 Hz), 115.4 (d, *J* = 21.6 Hz), 83.6, 82.9, 80.1, 68.1, 28.4, 28.3.

HRMS (ESI): Calcd for $C_{28}H_{25}FN_2NaO_5S$ $[M+Na]^+$: 543.1360; Found 543.1365.

Product 5a

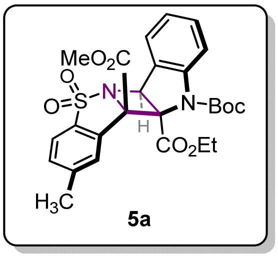

A white solid, 50.1 mg, 90% yield.

TLC: Rf = 0.56 (Hexane/EtOAc = 3:1) [UV]

^{1}H NMR (400 MHz, $CDCl_3$): δ 7.85 (s, 1H), 7.72 (q, J = 8.0 Hz, 1H), 7.51 – 7.44 (t, 2H), 7.43 – 7.38 (td, 1H), 7.16 (t, J = 7.5, 1.0 Hz, 1H), 5.73 (s, 1H), 3.68 (s, 3H), 2.54 (s, 3H), 1.65 (s, 9H), 1.02 (t, J = 7.1 Hz, 3H).

^{13}C NMR (151 MHz, $CDCl_3$) δ 166.5, 165.1, 150.7, 145.1, 143.7, 134.3, 133.2, 132.2, 130.9, 129.7, 126.3, 125.7, 124.0, 121.3, 115.3, 83.3, 80.9, 71.4, 69.9, 62.2, 53.6, 28.1, 21.9, 13.7.

HRMS (ESI): $[M+Na]^+$ Calcd for $C_{26}H_{28}N_2NaO_8S$ 551.1459; Found 551.1453.

Product 5b

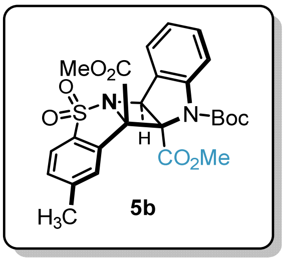

A white solid, 45.3 mg, 88% yield.

TLC: R_f = 0.39(Hexane/EtOAc = 3:1) [UV].

^{1}H NMR (400 MHz, $CDCl_3$): δ 7.93 (br s, 1H), 7.85 (s, 1H), 7.72 (d, *J* = 7.9 Hz, 1H), 7.52 – 7.37 (m, 3H), 7.16 (t, *J* = 7.9 Hz, 1H), 5.74 (s, 1H), 3.69 (s, 3H), 3.56 (s, 3H), 2.55 (s, 3H), 1.64 (s, 9H).

^{13}C NMR (101 MHz, $CDCl_3$): δ 166.5, 165.6, 150.6, 145.0, 143.6, 133.9, 133.1, 132.3, 131.0, 129.9, 126.3, 125.7, 124.1, 121.3, 115.4, 83.6, 80.9, 71.1, 69.9, 53.6, 52.7, 28.1, 21.9.

HRMS (ESI): Calcd for $C_{25}H_{27}N_2O_8S$ $[M+H]^+$: 515.1483; Found 515.1478.

Product 5c

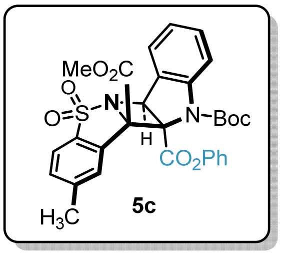

A white solid, 49.1 mg, 85% yield.

TLC: R_f = 0.43 (Hexane/EtOAc = 3:1) [UV].

^{1}H NMR (600 MHz, $CDCl_3$): δ 7.93 (s, 2H), 7.71 (d, J = 8.1 Hz, 1H), 7.53 (d, J = 7.9 Hz, 1H), 7.47 – 7.43 (m, 2H), 7.34 – 7.29 (m, 2H), 7.20 (q, J = 7.9 Hz, 2H), 6.92 (d, J = 7.9 Hz, 2H), 5.96 (s, 1H), 3.73 (s, 3H), 2.50 (s, 3H), 1.71 (s, 9H).

^{13}C NMR (151 MHz, $CDCl_3$): δ 166.5, 163.6, 151.0, 149.8, 144.9, 143.9, 133.7, 133.1, 132.5, 131.1, 129.9, 129.5, 126.4, 125.9, 124.2, 121.4, 120.9, 115.5, 84.2, 80.9, 71.3, 69.8, 53.7, 28.2, 21.9.

HRMS (ESI): Calcd for $C_{30}H_{29}N_2O_8S$ $[M+H]^+$: 577.1639 ; Found 577.1633.

Product 5d

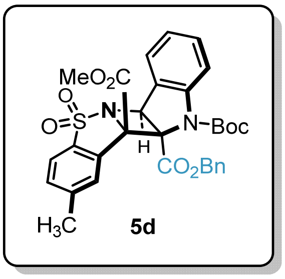

A white solid, 39.5 mg, 67% yield.

TLC: R_f = 0.41 (Hexane/EtOAc = 3:1) [UV].

^{1}H NMR (400 MHz, $CDCl_3$): δ 7.90 (br s, 1H), 7.79 (s, 1H), 7.64 (d, J = 7.9 Hz, 1H), 7.48 – 7.34 (m, 3H), 7.28 (dt, J = 7.9, 3.1 Hz, 3H), 7.15 (t, J = 7.9 Hz, 1H), 7.10 – 7.05 (m, 2H), 5.76 (s, 1H), 5.03 (d, J = 12.3 Hz, 1H), 4.90 (d, J = 12.3 Hz, 1H), 3.67 (s, 3H), 2.45 (s, 3H), 1.56 (s, 9H).

^{13}C NMR (101 MHz, $CDCl_3$): δ 166.5, 165.0, 150.7, 145.1, 143.7, 134.1, 133.1, 132.2, 131.0, 129.4, 128.6, 128.6, 128.3, 126.2, 125.8, 124.0, 121.2, 115.3, 83.6, 80.8, 71.56, 69.9, 67.9, 53.6, 28.0, 21.9.

HRMS (ESI): Calcd for $C_{31}H_{31}N_2O_8S$ $[M+H]^+$: 591.1796 ; Found 591.1791.

Product 5e

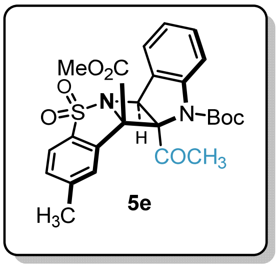

A white solid, 37.9 mg, 76% yield.

TLC: R_f = 0.47 (Hexane/EtOAc = 3:1) [UV].

^{1}H NMR (600 MHz, $CDCl_3$): δ 8.01 (br s, 1H), 7.80 (s, 1H), 7.69 (d, *J* = 8.0 Hz, 1H), 7.50 (d, *J* = 7.9 Hz, 1H), 7.48 – 7.40 (m, 2H), 7.20 (t, *J* = 7.9 Hz, 1H), 5.64 (s, 1H), 3.65 (s, 3H), 2.53 (s, 3H), 1.94 (s, 3H), 1.65 (s, 9H).

^{13}C NMR (151 MHz, $CDCl_3$): δ 197.1, 166.9, 150.3, 144.7, 143.5, 133.9, 132.7, 132.1, 131.2, 129.5, 126.6, 126.2, 124.5, 121.3, 115.5, 84.4, 80.5, 74.9, 68.9, 53.5, 28.1, 24.8, 21.9.

HRMS (ESI): Calcd for $C_{25}H_{27}N_2O_7S$ $[M+H]^+$: 499.1533; Found 499.1330.

Product 5f

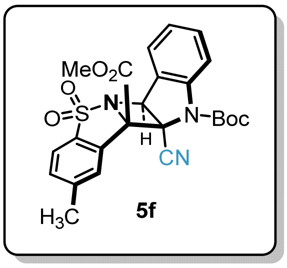

A white solid, 34.7 mg, 72% yield.

TLC: R_f = 0.42 (Hexane/EtOAc = 3:1) [UV].

^{1}H NMR (600 MHz, $CDCl_3$): δ 7.90 (s, 1H), 7.80 (d, *J* = 8.1 Hz, 1H), 7.61 (d, *J* = 8.1 Hz, 1H), 7.49 – 7.44 (m, 2H), 7.21 (d, *J* = 8.1 Hz, 1H), 5.87 (s, 1H), 3.78 (s, 3H), 2.61 (s, 3H), 1.74 (s, 9H).

^{13}C NMR (151 MHz, $CDCl_3$): δ 164.5, 145.5, 143.9, 133.6, 133.5, 131.6, 129.1, 125.7, 125.1, 124.7, 121.9, 115.7, 112.9, 85.4, 79.3, 71.1, 62.8, 54.1, 28.1, 22.1.

HRMS (ESI): Calcd for $C_{24}H_{24}N_3O_6S$ $[M+H]^+$: 482.1380; Found 482.1374.

Product 5g

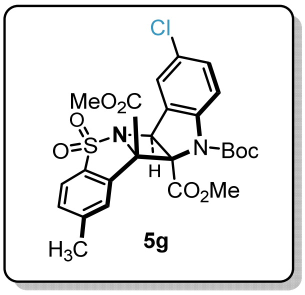

A yellow solid, 45.5 mg, 83% yield.

TLC: R_f = 0.48 (Hexane/EtOAc = 3:1) [UV].

^{1}H NMR (400 MHz, $CDCl_3$): δ 7.86 (br s, 1H), 7.83 (s, 1H), 7.71 (d, J = 8.1 Hz, 1H), 7.50 (d, J = 8.1 Hz, 1H), 7.42 (d, J = 2.3 Hz, 1H), 7.37 (dd, J = 8.1, 2.3 Hz, 1H), 5.68 (s, 1H), 3.70 (s, 3H), 3.57 (s, 3H), 2.55 (s, 3H), 1.63 (s, 9H).

^{13}C NMR (101 MHz, $CDCl_3$): δ 166.3, 165.2, 150.4, 143.8, 133.7, 133.0, 132.4, 130.9, 129.9, 129.1, 127.9, 125.8, 121.3, 116.4, 84.0, 80.9, 71.3, 69.1, 53.7, 52.8, 28.0, 21.9.

HRMS (ESI): Calcd for $C_{25}H_{26}ClN_2O_8S$ $[M+H]^+$: 549.1093 ; Found 549.1087.

Product 5h

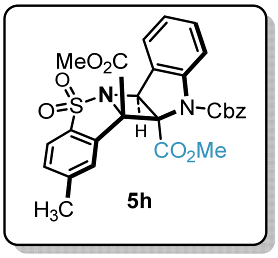

A yellow solid, 33.4 mg, 61% yield.

TLC: R_f = 0.38 (Hexane/EtOAc = 3:1) [UV].

^{1}H NMR (400 MHz, $CDCl_3$): δ 7.98 (br s, 1H), 7.69 (d, J = 8.1 Hz, 1H), 7.52 – 7.36 (m, 9H), 7.20 (t, J = 8.1 Hz, 1H), 5.73 (s, 1H), 5.44 (d, J = 11.6 Hz, 1H), 5.22 (d, J = 11.6 Hz, 1H), 3.61 (s, 3H), 3.37 (s, 3H), 2.41 (s, 3H).

^{13}C NMR (101 MHz, $CDCl_3$): δ 166.5, 165.3, 144.6, 143.8, 134.9, 133.8, 133.2, 132.2, 131.1, 129.1, 129.0, 128.9, 128.7, 126.2, 125.9, 124.5, 121.3, 115.3, 80.4, 71.2, 69.9, 68.8, 53.6, 52.8, 21.8.

HRMS (ESI): Calcd for $C_{28}H_{25}N_2O_8S$ $[M+H]^+$: 549.1326 ; Found 549.1322.

Product 5i

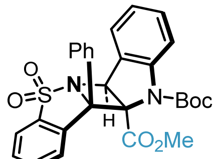

5i

A white solid, 36.3 mg, 70% yield.

TLC: R_f = 0.33 (Hexane/EtOAc = 3:1) [UV].

^{1}H NMR (400 MHz, $CDCl_3$): δ 8.13 (d, *J* = 7.9 Hz, 1H), 7.83 – 7.70 (m, 5H), 7.62 (t, *J* = 7.6 Hz, 1H), 7.49 (d, *J* = 7.6 Hz, 1H), 7.36 (t, *J* = 7.6 Hz, 1H), 7.29 – 7.25 (m, 3H), 7.15 (t, *J* = 7.6 Hz, 1H), 5.75 (s, 1H), 3.58 (s, 3H), 1.44 (s, 9H).

^{13}C NMR (101 MHz, $CDCl_3$): δ 166.8, 151, 144.9, 139.3, 136.6, 135.4, 132.1, 130.7, 130.2, 129.9, 128.5, 128.0, 127.36, 127.2, 125.4, 123.8, 121.8, 115.4, 84.7, 83.1, 72.8, 68.8, 52.5, 28.0.

HRMS (ESI): Calcd for $C_{28}H_{27}N_2O_6S$ $[M+H]^+$: 519.1584 ; Found 519.1579.

Product 6a

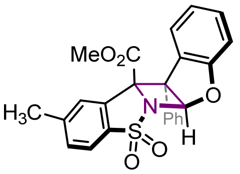

6a

A white solid, 41.9 mg, 92% yield.

TLC: R_f = 0.59 (Hexane/EtOAc = 3:1) [UV].

^{1}H NMR (400 MHz, $CDCl_3$): δ 7.68 – 7.63 (m, 2H), 7.48 – 7.43 (m, 2H), 7.36 – 7.30 (m, 4H), 7.28 – 7.23 (m, 1H), 7.16 (s, 1H), 7.10 – 7.03 (m, 2H), 6.56 (s, 1H), 3.65 (s, 3H), 2.23 (s, 3H).

^{13}C NMR (101 MHz, $CDCl_3$): δ 168.3, 159.2, 144.4, 135.1, 134.8, 132.1, 130.8, 128.8, 128.4, 128.3, 127.5, 126.4, 122.5, 121.7, 112.5, 96.9, 79.8, 63.2, 53.3, 21.5.

HRMS (ESI): Calcd for $C_{24}H_{19}NNaO_5S$ $[M+H]^+$: 456.0876; Found 456.0876.

Product 6b

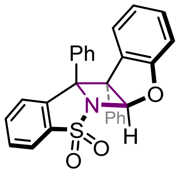

6b

A white solid, 36.8 mg, 80% yield.

TLC: R_f = 0.56 (Hexane/EtOAc = 3:1) [UV].

^{1}H NMR (400 MHz, $CDCl_3$): δ 7.77 (d, *J* = 8.1 Hz, 1H), 7.64 – 7.59 (m, 2H), 7.51 – 7.42 (m, 1H), 7.38 – 7.26 (m, 9H), 7.24 – 7.15 (m, 1H), 7.02 (d, *J* = 8.1 Hz, 2H), 7.01 – 6.95 (m, 1H), 6.78 – 6.69 (m, 1H), 6.61 (s, 1H).

^{13}C NMR (101 MHz, $CDCl_3$): δ 159.2, 141.1, 137.9, 137.1, 135.7, 132.5, 130.0, 129.9, 128.9, 128.6, 128.3, 128.1, 127.6, 127.5, 127.3, 127.0, 125.4, 122.3, 121.8, 111.9, 96.9, 82.4, 64.5.

HRMS (ESI): Calcd for $C_{27}H_{19}NNaO_3S$ $[M+Na]^+$: 460.0976; Found 460.0978.

Product 6c

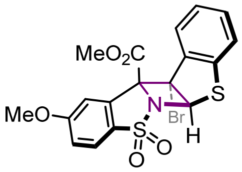

6c

A white solid, 21.1 mg, 43% yield.

TLC: R_f = 0.57 (Hexane/EtOAc = 3:1) [UV].

^{1}H NMR (400 MHz, $CDCl_3$): δ 7.75 (d, *J* = 8.1 Hz, 1H), 7.61 – 7.57 (m, 2H), 7.41 – 7.35 (m, 1H), 7.34 – 7.30 (m, 1H), 7.28 – 7.22 (m, 2H), 5.93 (s, 1H), 4.02 (s, 3H), 3.58 (s, 3H)

^{13}C NMR (101 MHz, $CDCl_3$): δ 166.2, 163.7, 140.4, 137.5, 136.7, 130.9, 128.1, 127.8, 125.9, 123.5, 119.2, 113.2, 83.2, 81.1, 68.2, 65.0, 56.2, 53.4.

HRMS (ESI): Calcd for $C_{18}H_{14}BrNNaO_5S_2$ $[M+Na]^+$: 489.9389; Found 489.9392.

Product 6d

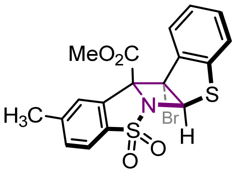

6d

A white solid, 23.0 mg, 51% yield.

TLC: R_f = 0.53 (Hexane/EtOAc = 3:1) [UV].

^{1}H NMR (400 MHz, $CDCl_3$): δ 7.97 (s, 1H), 7.75 (d, J = 8.1 Hz, 1H), 7.60 – 7.54 (m, 2H), 7.42 – 7.36 (m, 1H), 7.31 (d, J = 8.1 Hz, 1H), 7.28 – 7.24 (m, 1H), 5.90 (s, 1H), 3.58 (s, 3H), 2.61 (s, 3H).

^{13}C NMR (101 MHz, $CDCl_3$): δ 166.2, 144.8, 140.4, 136.7, 135.1, 133.2, 133.1, 131.4, 129.6, 128.1, 125.9, 123.5, 122.0, 83.5, 81.0, 64.9, 53.5, 22.1.

HRMS (ESI): Calcd for $C_{18}H_{15}BrNO_4S_2$ $[M+H]^+$: 451.9620; Found 451.9618.

Product 6e

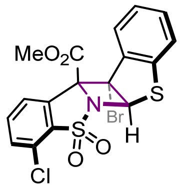

6e

A white solid, 28.1 mg, 57% yield.

TLC: R_f = 0.49 (Hexane/EtOAc = 3:1) [UV].

^{1}H NMR (400 MHz, $CDCl_3$): δ 8.13 – 8.09 (m, 1H), 7.78 (t, J = 7.9 Hz, 1H), 7.72 – 7.68 (m, 1H), 7.59 – 7.55 (m, 1H), 7.42 – 7.37 (m, 1H), 7.35 – 7.31 (m, 1H), 7.28 – 7.25 (m, 1H), 5.97 (s, 1H), 3.57 (s, 3H).

^{13}C NMR (101 MHz, $CDCl_3$): δ 165.8, 140.3, 137.3, 136.4, 134.6, 134.1, 132.7, 131.6, 129.9, 128.0, 127.9, 126.0, 123.6, 82.5, 81.2, 64.8, 53.6.

HRMS (ESI): Calcd for $C_{17}H_{12}BrClNNaO_4S_2$ $[M+Na]^+$: 493.8892; Found 493.8894.

Product 6f

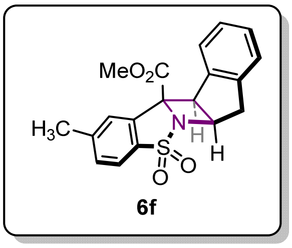

A white solid, 32.8 mg, 87% yield.

TLC: R_f = 0.63 (Hexane/EtOAc = 3:1) [UV].

^{1}H NMR (400 MHz, $CDCl_3$): δ 7.82 (s, 1H), 7.71 (d, *J* = 7.9 Hz, 1H), 7.48 (d, *J* = 7.9 Hz, 1H), 7.43 (d, *J* = 7.9 Hz, 1H), 7.40 – 7.28 (m, 3H), 4.87 (t, *J* = 6.3 Hz, 1H), 4.15 (d, *J* = 6.3 Hz, 1H), 3.54 (s, 3H), 3.50 (d, *J* = 17.5 Hz, 1H), 3.28 (dd, *J* = 17.5, 6.3 Hz, 1H), 2.57 (s, 3H).

^{13}C NMR (101 MHz, $CDCl_3$): δ 167.7, 145.3, 143.4, 138.8, 137.6, 133.9, 132.1, 129.1, 127.1, 126.5, 126.4, 126.1, 122.1, 79.0, 65.7, 52.8, 52.4, 40.0, 21.9.

HRMS (ESI): Calcd for $C_{19}H_{17}NNaO_4S$ $[M+Na]^+$: 378.0770; Found 378.0778.

Product 6g

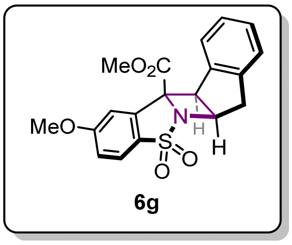

A white solid, 23.2 mg, 59% yield.

TLC: R_f = 0.58 (Hexane/EtOAc = 3:1) [UV].

^{1}H NMR (400 MHz, $CDCl_3$): δ 7.73 (d, *J* = 8.6 Hz, 1H), 7.45 – 7.41 (m, 2H), 7.39 – 7.26 (m, 3H), 7.17 (dd, *J* = 8.6, 2.3 Hz, 1H), 4.90 (t, *J* = 6.3 Hz, 1H), 4.16 (d, *J* = 6.3 Hz, 1H), 3.98 (s, 3H), 3.54 (s, 3H), 3.50 (d, *J* = 17.5 Hz, 1H), 3.28 (dd, *J* = 17.5, 6.3 Hz, 1H).

^{13}C NMR (101 MHz, $CDCl_3$): δ 167.6, 164.3, 143.4, 141.1, 137.5, 129.1, 128.6, 127.1, 126.5, 126.1, 123.7, 118.2, 109.8, 78.7, 65.8, 56.2, 52.9, 52.4, 40.0..

HRMS (ESI): Calcd for $C_{19}H_{17}NNaO_5S$ $[M+Na]^+$: 394.0720; Found 394.0721.

3.4 Gram-scale reaction

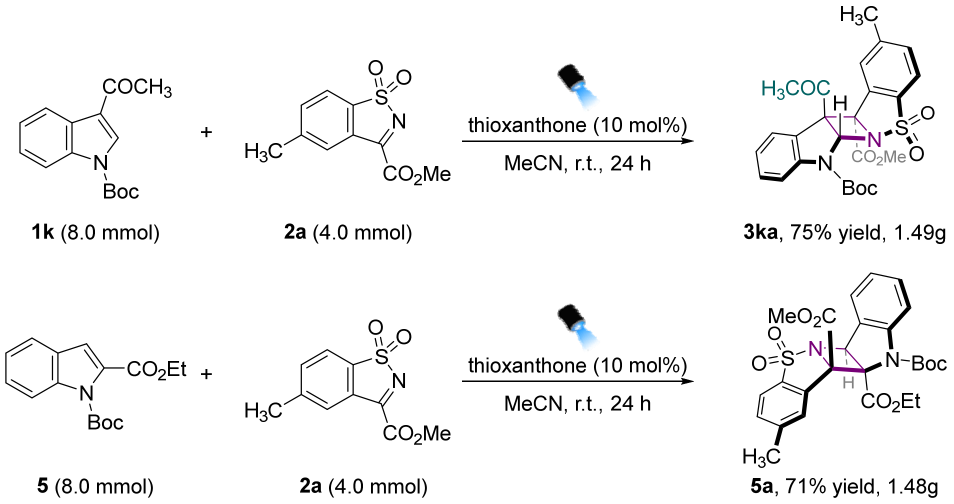

To a 200 mL flask equipped with a magnetic stirring bar and rubber seal containing a stirring bar was added indole **1k** or **5** (8.0 mmol), imine **2a** (4.0 mmol) in MeCN (80 mL). After the addition of thioxanthone (10 mol%), the reaction mixture was deoxygenated by bubbling N_2 for 10 min, and was then illuminated under 20 W blue LEDs (λ_{max} = 405 nm) at room temperature for 24 h. The mixture was then concentrated in vacuo and purified by silica gel flash chromatography (PE/EA: 15/1 to 4/1) to afford the corresponding azetidines as a white solid.

3.5 Mechanistic studies

3.5.1 UV/Vis absorption spectra

UV/Vis absorption specta were recorded on a Varioskan LUX (Thermo). Samples were prepared in MeCN with **1k** (2.5 mM), **2a** (2.5 mM), **1k** + **2a** (2.5 mM), **1k** + **2a** (2.5 mM), and thioxanthone (0.1 mM). The photocatalyst is the only species absorbing at 405 nm.

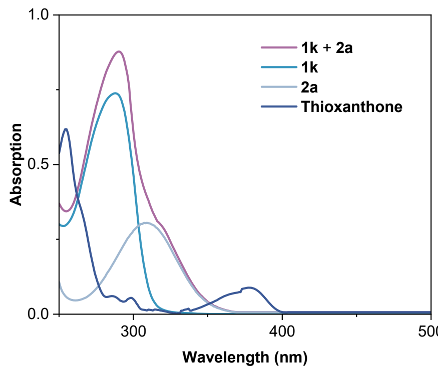

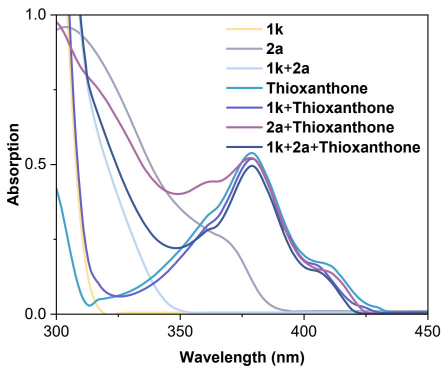

Supplementary Fig. 4. Left: UV-vis absorption spectrum of 1**k** (2.5 mM), **2a** (2.5 mM), **1k** + **2a** (2.5 mM) and thioxanthone (0.1 mM). Right: UV-vis absorption spectrum of

1**k** (0.5 mM), **2a** (0.5 mM), thioxanthone (0.5 mM) and all of their mixtures.

3.5.2 Electrochemical measurements

The reduction/oxidation potentials were determined by cyclic voltammograms of each compound on a potentiostat. Conditions: glassy carbon as the working electrode, Pt plate as the counter electrode, Ag/AgCl (0.1 M in MeCN) as the reference electrode, tetrabutylammonium hexafluorophosphate (Bu_4NBF_4) in MeCN (0.1 M) was used as the supporting electrolyte, scan rate = 100 mV·s^{-1}. The redox potentials of the substrates were measured in MeCN (0.1 M) with the supporting electrolyte, respectively. The potential ($E_{p/2}$) were determined according to literature procedures[12]. For comparison with reported redox potentials of the photosensitizers, the reference electrode potential was converted to the saturated calomel electrode (SCE) scale using the formula: $E_{SCE} = E_{Ag/AgCl}$ -0.034 V. No obvious oxidation peak of **1k** and **2a** was observed before +1.7 V vs SCE, which suggest that these two compounds could not be oxidized by the excited state *thioxanthone ($E_{1/2}^{[PC]*/[PC]-}$ = +1.18 V vs SCE). Similarly, no obvious reduction peak of **1k** was observed before -1.2 V vs SCE. **1k** could not be reduced by the *thioxanthone ($E_{1/2}^{[PC]+/[PC]*}$= -1.11 V vs SCE). Only **2a** may be reduced by the the *thioxanthone ($E_{1/2}^{[PC]+/[PC]*}$= -1.11 V vs SCE). However, comparing these values with those of the utilized photosensitizers, there was no obvious correlation between the oxidation/reduction potentials (Supplementary Fig. 5). Moreover, there appeared to be a clear correlation between the triplet energy values of photocatalysts and the reactivity of the photocycloaddition. These results collectively indicate a single-electron transfer mechanism is unlikely involved.

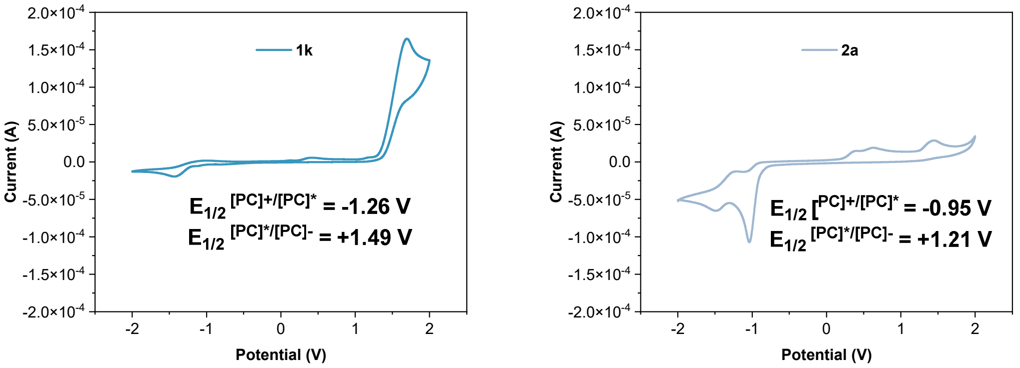

Supplementary Fig. 5. Cyclic voltammogram of substrate **1k** and **2a**.

Supplementary Table 5. Comparison of triplet excited state energies and redox potentials for phtocatalysts.

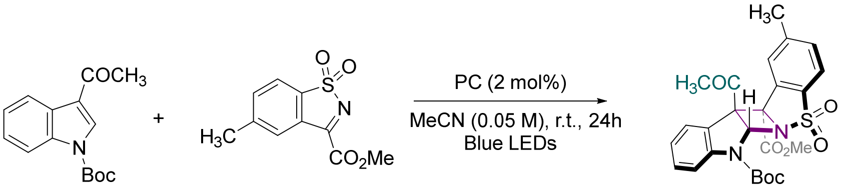

1k 2a 3ka

Entry[a]	Photocatalyst	E_T (kcal mol^{-1})	$E_{1/2}$ [PC]+/[PC]* (V)	$E_{1/2}$ [PC]*/[PC]- (V)	Yield (%)[b]
1	$[MesAcr](ClO_4)$	44.7	-	+0.57	0
2	$[Ru(bpy)_3](PF6)_2$	46.5	-0.81	+0.77	0
3	$[Ir(ppy)_2(dtbbpy)](PF_6)$	49.2	-0.96	+0.66	0
4	fac-$[Ir(ppy)_3]$	58.1	-1.73	+0.31	Trace
5	$[Ir(dF(CF_3)ppy)_2(dtbbpy)](PF_6)$	61.8	-0.89	+1.21	92
**6[c]**	**Thioxanthone**	**65.5**	**-1.11**	**+1.18**	**91**

[a]Reaction conditions: A solution of **1k** (0.2 mmol), **2a** (0.1 mmol) and photosensitizer in MeCN (2 mL) was irradiated by a 10 W purple LEDs (λ_{max} = 455 nm) at room temperature under nitrogen for 24 h. [b]Isolated yield of **3ka**. [c]Catalyst loading (10 mol%) was used with 405 nm LEDs.

3.5.3 Stern-Volmer quenching studies

Stern-Volmer luminescence quenching analysis was conducted using a Shimadzu RF-5301PC spectrofluorometer. The solution of thioxanthone (0.05 mM in MeCN) was excited at λ_{ex} = 350 nm and the emission was collected at 390 nm. The substrates **1k** and **2a** were dissolved in MeCN (300 mM), respectively. For each quenching experiment, 1μL of the stock solution was titrated to a solution (1 mL) of thioxanthone in a 1-cm quartz cuvette, , which was sealed with a septum-equipped cap and degassed by sparging with nitrogen gas for 5 min. The addition of 10 μL stock solution refers to an increase in the quencher concentration of 3.0 mM. The emission was observed at 390 nm and the ratio of I_0/I was plotted as a function of the quencher concentration (I_0: emission intensity without quencher; I: emission intensity with quencher). The Stern-Volmer analysis shows that imine **2a** is the main quencher of the photocatalyst thioxanthone with a quenching rate K_{SV} = 0.029 mM^{-1}, while the quenching rate of **1k** is K_{SV} = 0.003 mM^{-1}.

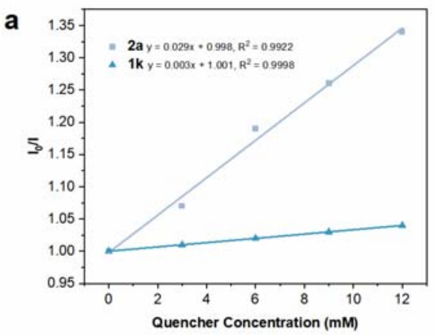

b

[1k] (mM)	I_0/I	[2a] (mM)	I_0/I
0.0	1.00	0.0	1.00
3.0	1.008	3.0	1.07
6.0	1.019	6.0	1.19
9.0	1.021	9.0	1.26
12.0	1.029	12.0	1.34

Supplementary Fig. 6. a, Stern-Volmer plots. **b**, Data was obtained from the photosensitizer thioxanthone using **1k** and **2a** as quenchers in MeCN.

3.5.4 Photocycloaddition via direct excitation

General procedure: To a 10 mL tube containing a stirring bar was added indole **1k** (0.2 mmol), imine **2a** (0.1 mmol) in MeCN (2 mL). The reaction mixture was deoxygenated by bubbling N_2 for 10 min, and was then illuminated under 10 W corresponding LEDs at room temperature for 24 h. The mixture was then concentrated in vacuum and purified by silica gel flash chromatography (PE/EA: 15/1 to 4/1) to afford the corresponding azetidine **3ka**.

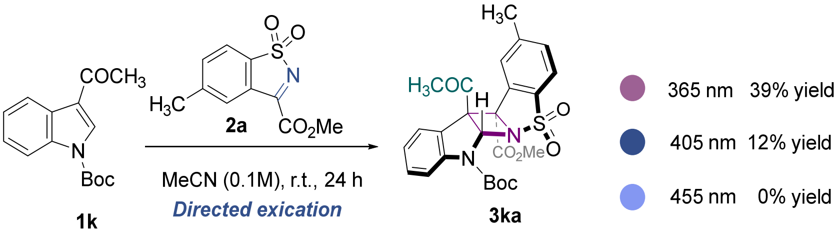

Supplementary Fig. 7. Light control reactions. No product formation was observed in the absence of light. With the change of wavelength, the yields of the reactions sharply decreased (12% yield, λ_{max} = 405 nm) or vanished (0% yield, λ_{max} = 455 nm).

3.5.5 Triplet energy quenching experiments

2,5-Dimethylhexa-2,4-diene is known as a triplet quencher[13]. The standard reaction was prominently inhibited by using one equiv of 2,5-dimethylhexa-2,4-diene as a triplet quencher hinting towards the involvement of excited triplet state intermediates in this intermolecular dearomative aza-Games Paternò-Buchi reaction.

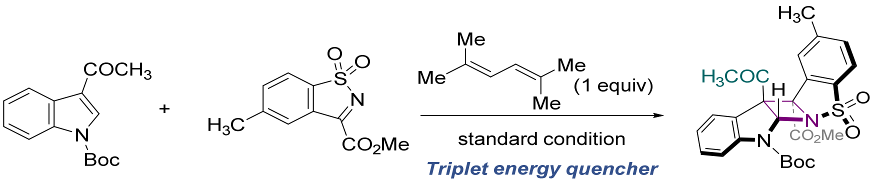

Supplementary Fig. 8. Triplet energy quenching experiments using one equiv of 2,5-

dimethylhexa-2,4-diene. Results indicated the involvement of excited triplet state intermediates.

4.Computational results

4.1 Calculation details

All density functional calculations were carried out using the B3LYP-D3[14-15] functional (with Grimme's D3 dispersion) and performed in the Gaussian 16 program[16]. The 6-31G(d,p) basis set was used for geometry optimization. Frequency calculations at the same level of theory have been performed to identify that all the stationary points are local minima (no imaginary frequencies) or transition states (one imaginary frequency) and to provide free energy corrections at 298 K. The final and solvation energies for the fully optimized structures in the acetonitrile were calculated by employing the SMD[17] continuum solvation model with the larger 6-311+G(2d,2p) basis set.

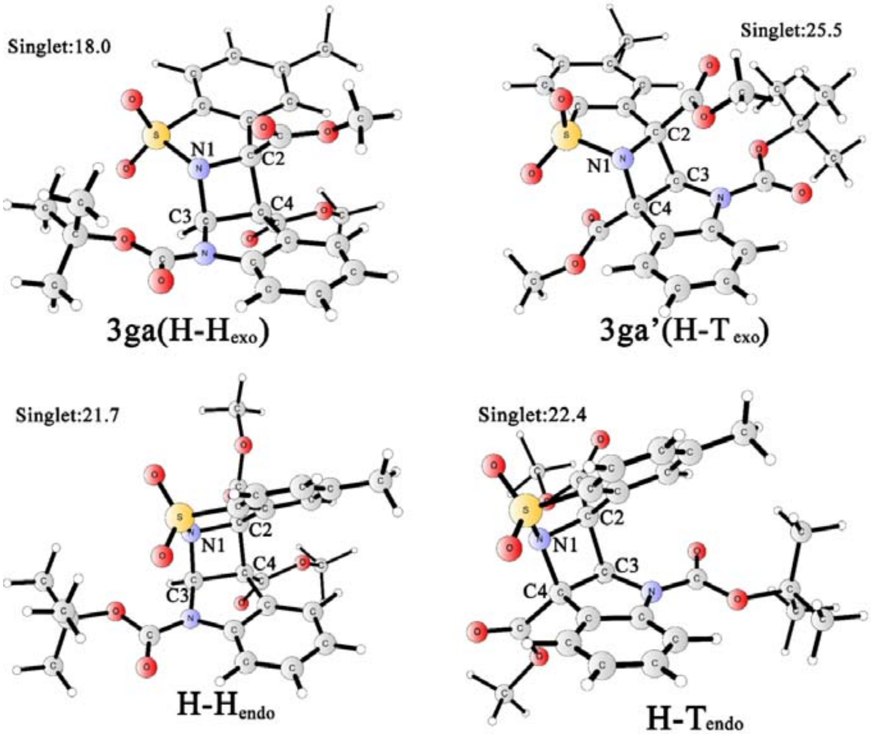

Supplementary Fig. 9. Optimized structures of **3ga(H-H*exo*)**, **H-H*endo***, **3ga'(H-T*exo*)** and **H-T*endo***.

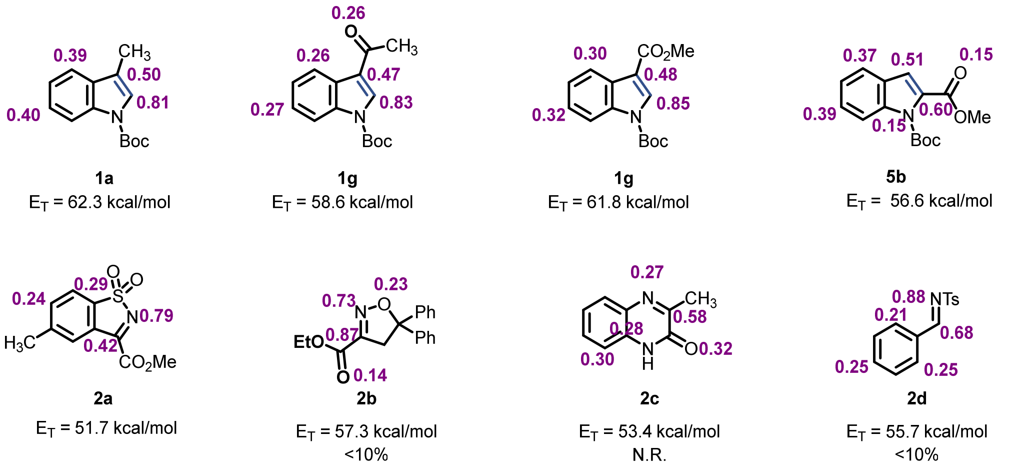

Supplementary Fig. 10. Calculated Mulliken spin population of the selected substrate (T1) and their calculated triple-singlet energy gaps (at the (U)B3LYP-D3/6-311+G(2d,2p)/SMD(acetonitrile) level of theory).

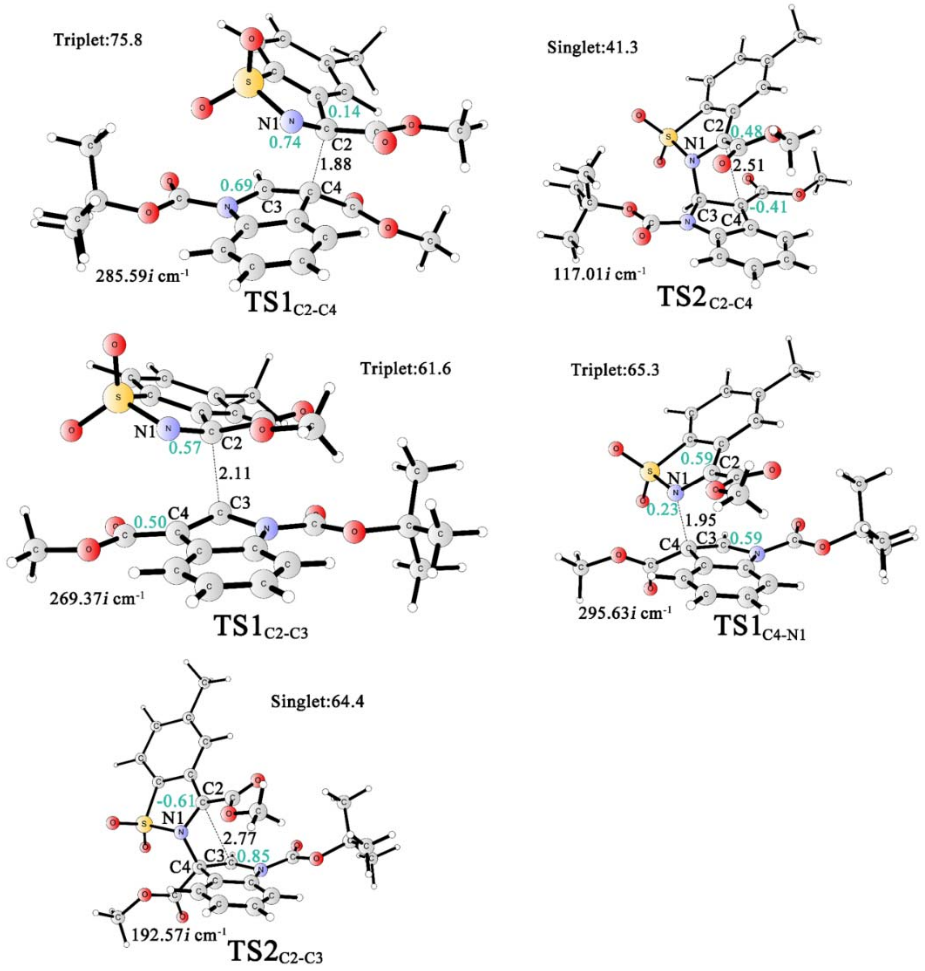

Supplementary Fig. 11. Optimized key transition state structures of **3ga**(H-H*exo*) and **3ga'**(H-T*exo*), respectively. Distances are given in Å. The imaginary frequencies for transition states are also shown. The Mulliken spin densities on key atoms are shown in cyan.

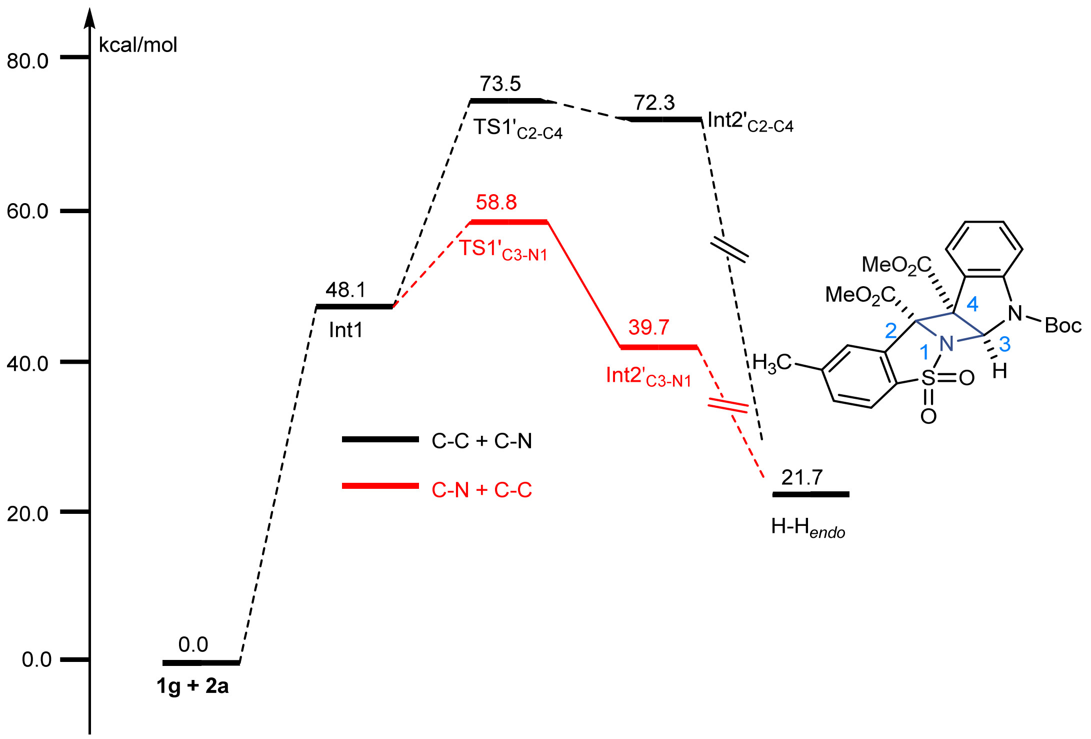

Supplementary Fig. 12. Calculated energy profile of H-Hendo. Computational method: B3LYP-D3/6-311+G(2d, 2p)/SMD(acetonitrile).

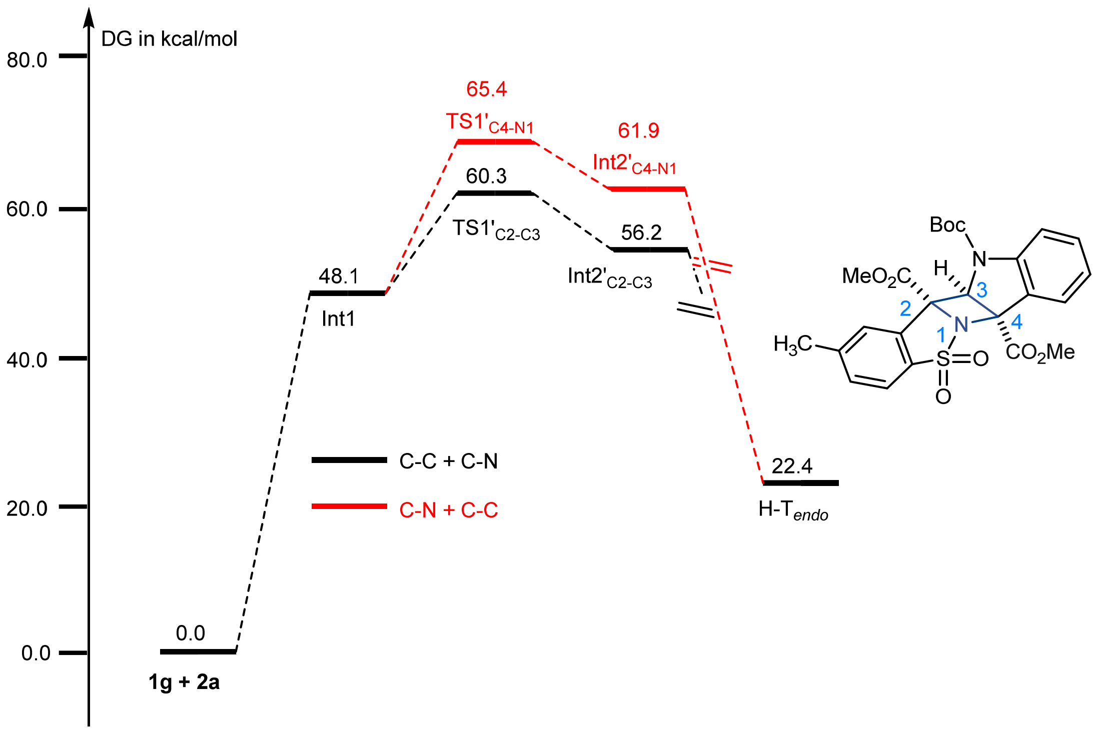

Supplementary Fig. 13. Calculated energy profile of **H-T*endo***. Computational method: B3LYP-D3/6-311+G(2d, 2p)/SMD(acetonitrile).

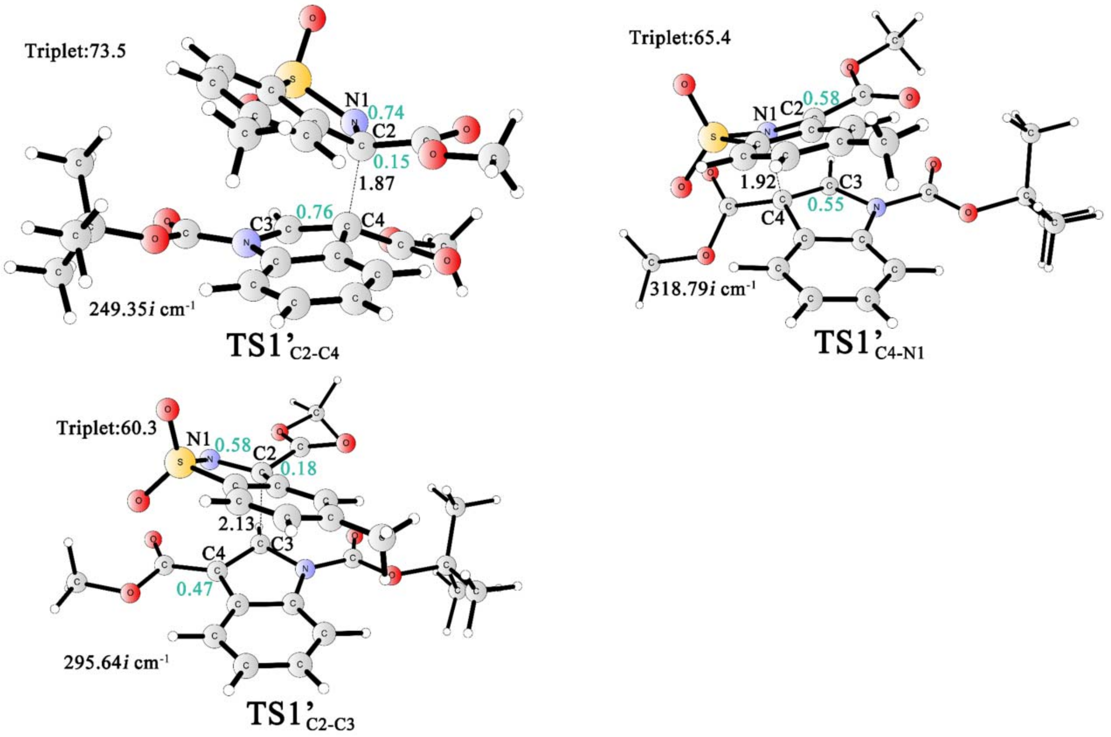

Supplementary Fig. 14. Optimized key transition state structures of **H-H*endo*** and **H-T*endo***, respectively. Distances are given in Å. The imaginary frequencies for transition states are also shown. The Mulliken spin densities on key atoms are shown in cyan.

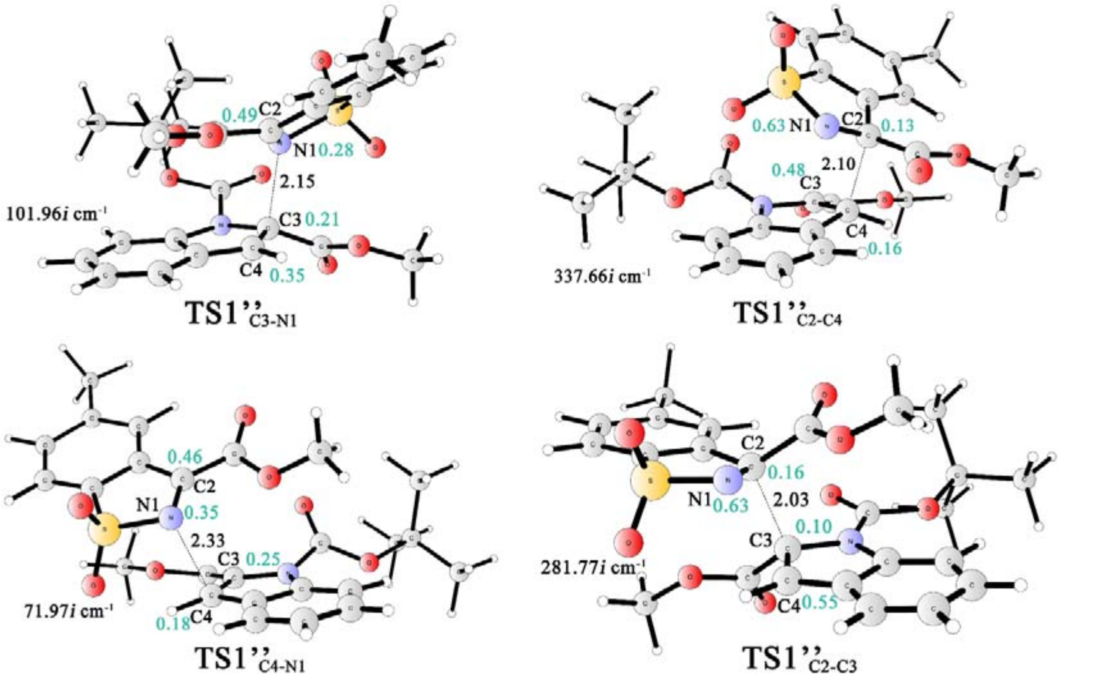

Supplementary Fig. 15. Optimized key transition state structures for the model reaction of N-Boc methyl indole-2-carboxylate **5b** with imine **2a**. respectively. Distances are given in Å. The imaginary frequencies for transition states are also shown. The Mulliken spin densities on key atoms are shown in cyan.

4.2 Optimized structures

Supplementary Table 6. Optimized structures.

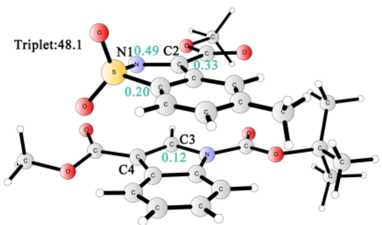

Int1

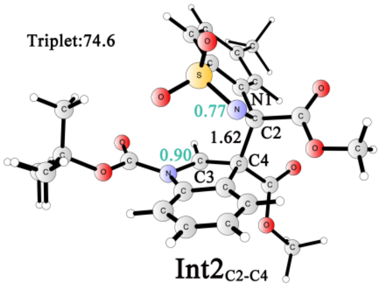

$Int2_{C2\text{-}C4}$

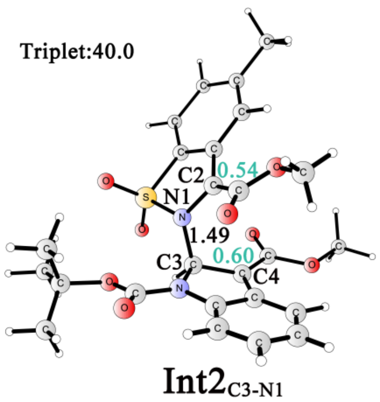

$Int2_{C3\text{-}N1}$

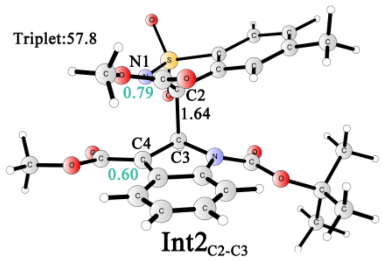

$Int2_{C2\text{-}C3}$

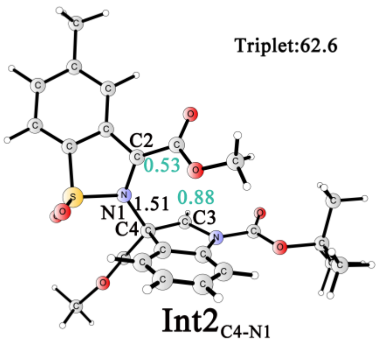

$Int2_{C4\text{-}N1}$

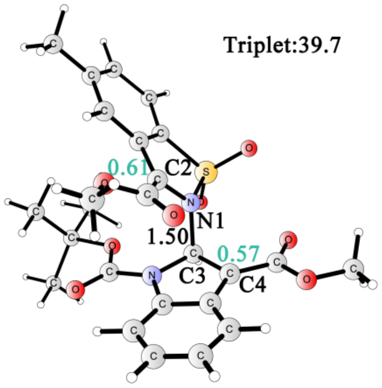

$Int2'_{C3\text{-}N1}$

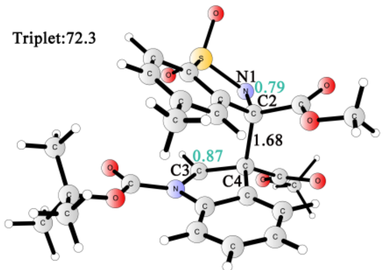

$Int2'_{C2\text{-}C4}$

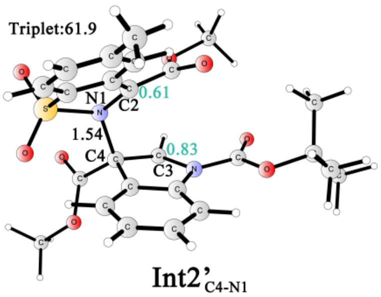

$Int2'_{C4\text{-}N1}$

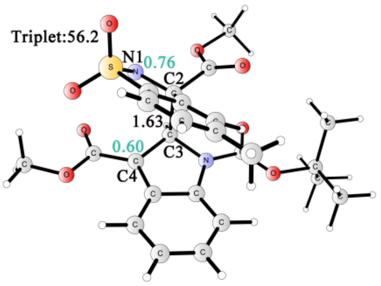

$Int2'_{C2\text{-}C3}$

4.3 Tables of energies

Supplementary Table 7. Calculated energies in Hartree and imaginary frequencies.

Stationary point	Geometry optimization energy (Hartree)	Gibbs energy correction energy (Hartree)	Large basis energy (Hartree)	Imaginary frequencies ()
**1g**	-937.5952881	0.25271	-937.8762189	
**2a**	-1140.280281	0.137281	-1140.573936	
**Int1**	-2077.827926	0.412712	-2078.393225	
**TS1$_{C3-N1}$**	-2077.821032	0.412598	-2078.384425	92*i*
**TS1$_{C2-C4}$**	-2077.787977	0.413107	-2078.349434	285.59*i*
**TS1$_{C2-C3}$**	-2077.811142	0.413563	-2078.372487	269.37*i*
**TS1$_{C4-N1}$**	-2077.804773	0.412062	-2078.365189	295.63*i*
**TS1'$_{C3-N1}$**	-2077.817446	0.414912	-2078.378276	287.33*i*
**TS1'$_{C2-C4}$**	-2077.791225	0.412015	-2078.352085	249.35*i*
**TS1'$_{C2-C3}$**	-2077.813417	0.413631	-2078.374698	295.64*i*
**TS1'$_{C4-N1}$**	-2077.806814	0.413425	-2078.366399	318.79*i*
**Int2$_{C3-N1}$**	-2077.851827	0.414754	-2078.408215	
**Int2$_{C2-C4}$**	-2077.79118	0.412183	-2078.350447	
**Int2$_{C2-C3}$**	-2077.815074	0.413631	-2078.378602	
**Int2$_{C4-N1}$**	-2077.8125	0.412668	-2078.370077	
**Int2'$_{C3-N1}$**	-2077.85087	0.414776	-2078.408568	
**Int2'$_{C2-C4}$**	-2077.79221	0.412103	-2078.354076	
**Int2'$_{C2-C3}$**	-2077.824572	0.415147	-2078.382691	
**Int2'$_{C4-N1}$**	-2077.813838	0.413391	-2078.371831	
**TS2$_{C2-C4}$**	-2077.851072	0.417112	-2078.408447	117.01*i*
**TS2$_{C2-C3}$**	-2077.810286	0.413417	-2078.36794	192.57*i*
**3ga(H-H$_{exo}$)**	-2077.886658	0.418315	-2078.446767	
**3ga'(H-T$_{exo}$)**	-2077.882037	0.420879	-2078.4374	
**H-H$_{emdo}$**	-2077.880529	0.418326	-2078.44083	
**H-T$_{endo}$**	-2077.877972	0.419096	-2078.440498	
**TS1''$_{C3-N1}$**	-2077.796777	0.412343	-2078.360114	101.96*i*
**TS1''$_{C2-C4}$**	-2077.786307	0.412074	-2078.349898	337.66*i*
**TS1''$_{C4-N1}$**	-2077.808484	0.412059	-2078.376464	71.97*i*
**TS1''$_{C2-C3}$**	-2077.793731	0.411386	-2078.35276	281.77*i*

4.4 PMI analysis for 3D score

The normalized principal moment of inertia (MPI)[18] values can be used to describe the 3-dimensional shape of a molecule. RDKit[19] is an open-source cheminformatics toolkit that can be used to evaluate these structures. These values were used to determine the normalized PMI values, I_1/I_3 and I_2/I_3.

Supplementary Table 8. The calculated normalized MPI values for select molecules.

Molecular	I_1/I_3	I_2/I_3	3D score (I_1/I_3 + I_2/I_3)
6b	0.71	0.88	1.59
5i	0.74	0.82	1.56
3ca	0.66	0.86	1.52
3da	0.64	0.86	1.5
5a	0.71	0.79	1.5
5d	0.71	0.79	1.5
3ya	0.58	0.91	1.49
6d	0.56	0.89	1.45
6a	0.63	0.81	1.44
3ba	0.59	0.75	1.34
3ja	0.54	0.75	1.29
6f	0.42	0.85	1.27
3ga	0.52	0.74	1.26
3aa	0.49	0.68	1.17
3kd	0.39	0.78	1.17
3oa	0.4	0.7	1.1
Hexadiyne	0.00	1.00	1.00
Benzene	0.50	0.50	1.00
Adamantane	1.00	1.00	2.00

5. Supplementary references

1. Zhang, J., Wu, M., Fan, J., Xu, Q. & Xie, M. Selective C-H acylation of indoles with alpha-oxocarboxylic acids at the C4 position by palladium catalysis. *Chem. Commun.* **55**, 8102-8105 (2019).
2. Mambrini, A., Gori, D., Guillot, R., Kouklovsky, C. & Alezra, V. Oxidative coupling of enolates using memory of chirality: an original enantioselective synthesis of quaternary alpha-amino acid derivatives. *Chem. Commun.* **54**, 12742-12745 (2018).
3. Tang, S. B. *et al.* Rhodium(III)-catalyzed C4-amidation of indole-oximes with dioxazolones via C-H activation. *Org. Biomol. Chem.* **18**, 7922-7931 (2020).
4. Zhang, X. *et al.* Photocatalyzed transfer hydrogenation and deuteriation of cyclic N-sulfonylimines. *Org. Chem. Front.* **6**, 2410-2414 (2019).
5. Li, Y. *et al.* Copper(II)-catalyzed asymmetric photoredox reactions: enantioselective alkylation of imines driven by visible light. *J. Am. Chem. Soc.* **140**, 15850-15858 (2018).
6. Li, Y., Lei, M. & Gong, L. Photocatalytic regio- and stereoselective C(sp^3)–H functionalization of benzylic and allylic hydrocarbons as well as unactivated alkanes. *Nat. Catal.* **2**, 1016-1026 (2019).
7. Pan, J. *et al.* Highly enantioselective synthesis of fused tri- and tetrasubstituted aziridines: aza-Darzens reaction of cyclic imines with alpha-lalogenated ketones catalyzed by bifunctional phosphonium salt. *Angew. Chem. Int. Ed.* **58**, 7425-7430 (2019).
8. Li, L., Matsuo, B., Levitre, G., McClain,E. J., Voight, E. A., Crane, E. A., & Molander, G. A. Dearomative intermolecular [2 + 2] photocycloaddition for construction of C(sp(^{3}))-rich heterospirocycles on-DNA. *Chem. Sci.* **14**, 2713-2720 (2023).
9. Oderinde, M. S.*et al.* Photocatalytic dearomative intermolecular [2 + 2] cycloaddition of heterocycles for building molecular complexity. *J. Org. Chem.* **86**, 1730-1747 (2021).
10. Romero, N. A. & Nicewicz, D. A. Organic photoredox catalysis. *Chem. Rev.* **116**, 10075–10166 (2016).
11. Teegardin, K., Day, J. I., Chan, J. & Weaver, J. Advances in photocatalysis: a microreview of visible light mediated ruthenium and iridium catalyzed organic transformations. *Org. Process Res. Dev.* **20**, 1156–1163 (2016).
12. Roth, H. G., Romero, N. A. & Nicewicz, D. A. Experimental and calculated electrochemical potentials of common organic molecules for applications to single-electron redox chemistry. *Synlett.* **27**, 714–723 (2016).
13. *Handbook of Photochemistry*, 3rd ed, Montalti, M.; Credi, A.; Prodi, L.; Gandolfi, M. T., Ed.; CRC, Taylor & Francis Group, Boca Raton, FL(2006).
14. Becke, A.D. Density-functional thermochemistry. III. The role of exact exchange. *The J. Chem. Phys.* **98**, 5648-5652 (1993).

15. Grimme, S., Antony, J., Ehrlich, S. & Krieg, H. A consistent and accurate ab initio parametrization of density functional dispersion correction (DFT-D) for the 94 elements H-Pu. *J. Chem. Phys.* **132**, 154104 (2010).
16. Frisch, M. J. *et al. Gaussian 16 Rev. A.01*, Wallingford, CT (2016).
17. Marenich, A. V., Cramer, C. J. & Truhlar, D. G., Universal solvation model based on solute electron density and on a continuum model of the solvent defined by the bulk dielectric constant and atomic surface tensions. *J. Phys. Chem. B* **113,** 6378-6396 (2010).
18. Sauer, W. H. B. & Schwarz, M. K. Molecular Shape Diversity of Combinatorial Libraries: A Prerequisite for Broad Bioactivity. *J. Chem. Inf. Comput. Sci.* **243,** *43,* 987-1003 (2003).
19. RDKit: Open-source cheminformatics. https://www.rdkit.org.

6. NMR spectra

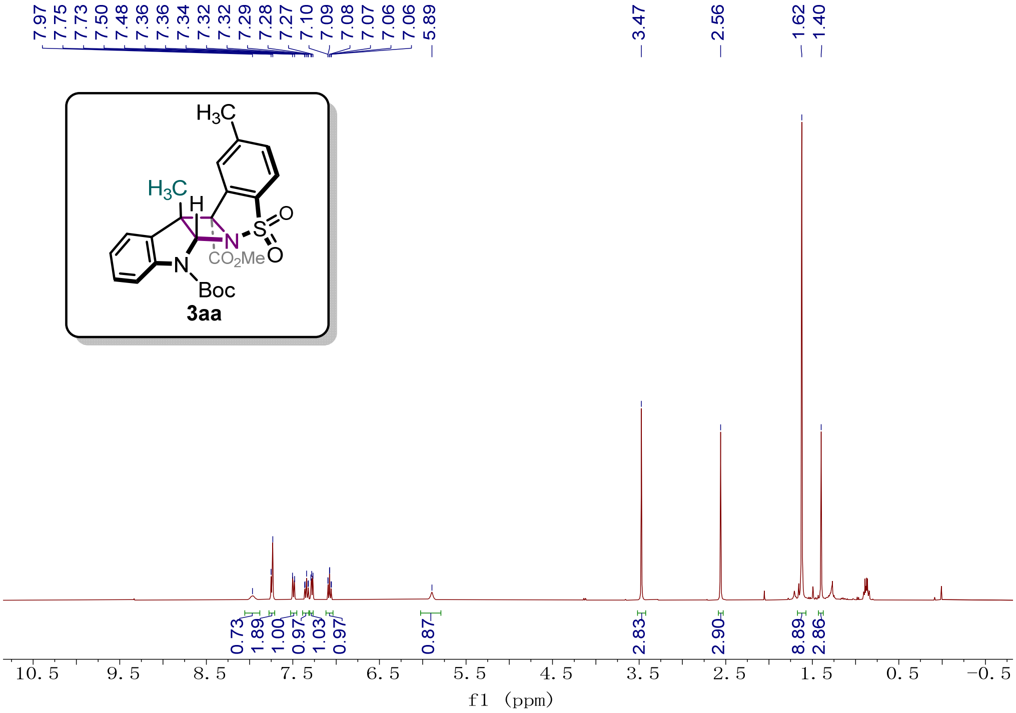

Supplementary Fig. 16. ^{1}H NMR of compound **3aa (400 MHz, $CDCl_3$)**

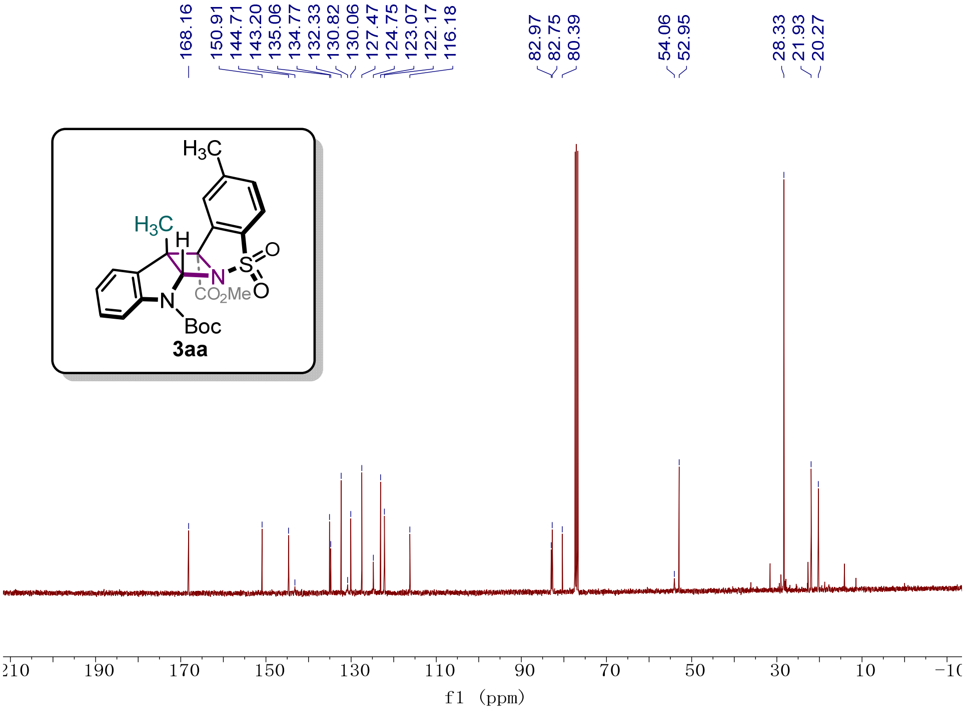

Supplementary Fig. 17. ^{13}C NMR of compound **3aa (101 MHz, $CDCl_3$)**

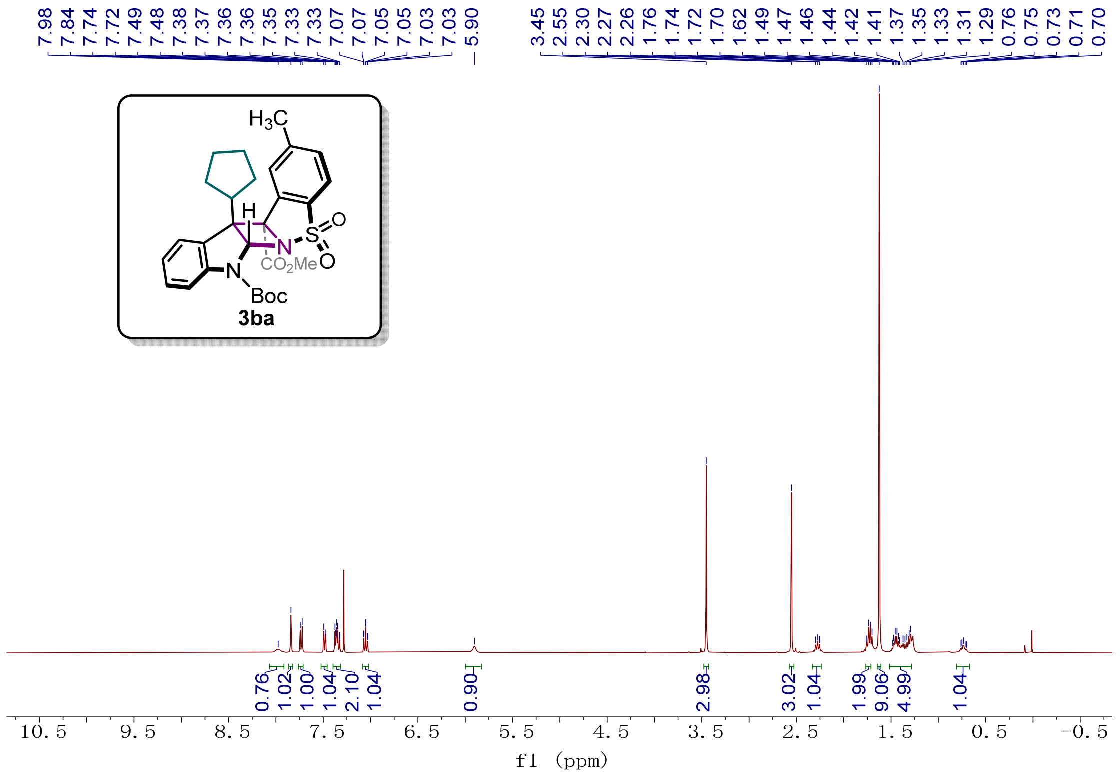

Supplementary Fig. 18. ^{1}H NMR of compound **3ba (400 MHz, CDCl$_3$)**

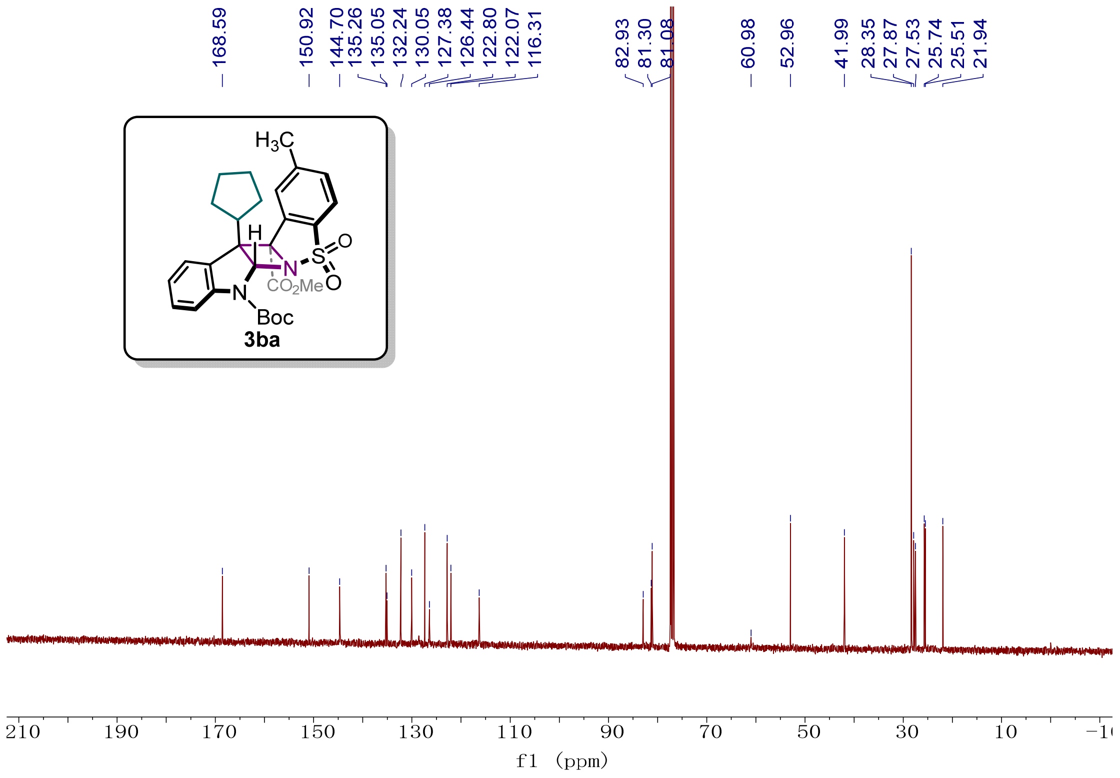

Supplementary Fig. 19. ^{13}C NMR of compound **3ba (101 MHz, CDCl$_3$)**

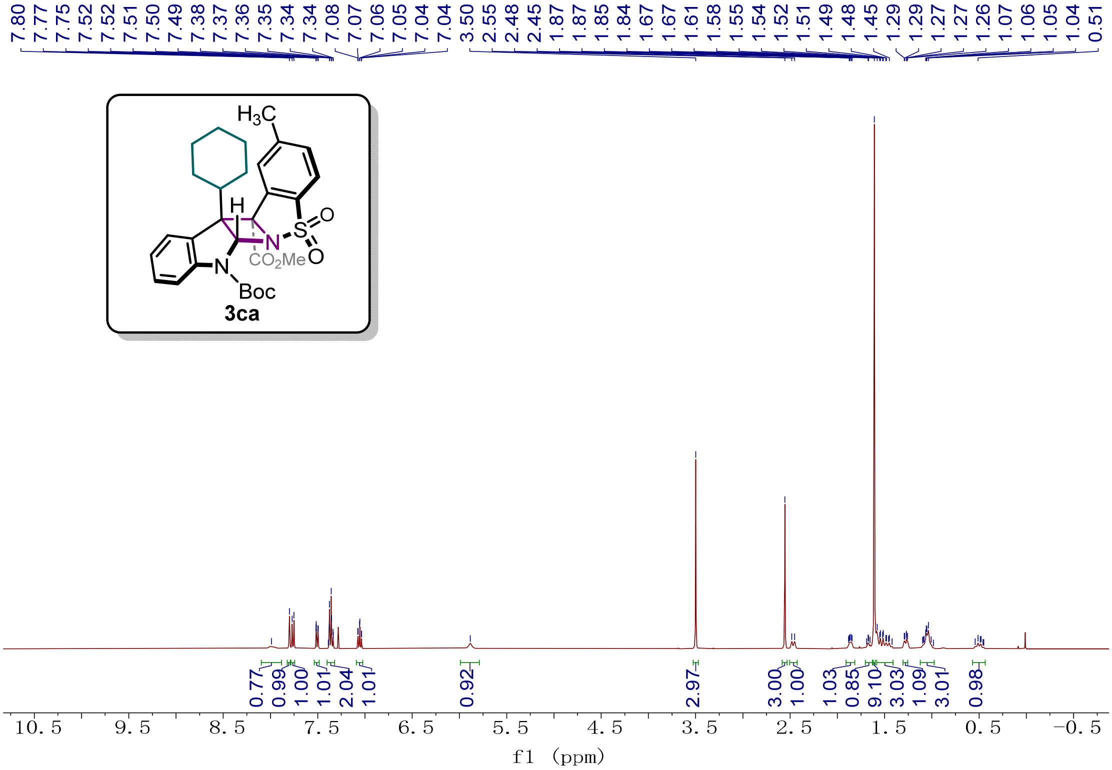

Supplementary Fig. 20. ^{1}H NMR of compound **3ca (400 MHz, CDCl$_3$)**

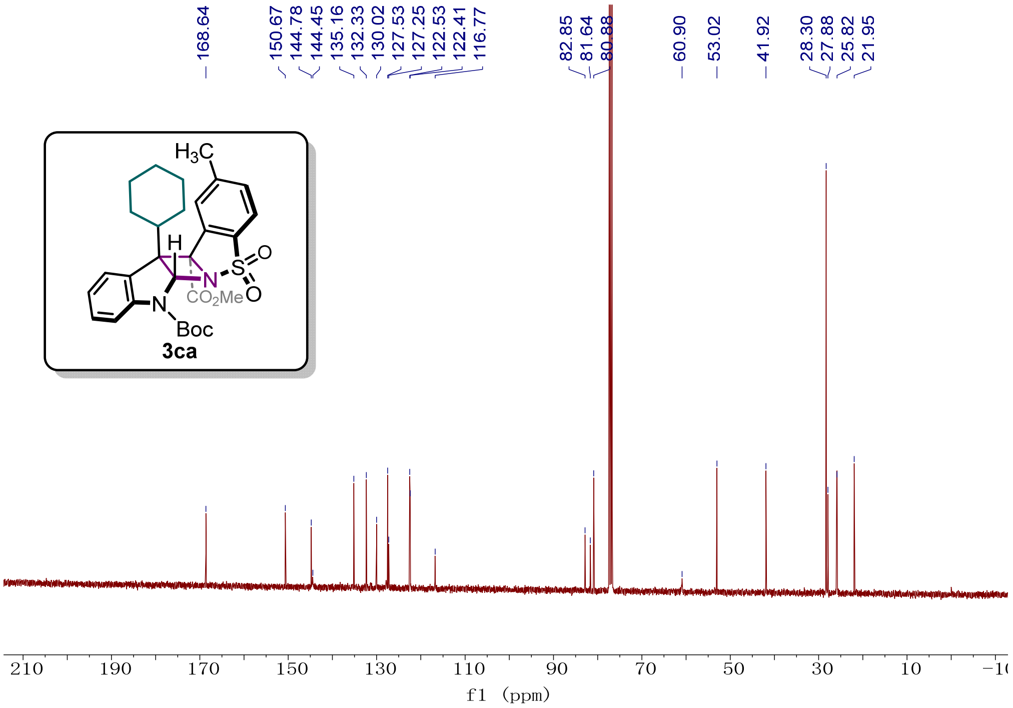

Supplementary Fig. 21. ^{13}C NMR of compound **3ca (101 MHz, CDCl$_3$)**

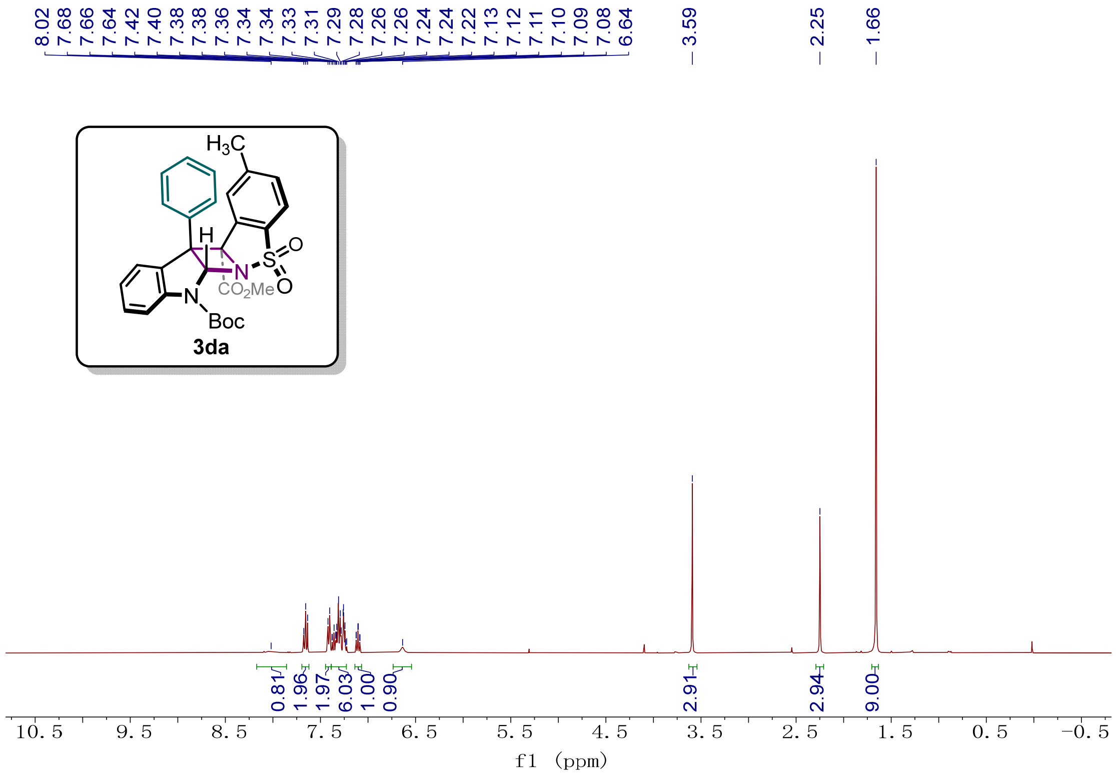

Supplementary Fig. 22. ^{1}H NMR of compound **3da (400 MHz, CDCl$_3$)**

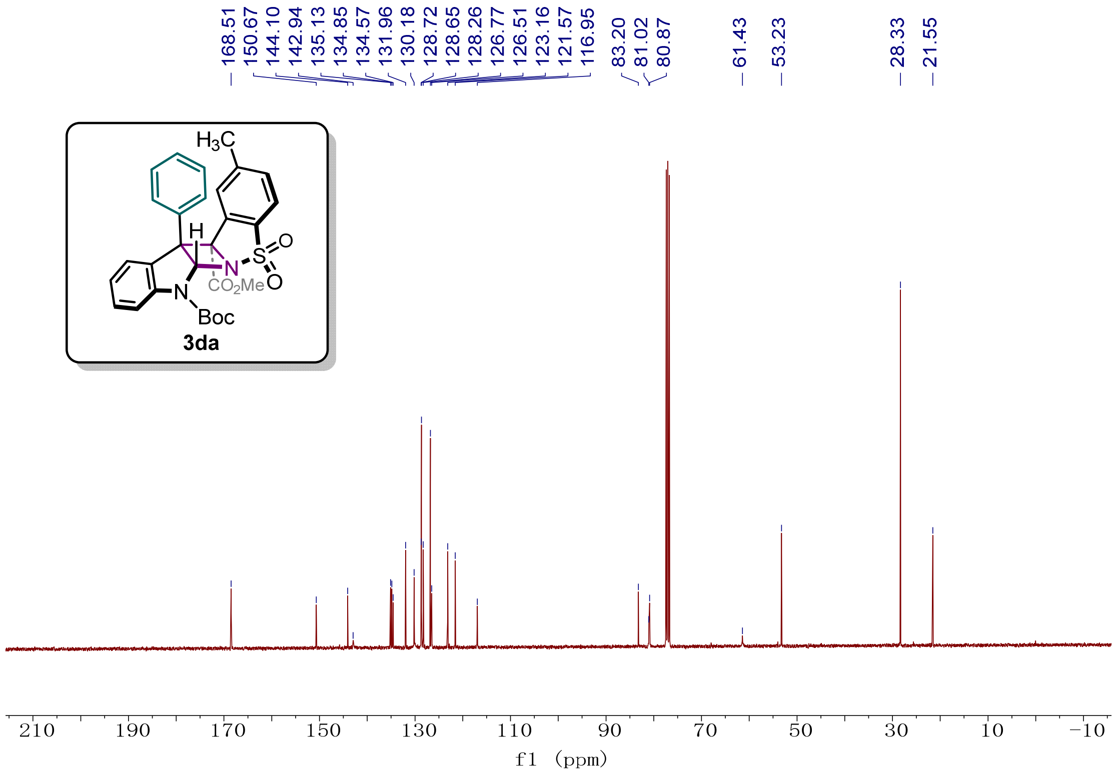

Supplementary Fig. 23. ^{13}C NMR of compound **3da (101 MHz, CDCl$_3$)**

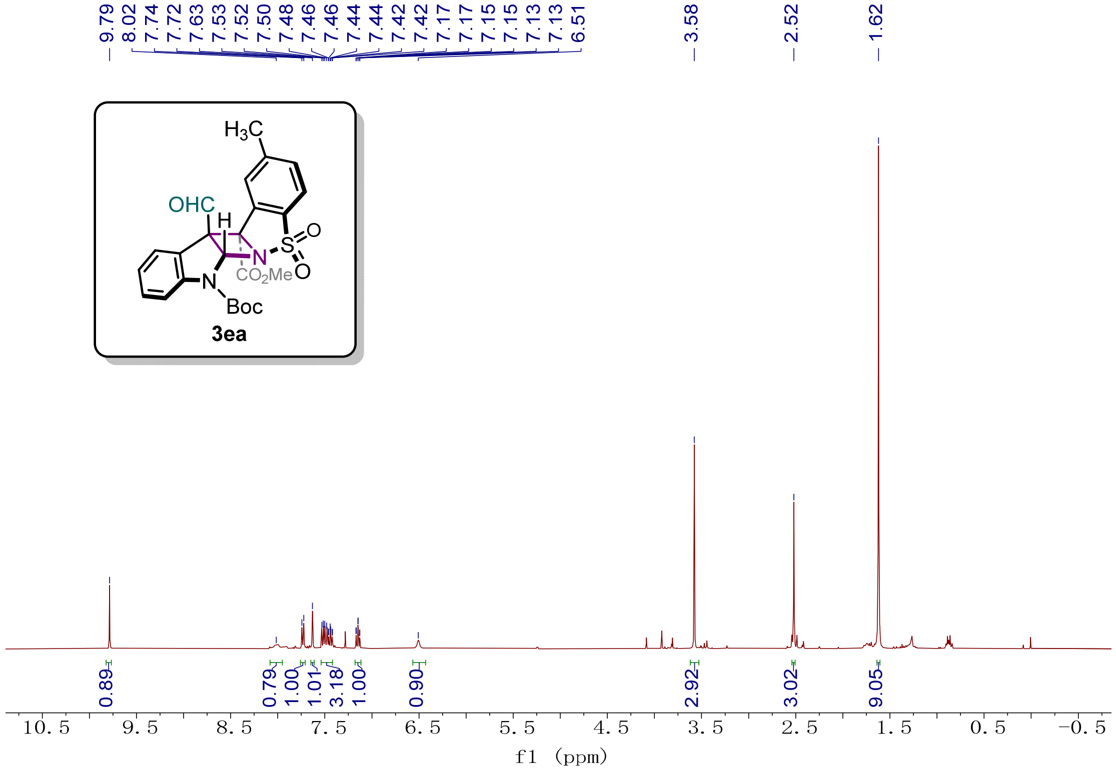

Supplementary Fig. 24. ^{1}H NMR of compound **3ea (400 MHz, CDCl$_3$)**

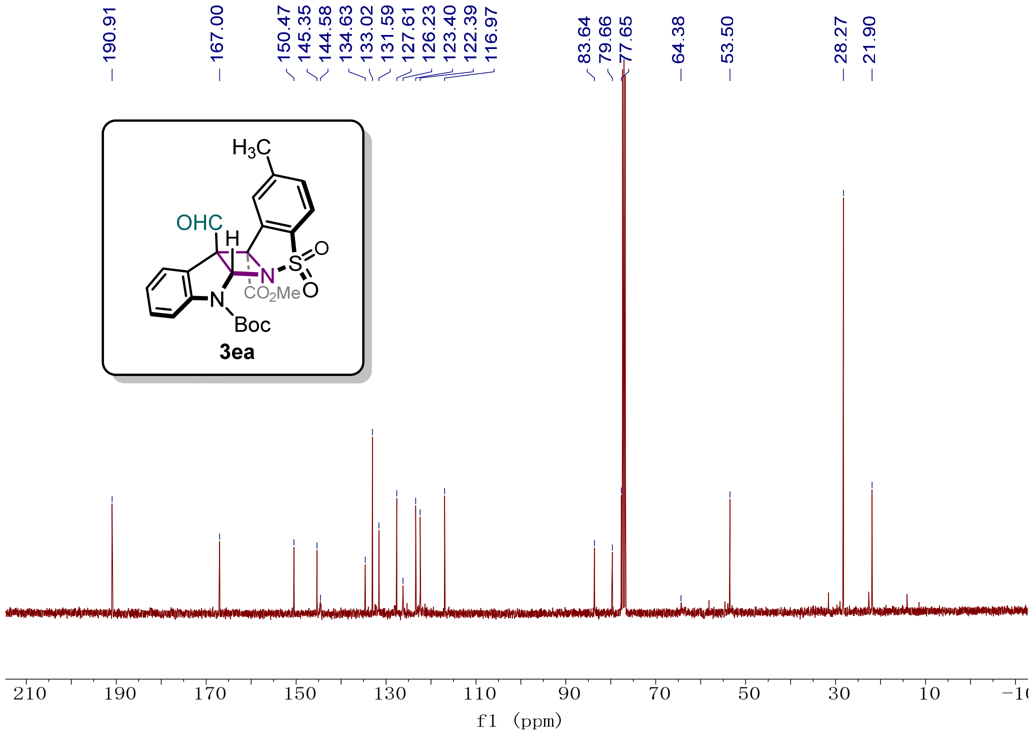

Supplementary Fig. 25. ^{13}C NMR of compound **3ea (101 MHz, CDCl$_3$)**

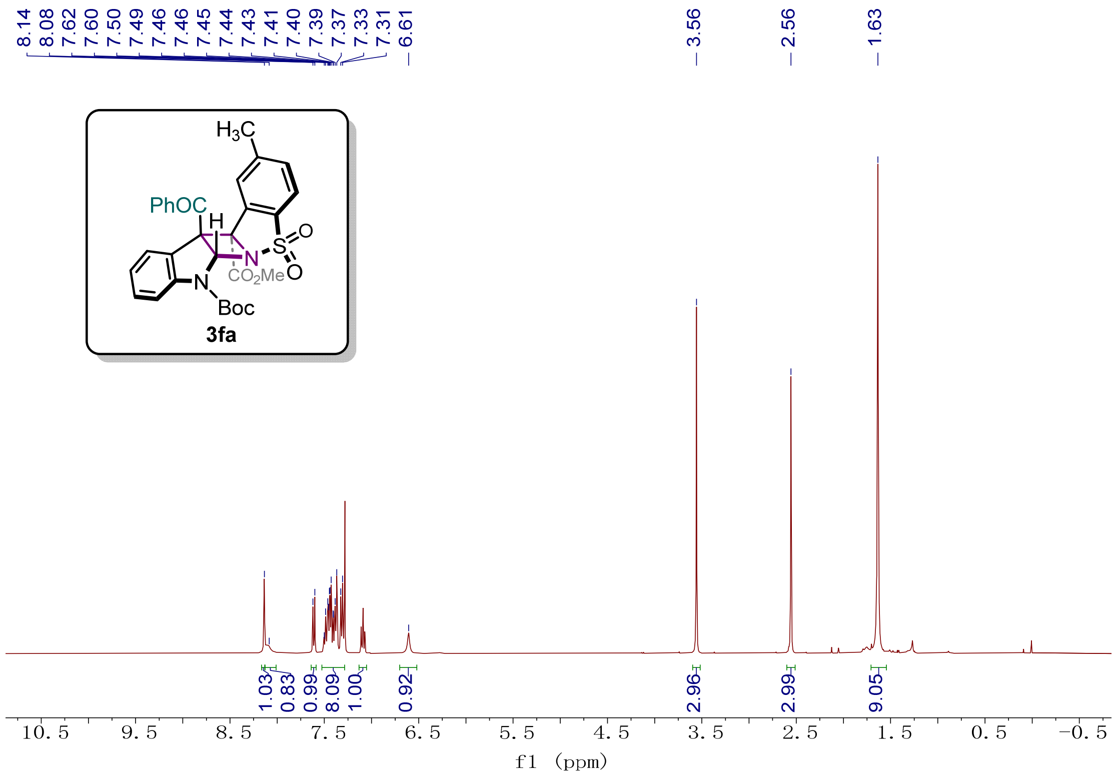

Supplementary Fig. 26. ^{1}H NMR of compound **3fa (400 MHz, CDCl$_3$)**

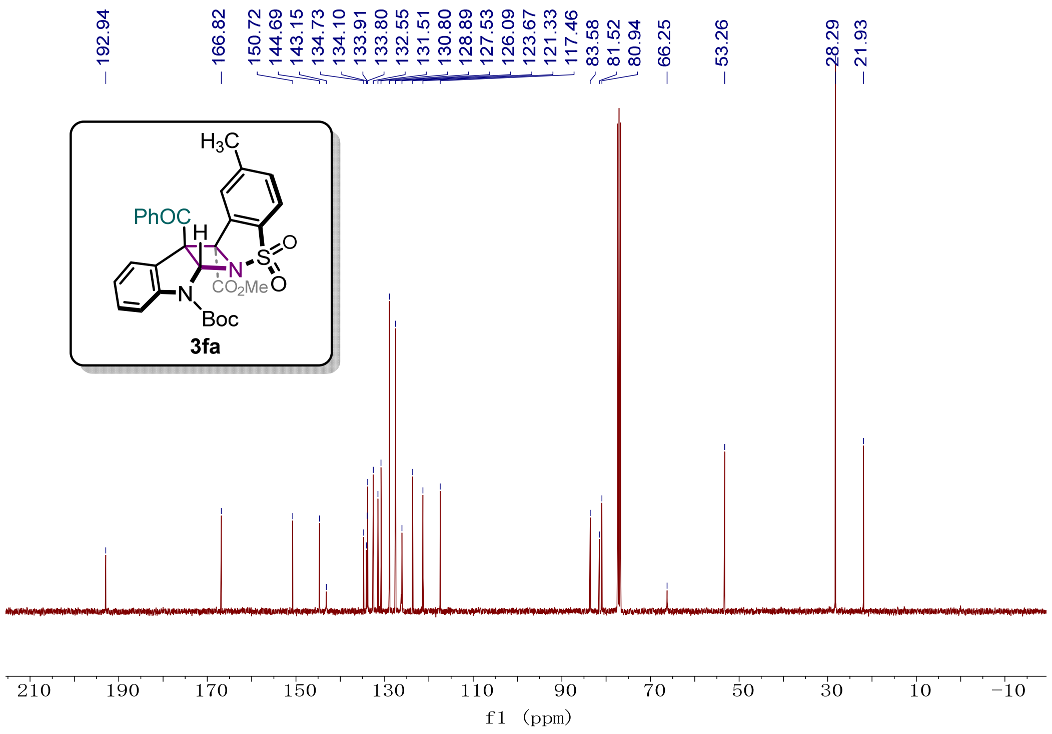

Supplementary Fig. 27. ^{13}C NMR of compound **3fa (101 MHz, CDCl$_3$)**

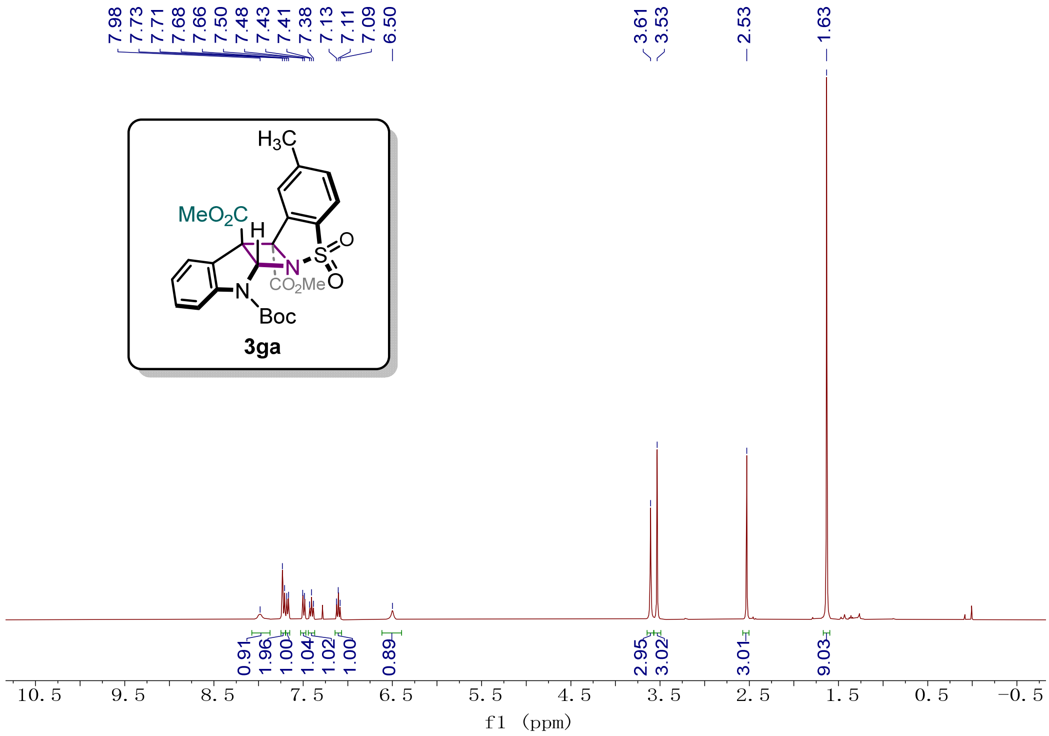

Supplementary Fig. 28. ^{1}H NMR of compound **3ga (400 MHz, $CDCl_3$)**

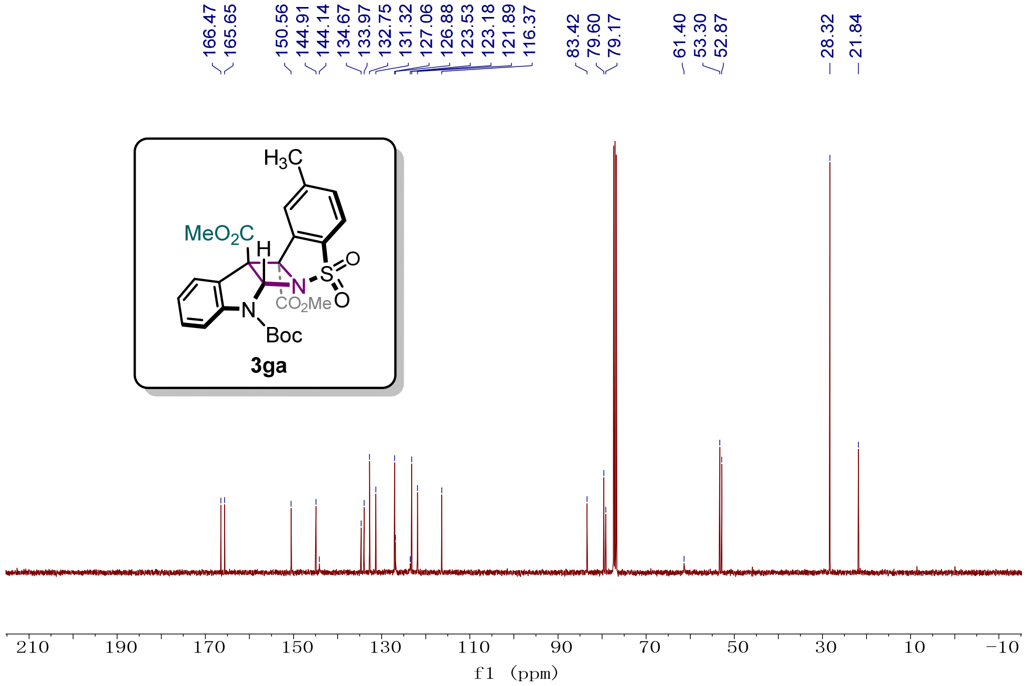

Supplementary Fig. 29. ^{13}C NMR of compound **3ga (101 MHz, $CDCl_3$)**

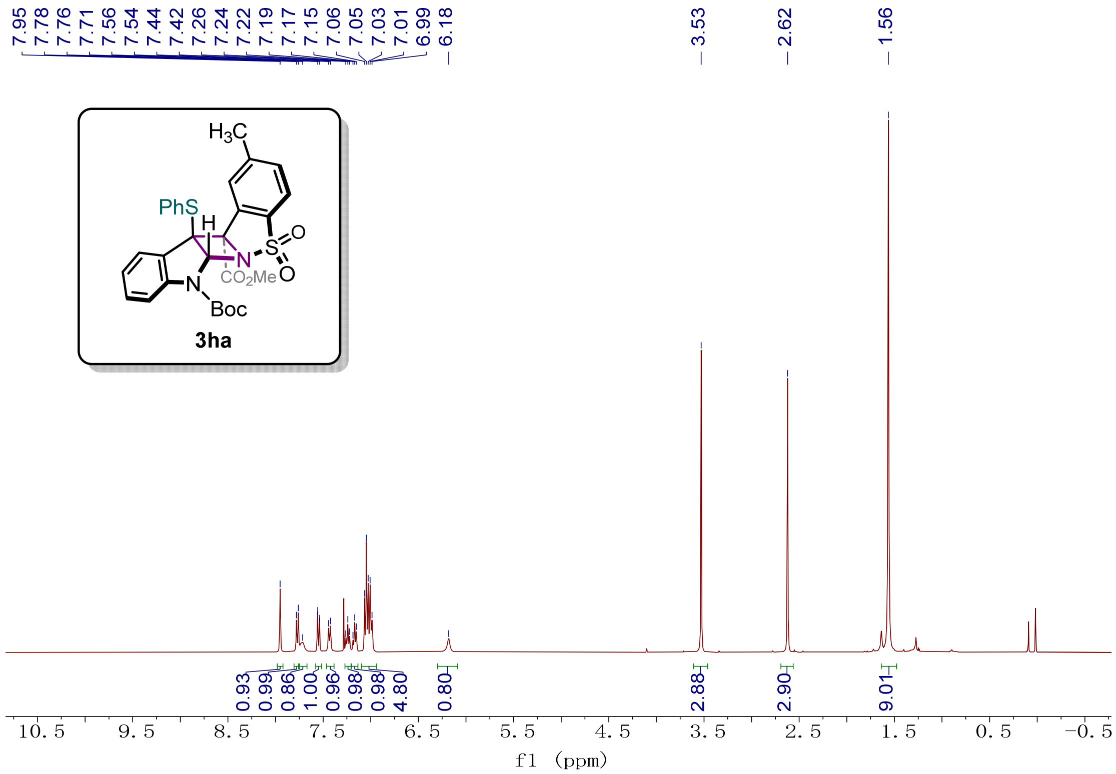

Supplementary Fig. 30. ^{1}H NMR of compound **3ha (400 MHz, CDCl₃)**

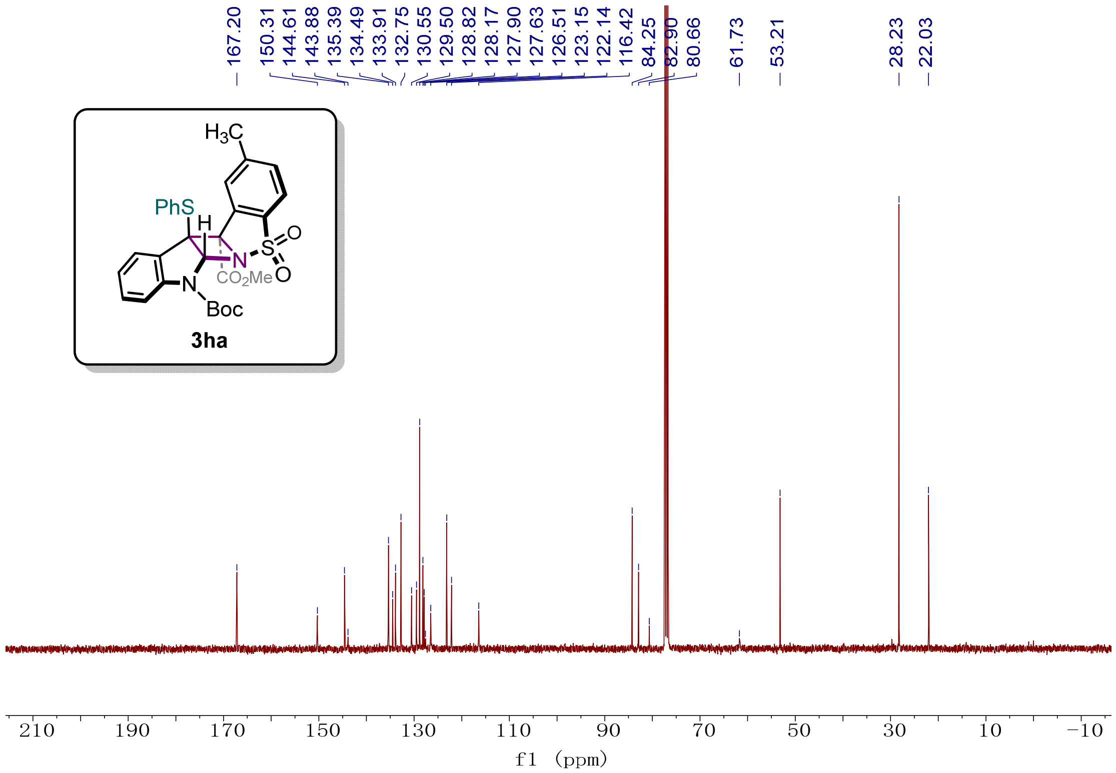

Supplementary Fig. 31. ^{13}C NMR of compound **3ha (101 MHz, CDCl₃)**

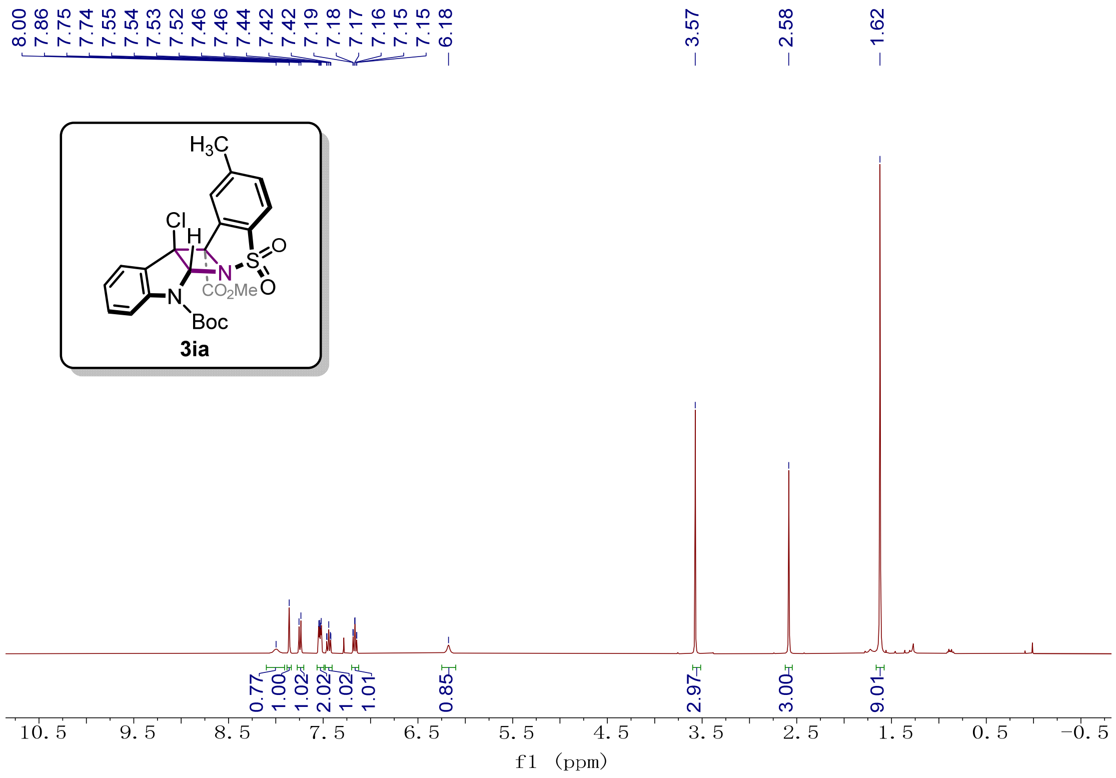

Supplementary Fig. 32. ^{1}H NMR of compound **3ia (400 MHz, $CDCl_3$)**

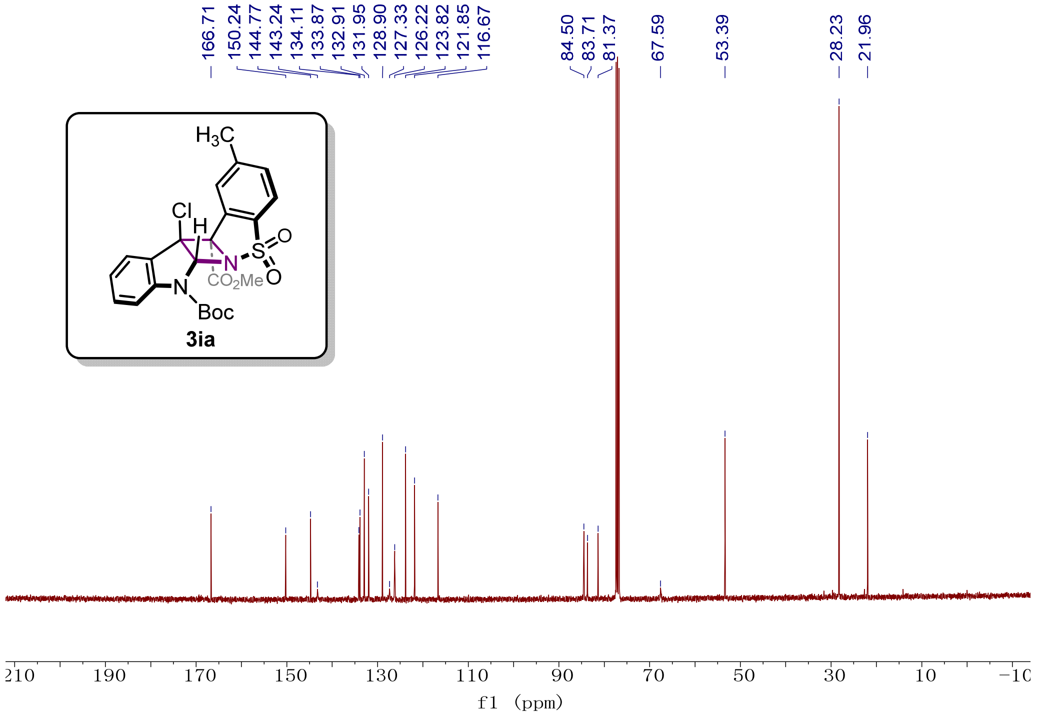

Supplementary Fig. 33. ^{13}C NMR of compound **3ia (101 MHz, $CDCl_3$)**

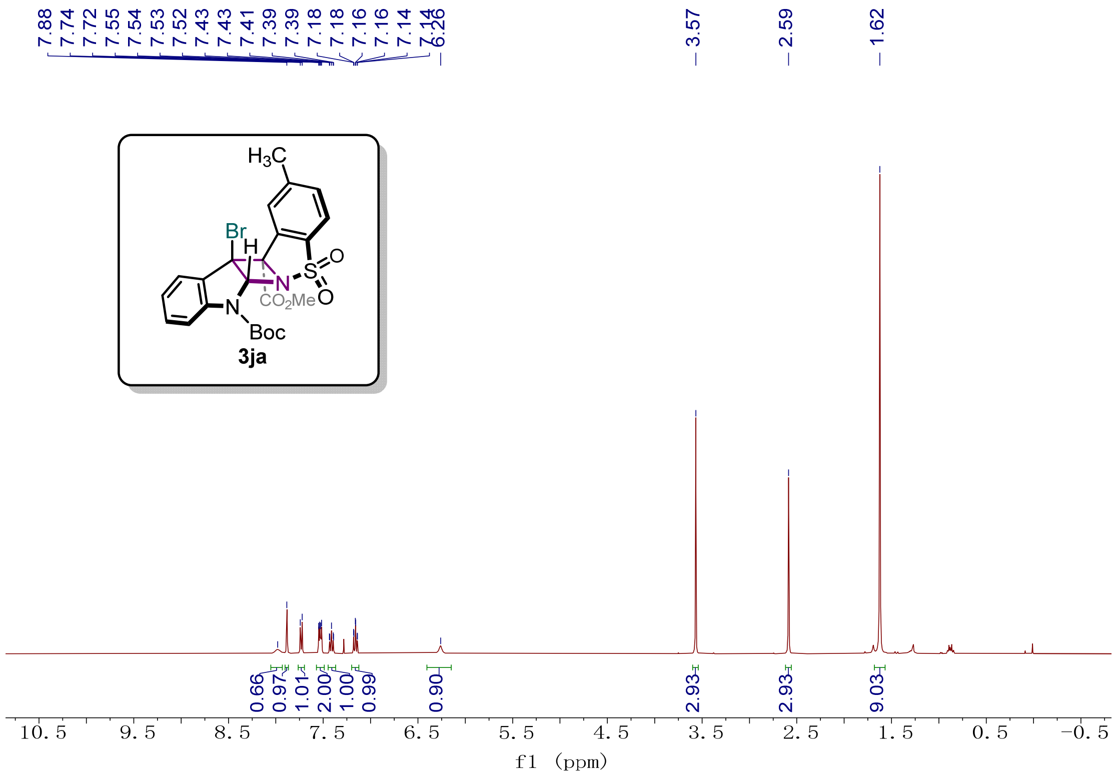

Supplementary Fig. 34. ^{1}H NMR of compound **3ja (400 MHz, $CDCl_3$)**

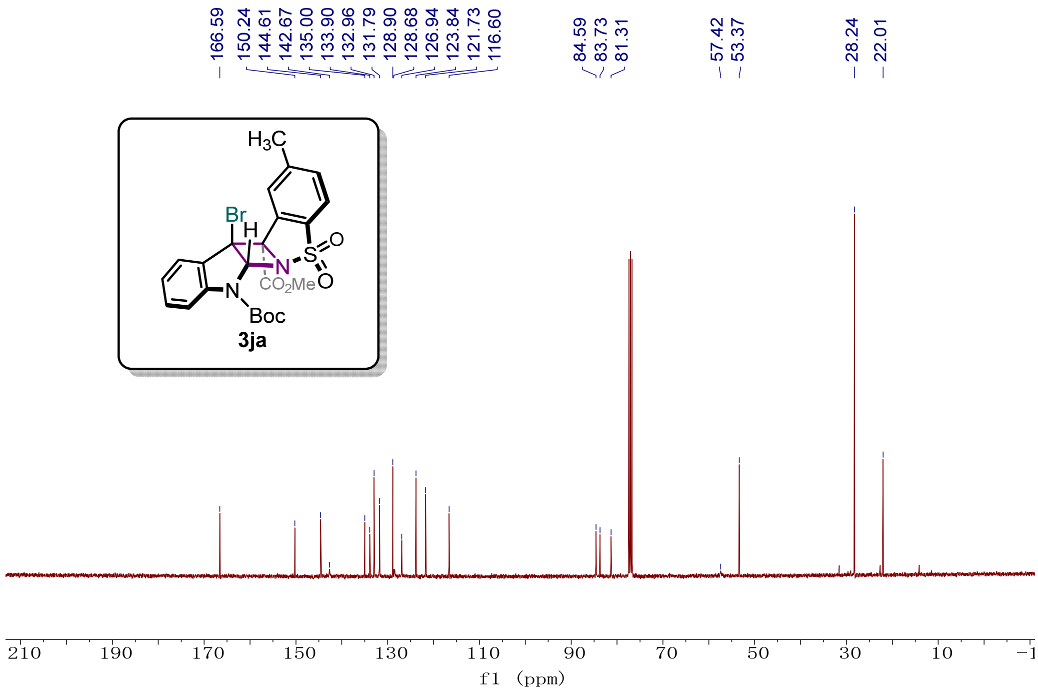

Supplementary Fig. 35. ^{13}C NMR of compound **3ja (101 MHz, $CDCl_3$)**

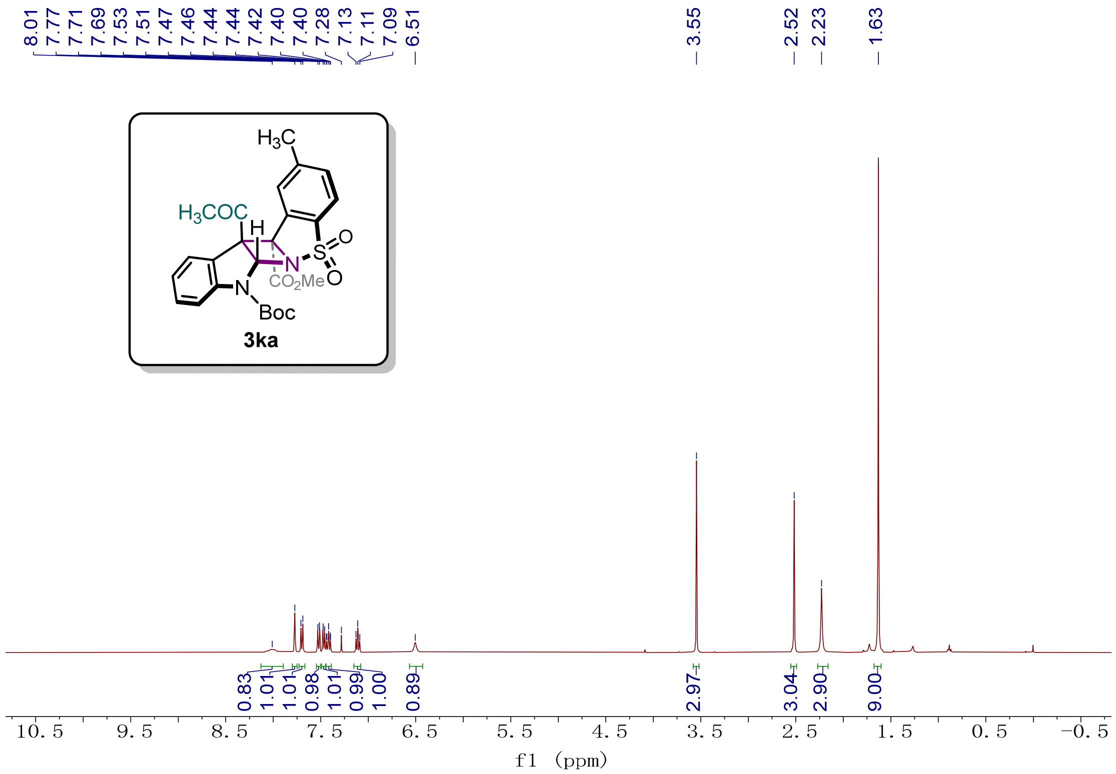

Supplementary Fig. 36. ^{1}H NMR of compound **3ka (400 MHz, CDCl₃)**

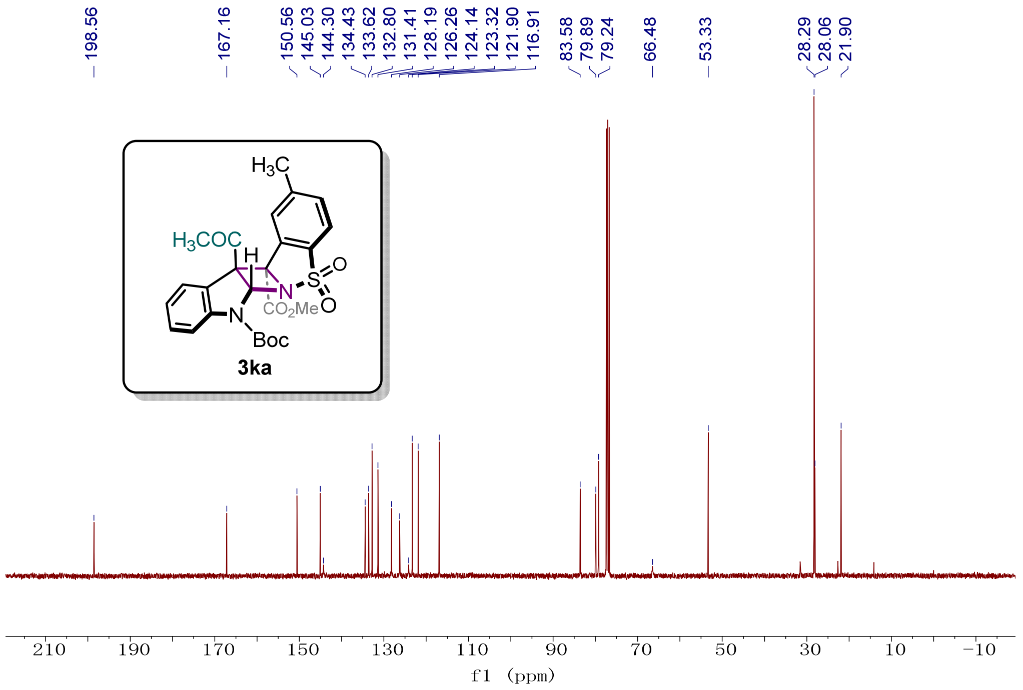

Supplementary Fig. 37. ^{13}C NMR of compound **3ka (101 MHz, CDCl₃)**

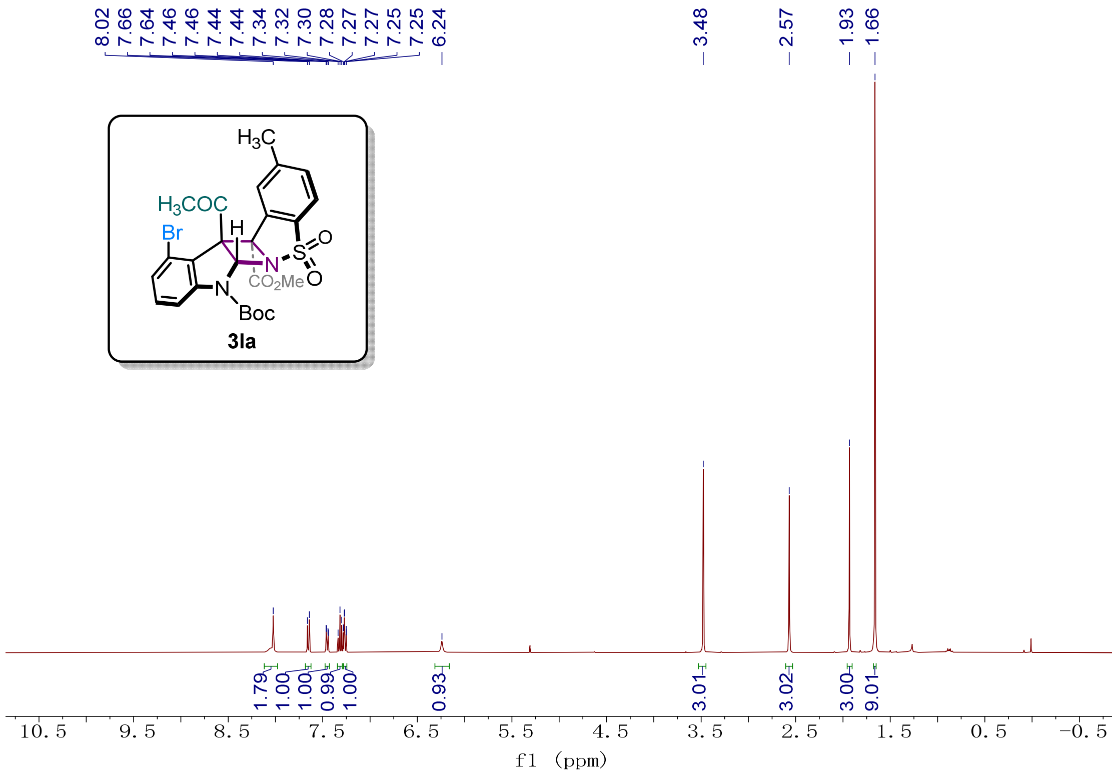

Supplementary Fig. 38. ^{1}H NMR of compound **3la (400 MHz, CDCl$_3$)**

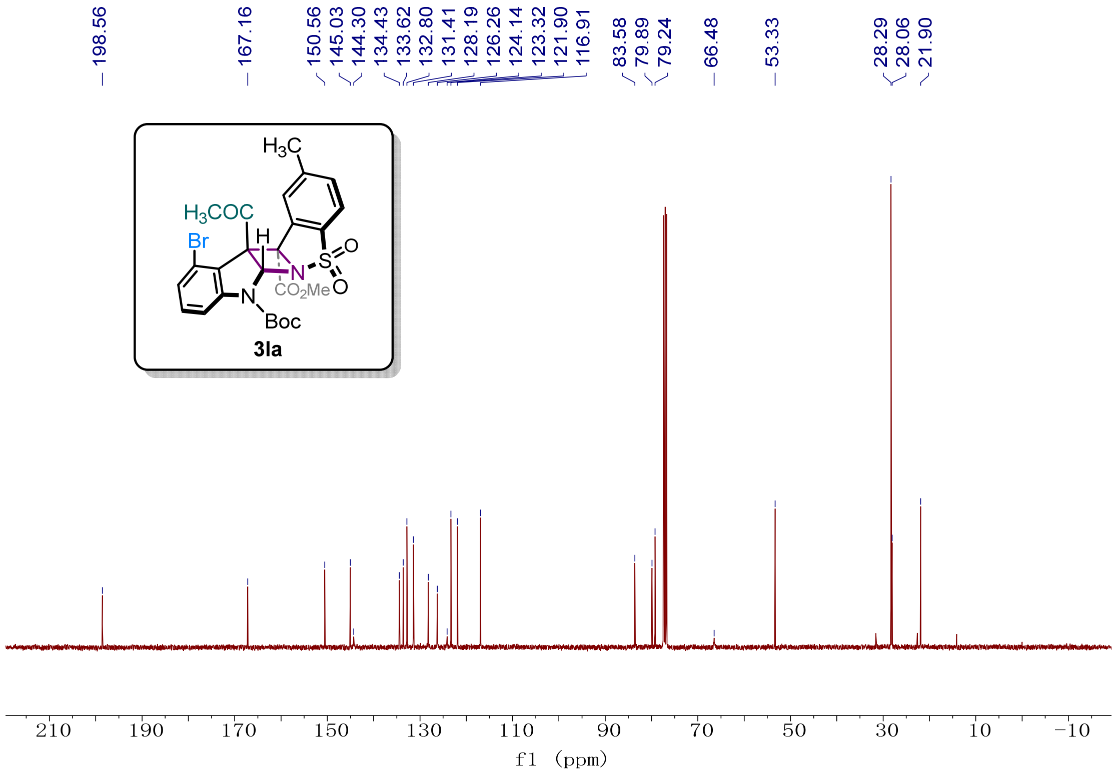

Supplementary Fig. 39. ^{13}C NMR of compound **3la (101 MHz, CDCl$_3$)**

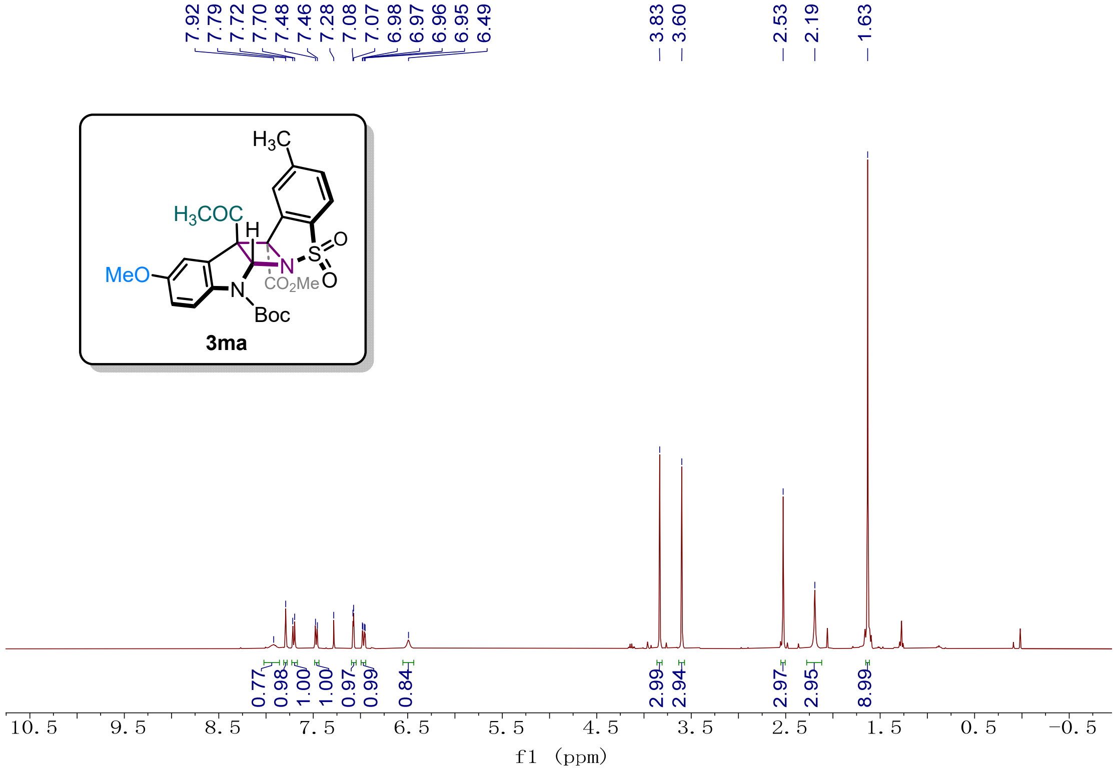

Supplementary Fig. 40. ^{1}H NMR of compound **3ma (400 MHz, CDCl$_3$)**

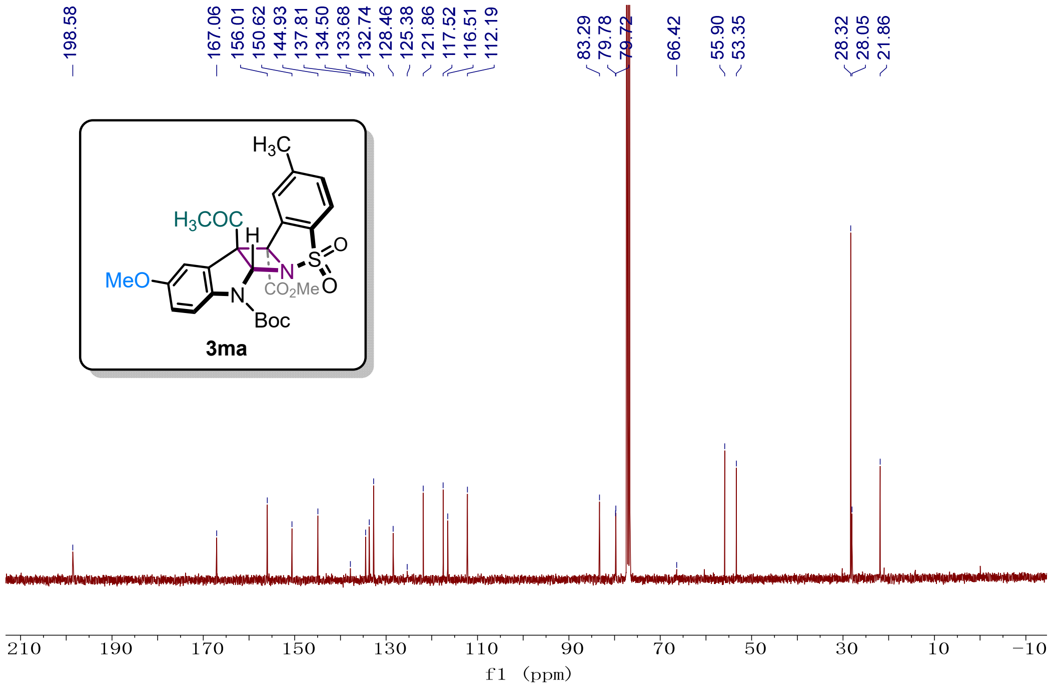

Supplementary Fig. 41. ^{13}C NMR of compound **3ma (101 MHz, CDCl$_3$)**

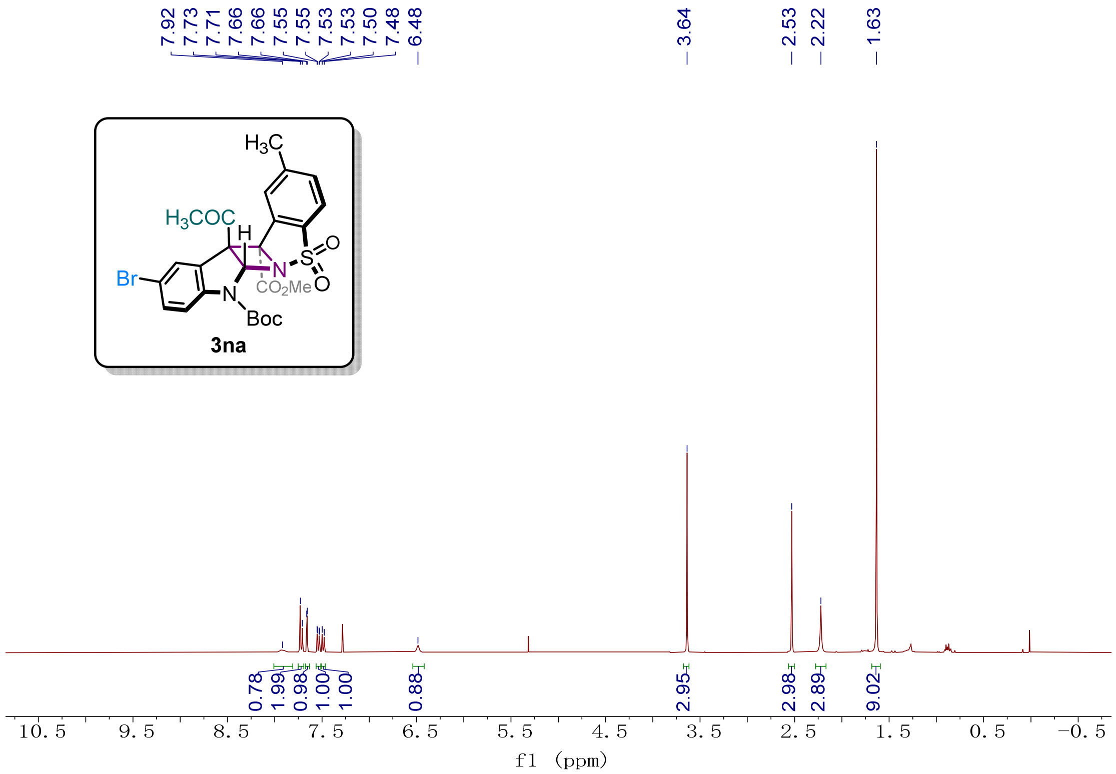

Supplementary Fig. 42. ^{1}H NMR of compound **3na (400 MHz, $CDCl_3$)**

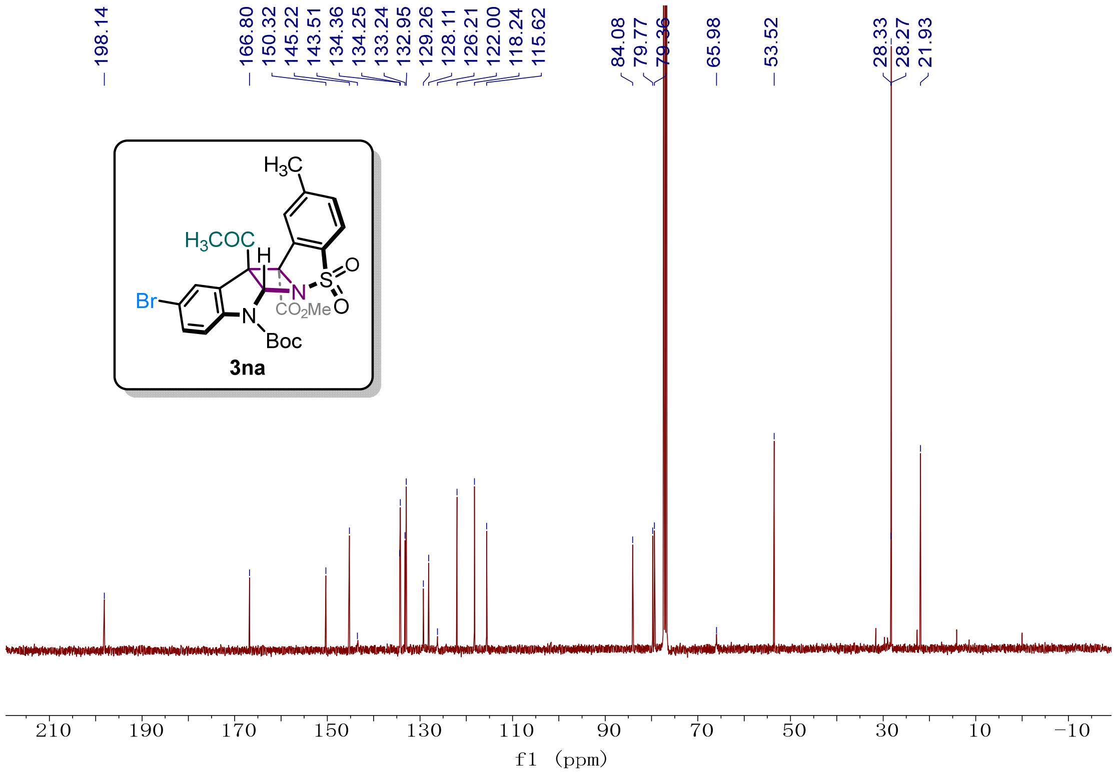

Supplementary Fig. 41. ^{13}C NMR of compound **3na (101 MHz, $CDCl_3$)**

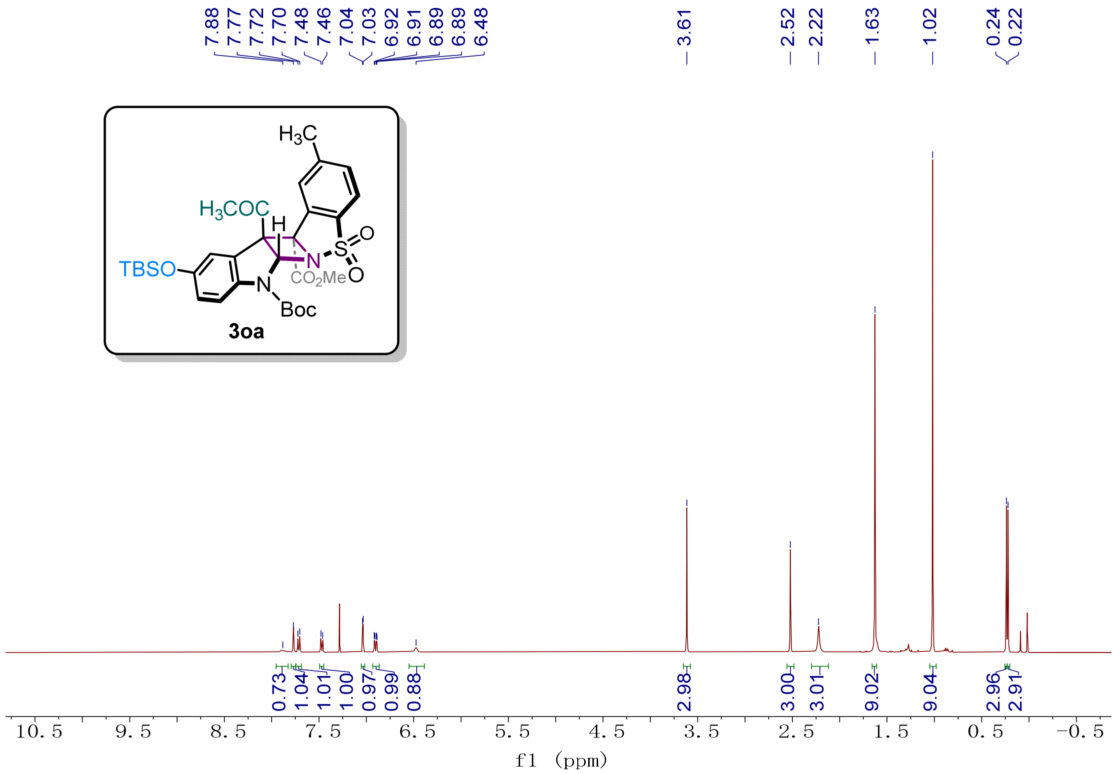

Supplementary Fig. 42. ^{1}H NMR of compound **3oa (400 MHz, $CDCl_3$)**

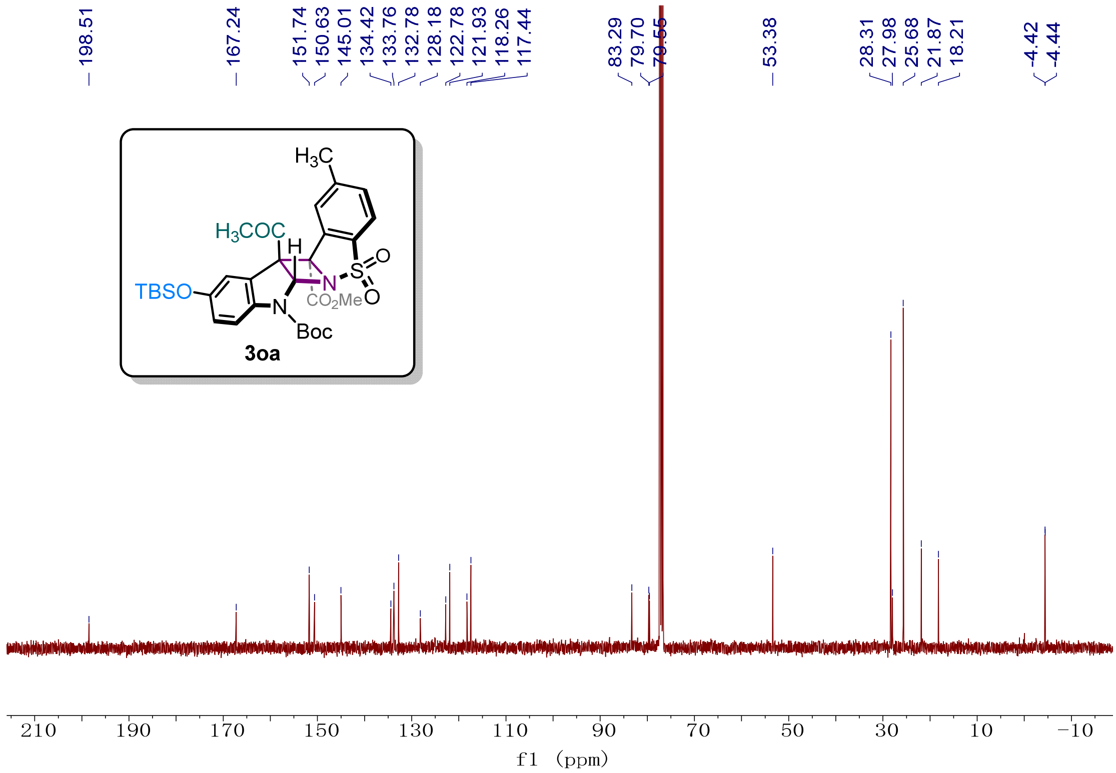

Supplementary Fig. 43. ^{13}C NMR of compound **3oa (101 MHz, $CDCl_3$)**

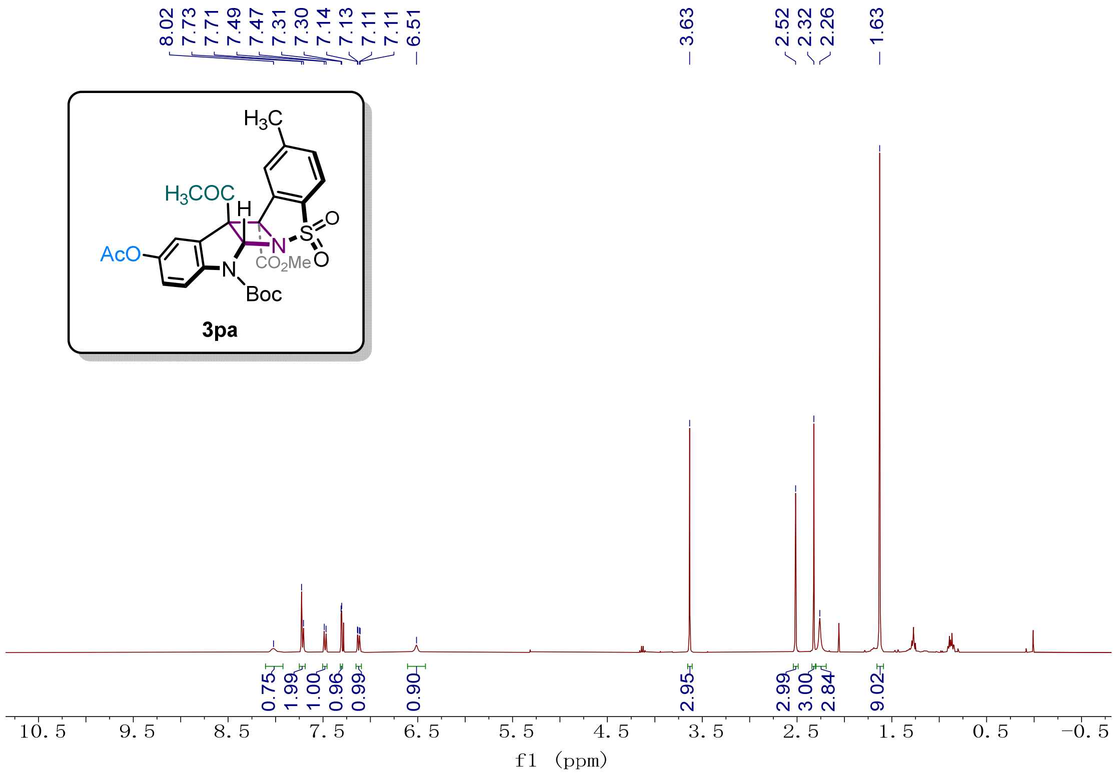

Supplementary Fig. 44. ^{1}H NMR of compound **3pa (400 MHz, CDCl$_3$)**

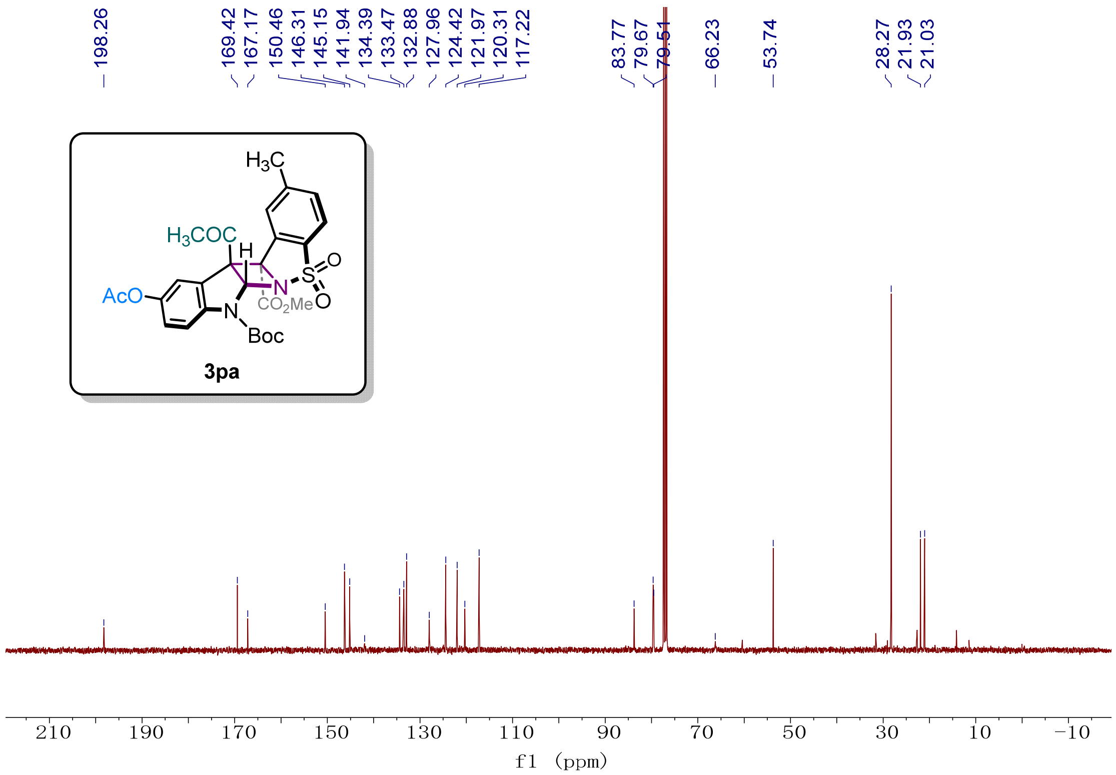

Supplementary Fig. 45. ^{13}C NMR of compound **3pa (101 MHz, CDCl$_3$)**

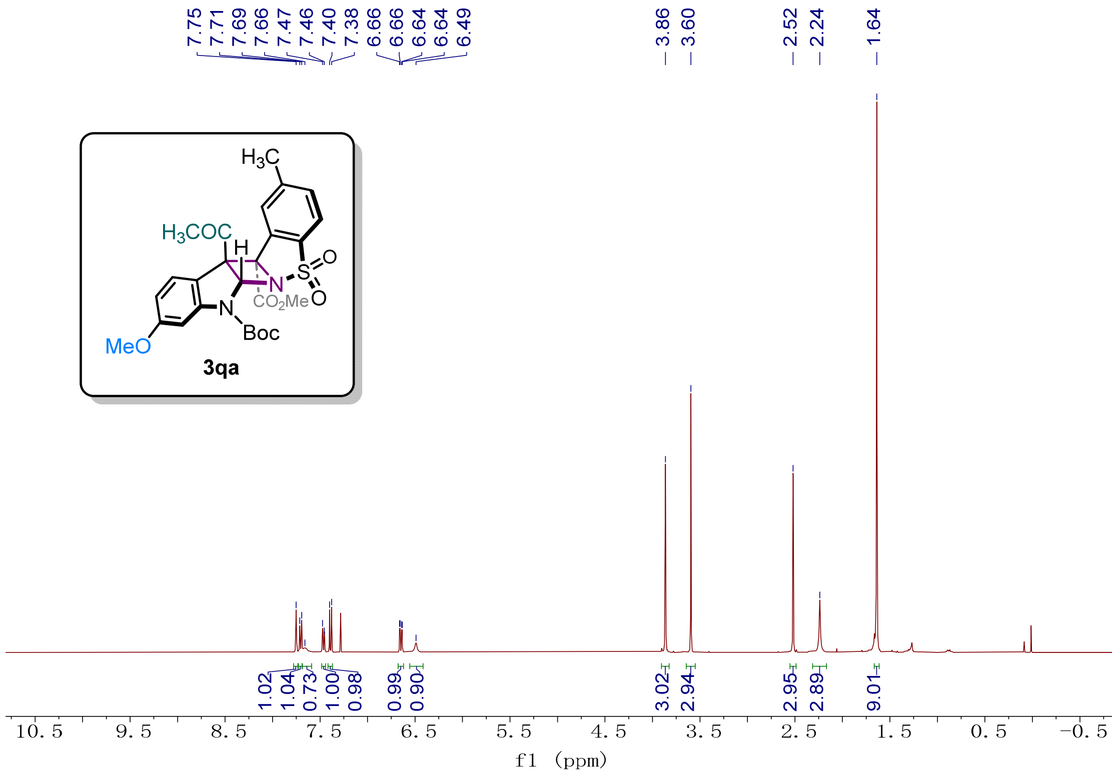

Supplementary Fig. 46. ^{1}H NMR of compound **3qa (400 MHz, $CDCl_3$)**

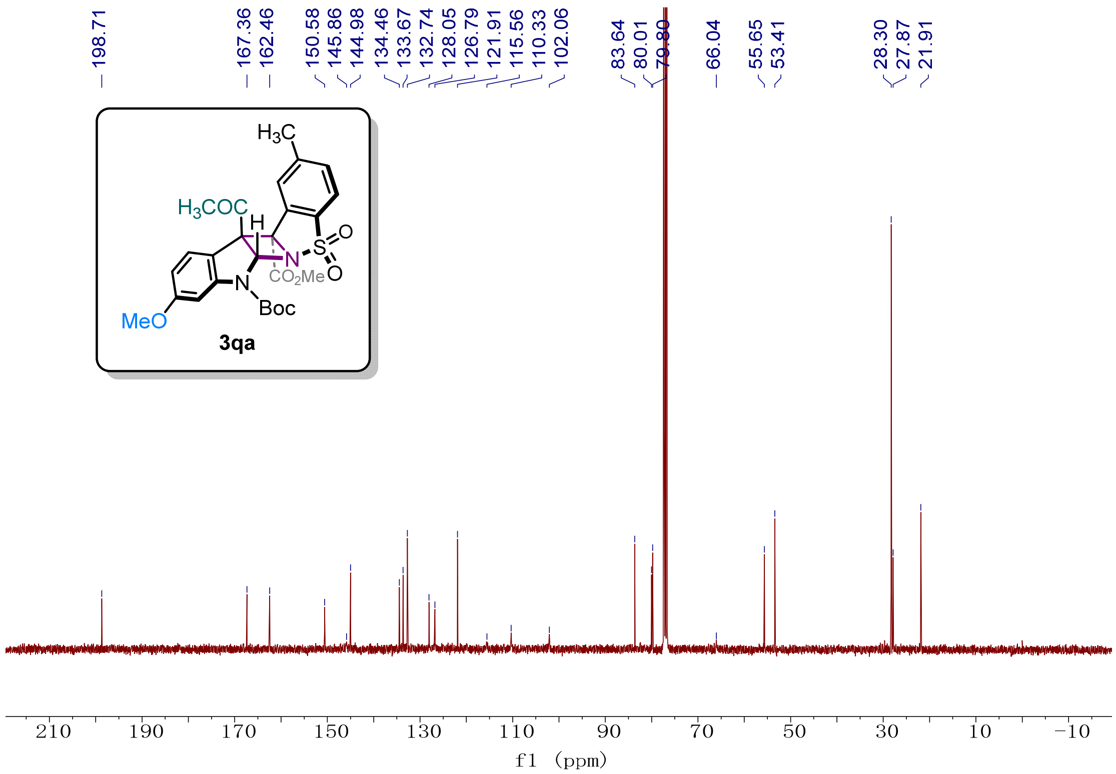

Supplementary Fig. 47. ^{13}C NMR of compound **3qa (101 MHz, $CDCl_3$)**

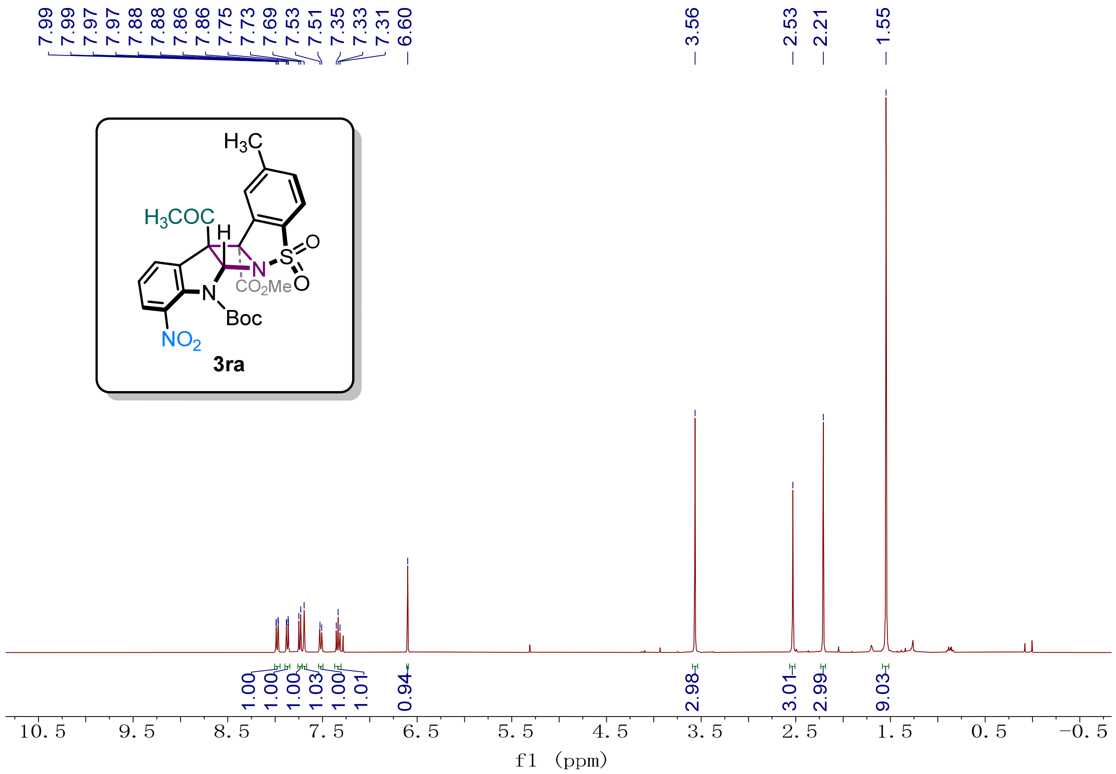

Supplementary Fig. 48. ^{1}H NMR of compound **3ra (400 MHz, CDCl$_3$)**

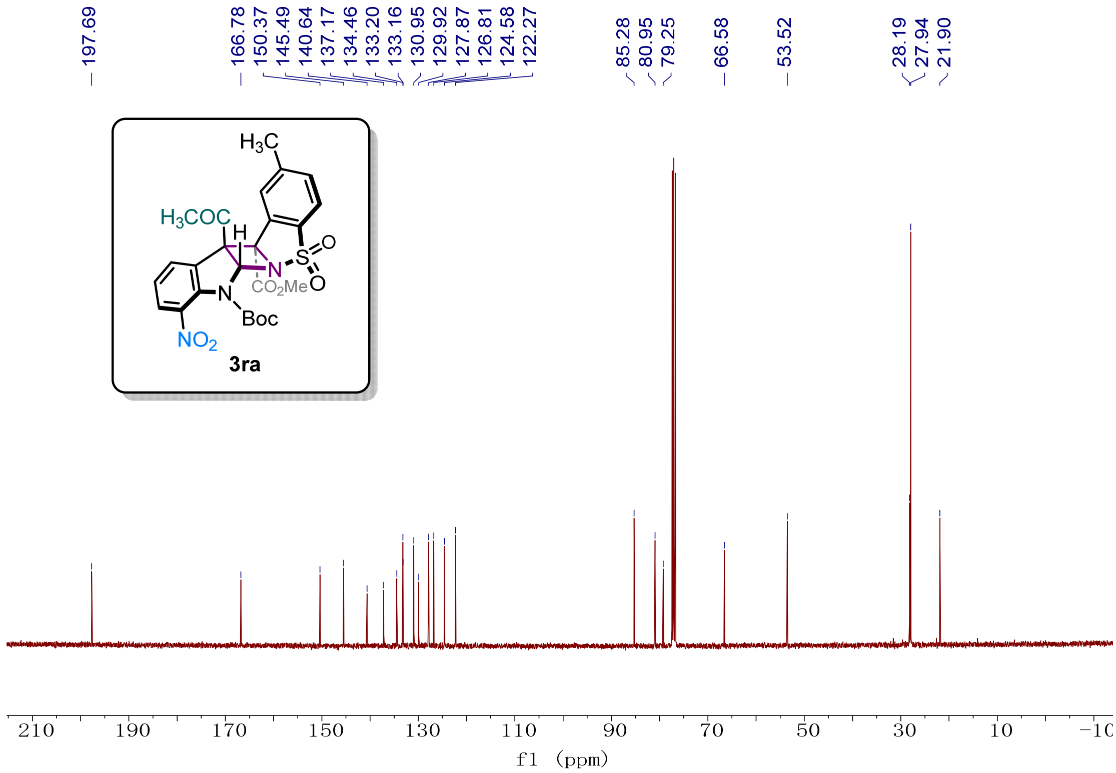

Supplementary Fig. 49. ^{13}C NMR of compound **3ra (101 MHz, CDCl$_3$)**

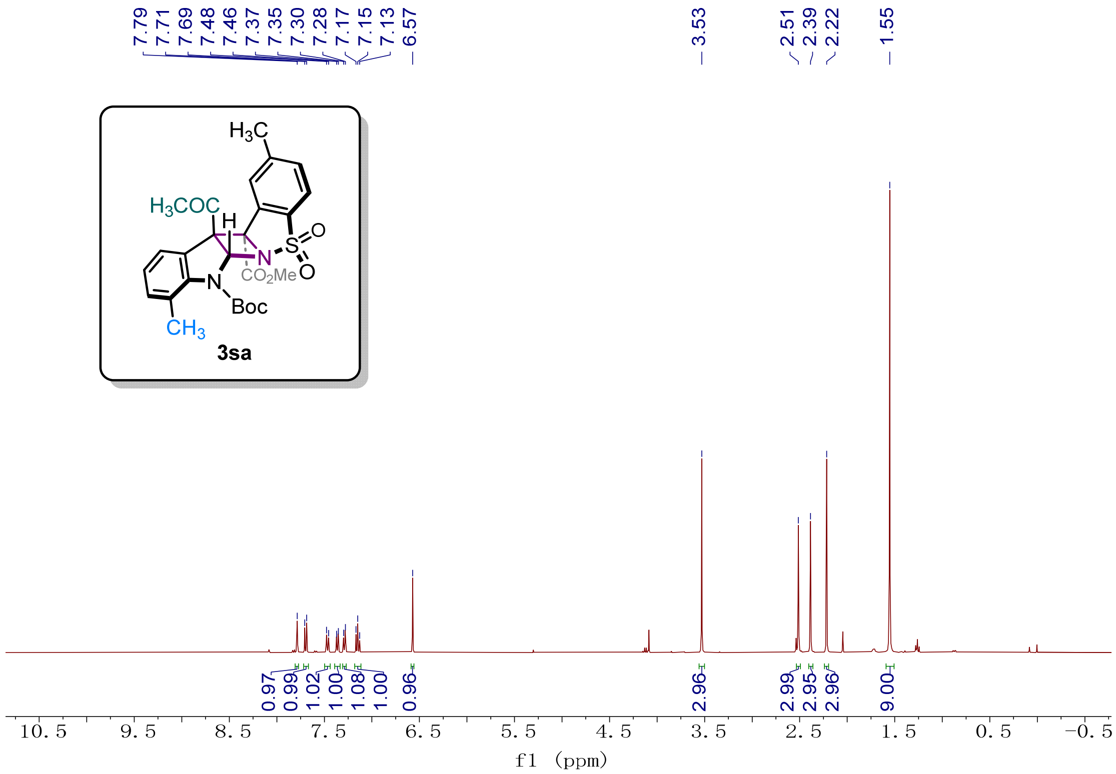

Supplementary Fig. 50. ^{1}H NMR of compound **3sa (400 MHz, CDCl₃)**

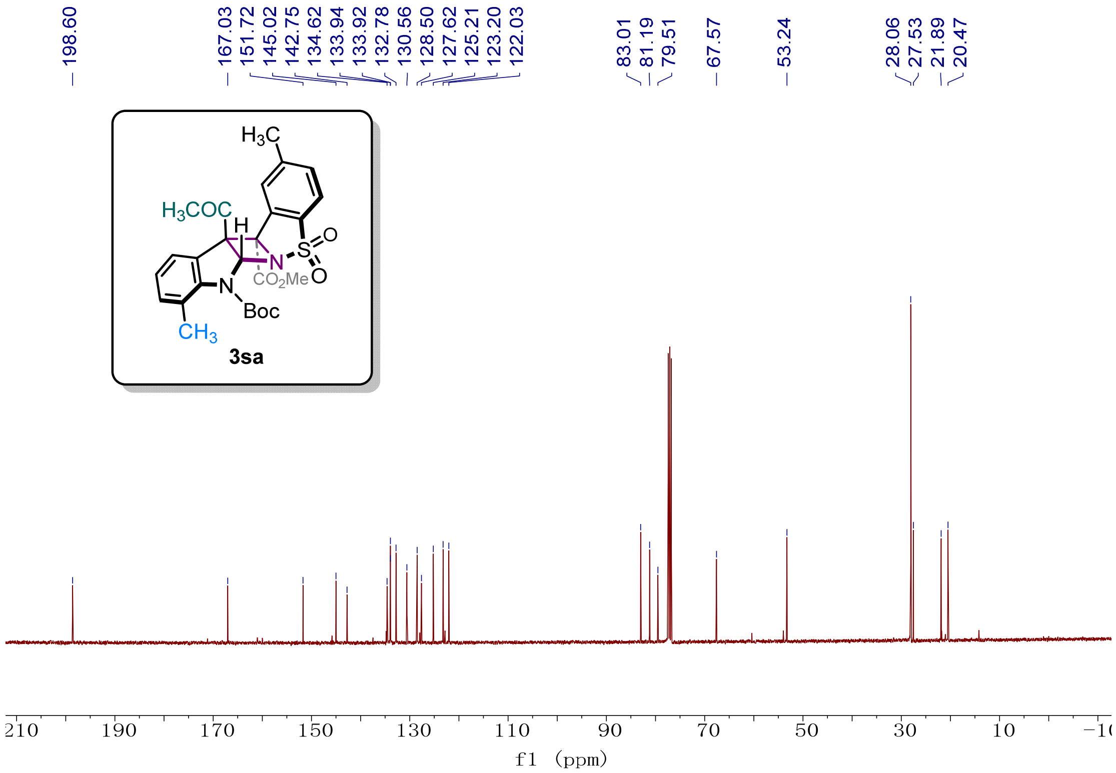

Supplementary Fig. 51. ^{13}C NMR of compound **3sa (101 MHz, CDCl₃)**

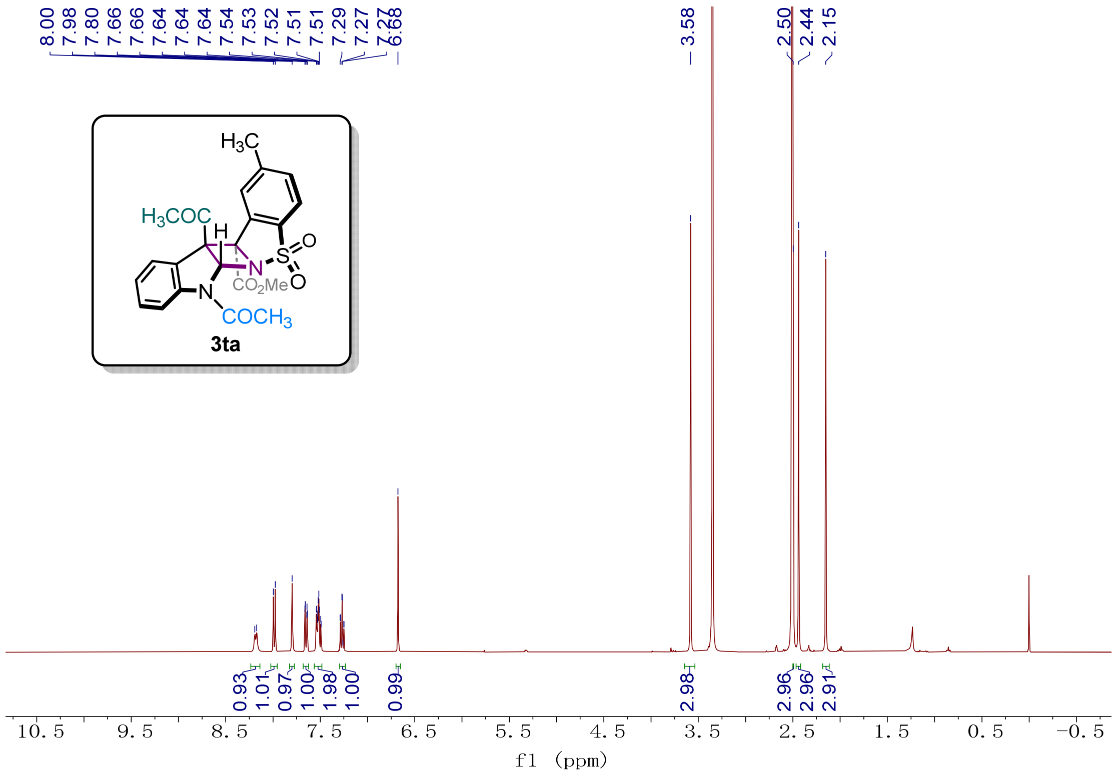

Supplementary Fig. 52. ^{1}H NMR of compound **3ta (400 MHz, DMSO-*d6*)**

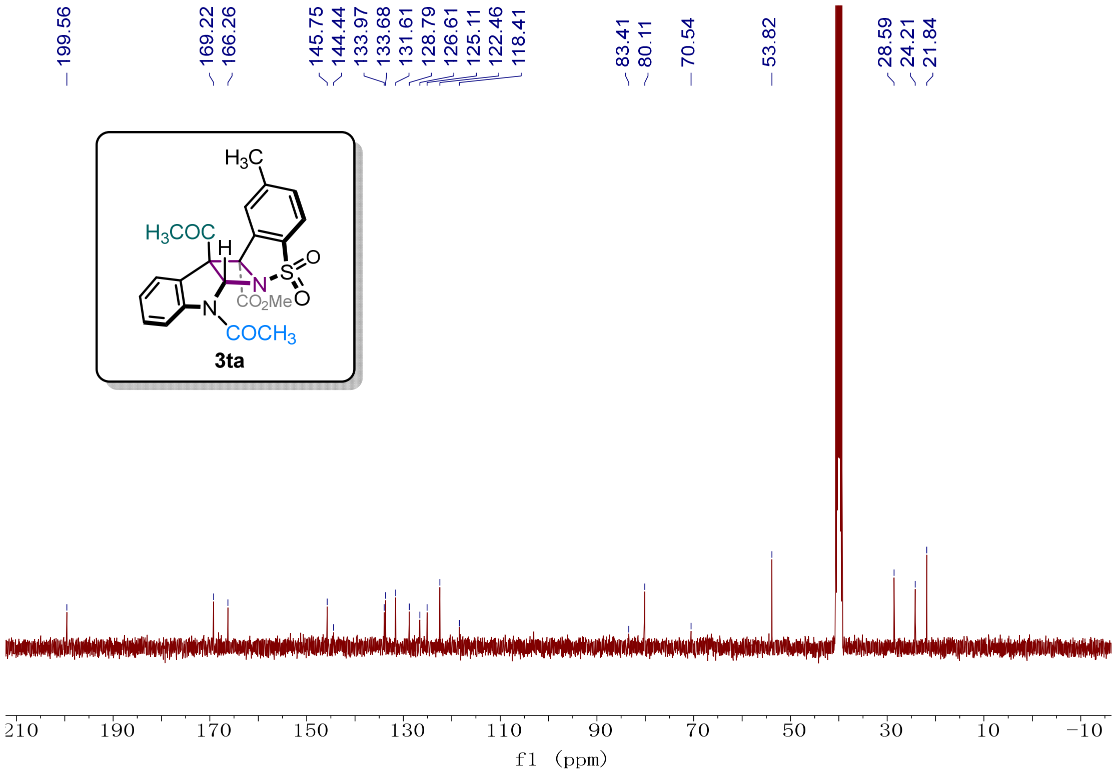

Supplementary Fig. 53. ^{13}C NMR of compound **3ta (101 MHz, DMSO-*d6*)**

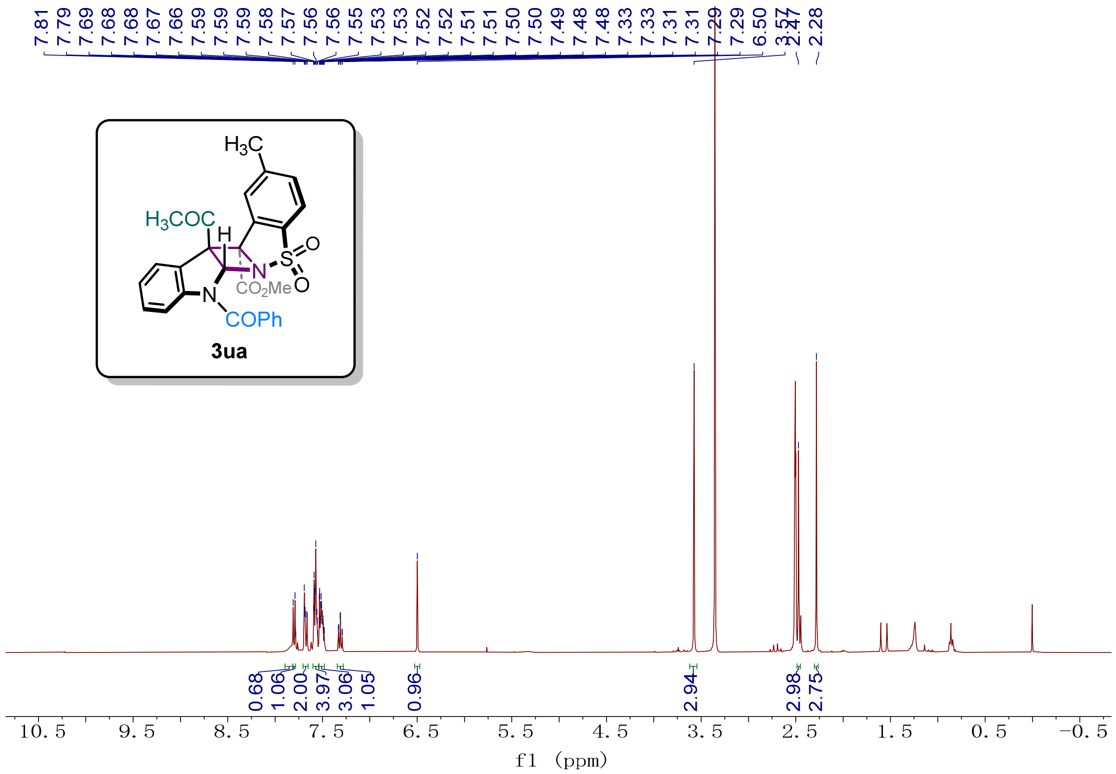

Supplementary Fig. 54. ^{1}H NMR of compound **3ua (400 MHz,DMSO-*d6*)**

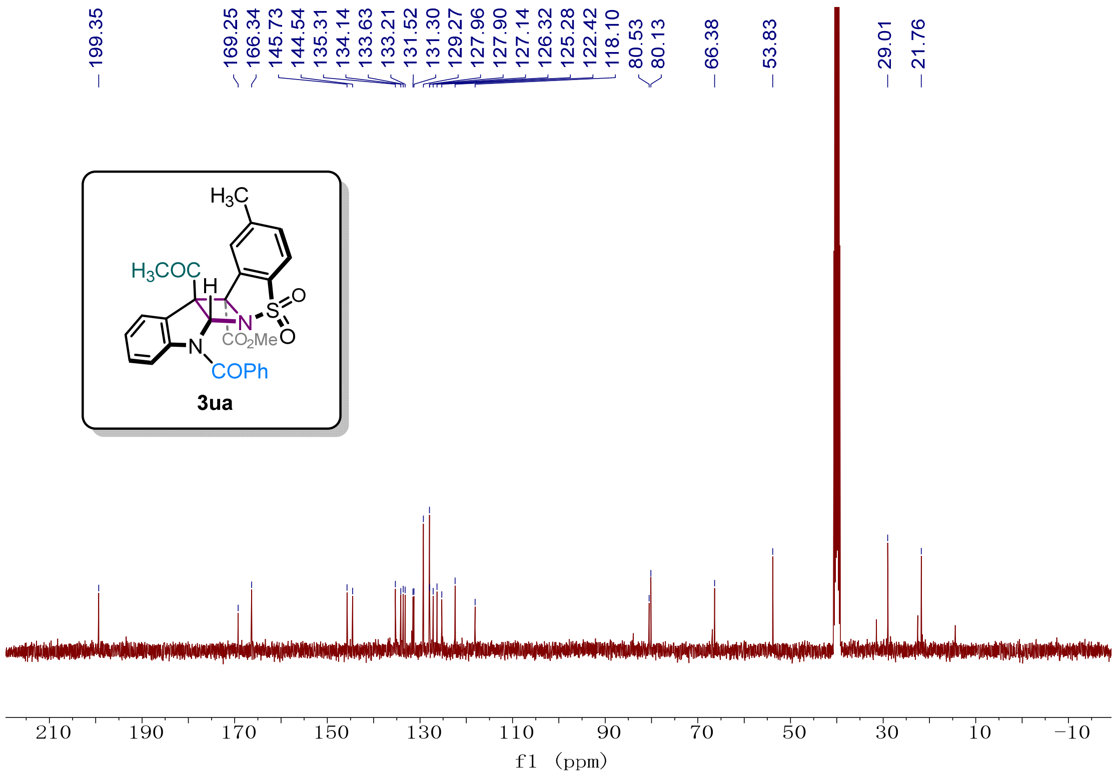

Supplementary Fig. 55. ^{13}C NMR of compound **3ua (101 MHz, DMSO-*d6*)**

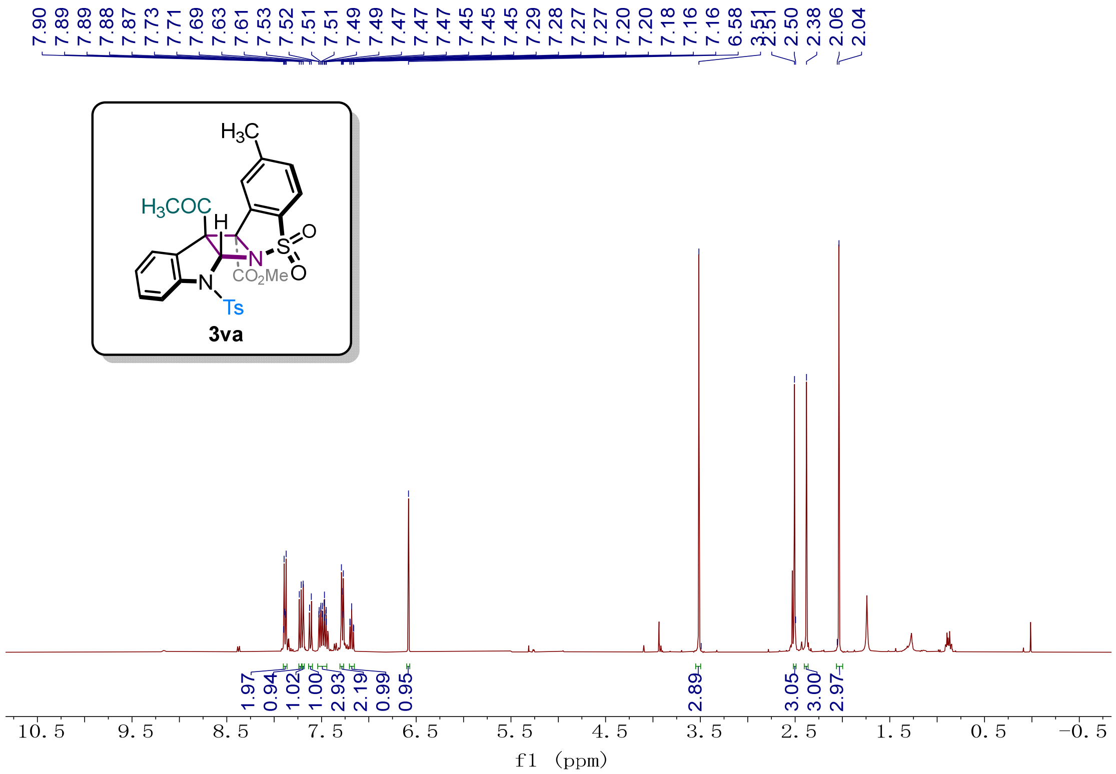

Supplementary Fig. 56. ^{1}H NMR of compound **3va (400 MHz, $CDCl_3$)**

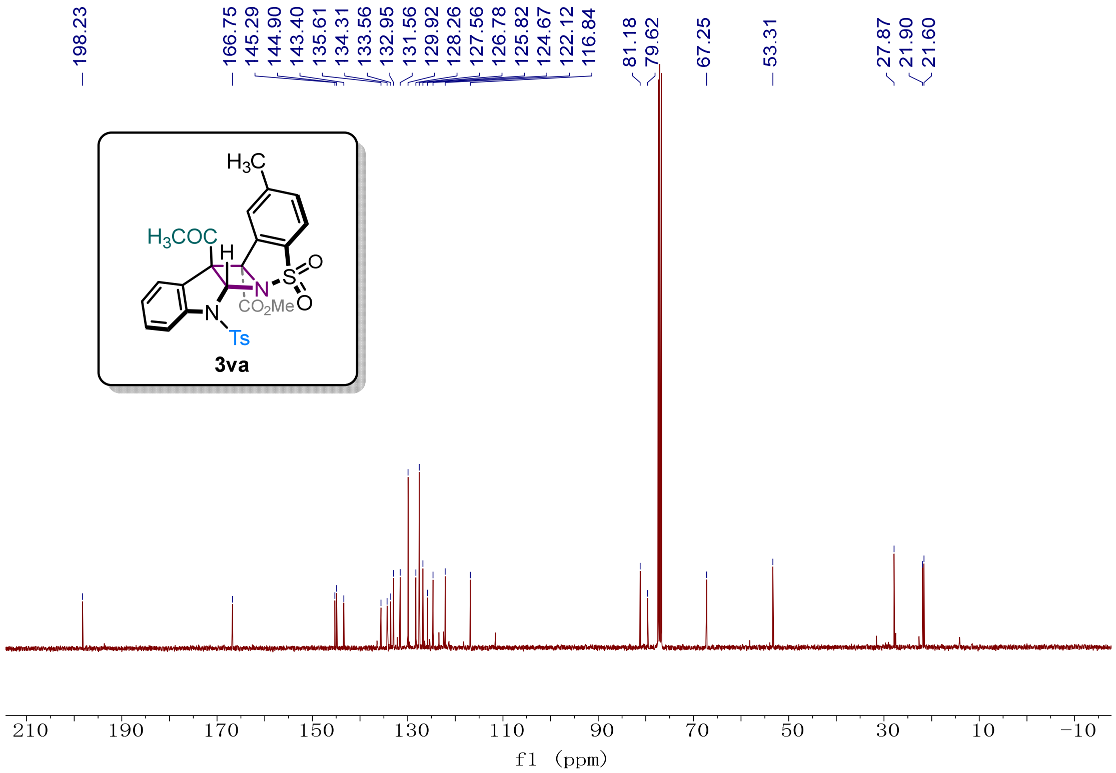

Supplementary Fig. 57. ^{13}C NMR of compound **3va (101 MHz, $CDCl_3$)**

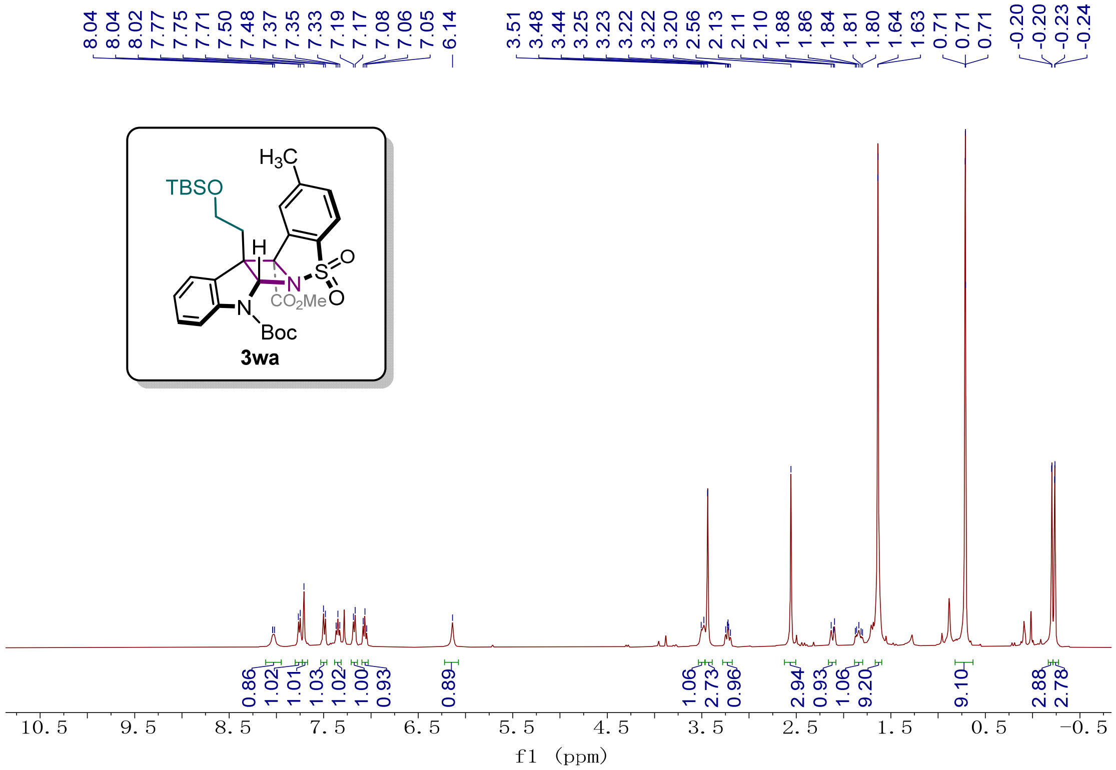

Supplementary Fig. 58. ^{1}H NMR of compound **3wa (400 MHz, CDCl$_3$)**

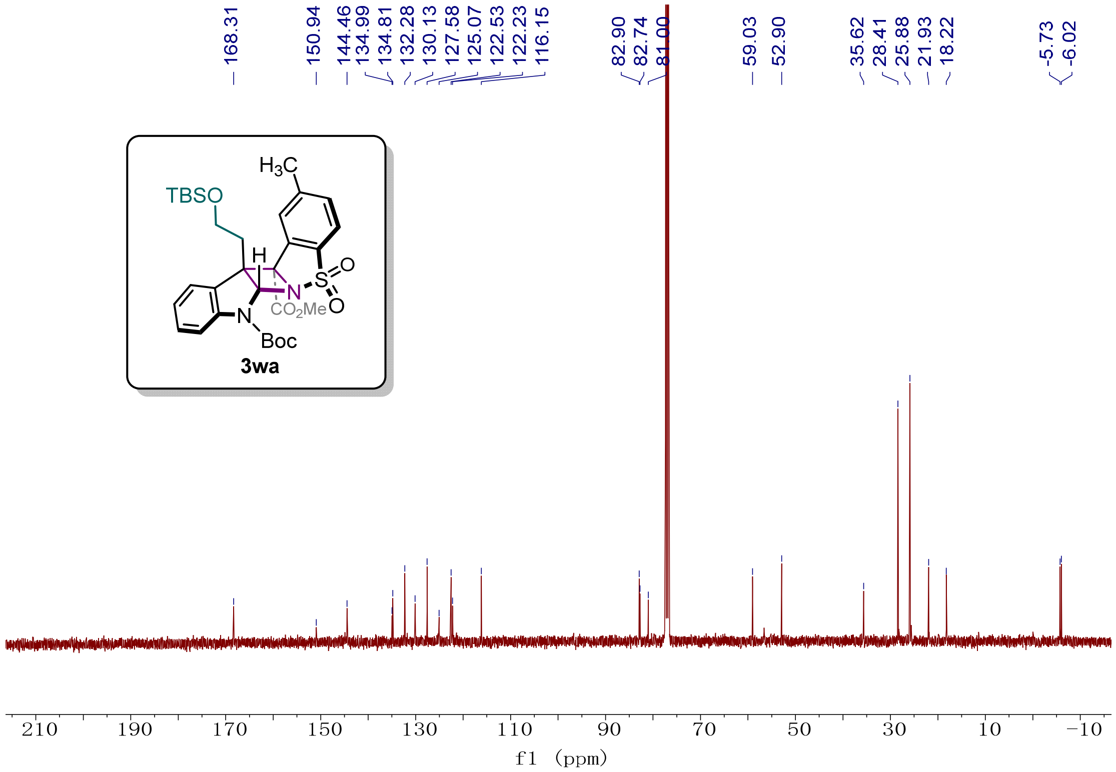

Supplementary Fig. 59. ^{13}C NMR of compound **3wa (101 MHz, CDCl$_3$)**

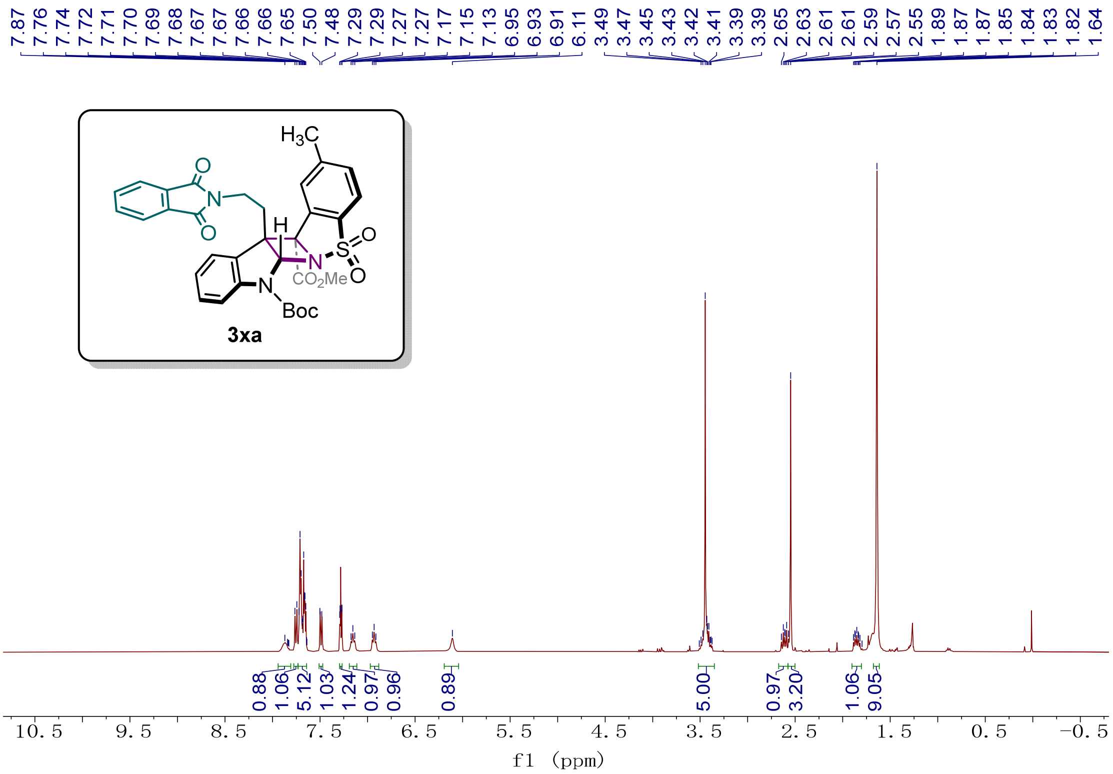

Supplementary Fig. 60. ^{1}H NMR of compound **3xa (400 MHz, CDCl$_3$)**

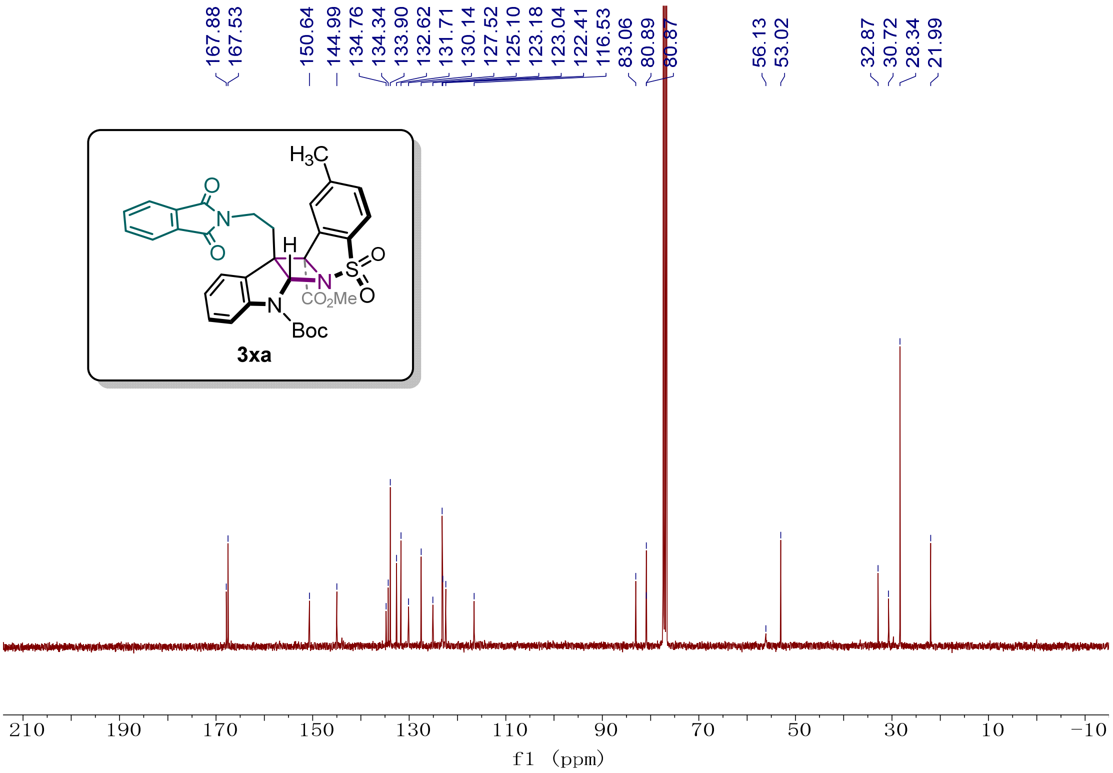

Supplementary Fig. 61. ^{13}C NMR of compound **3xa (101 MHz, CDCl$_3$)**

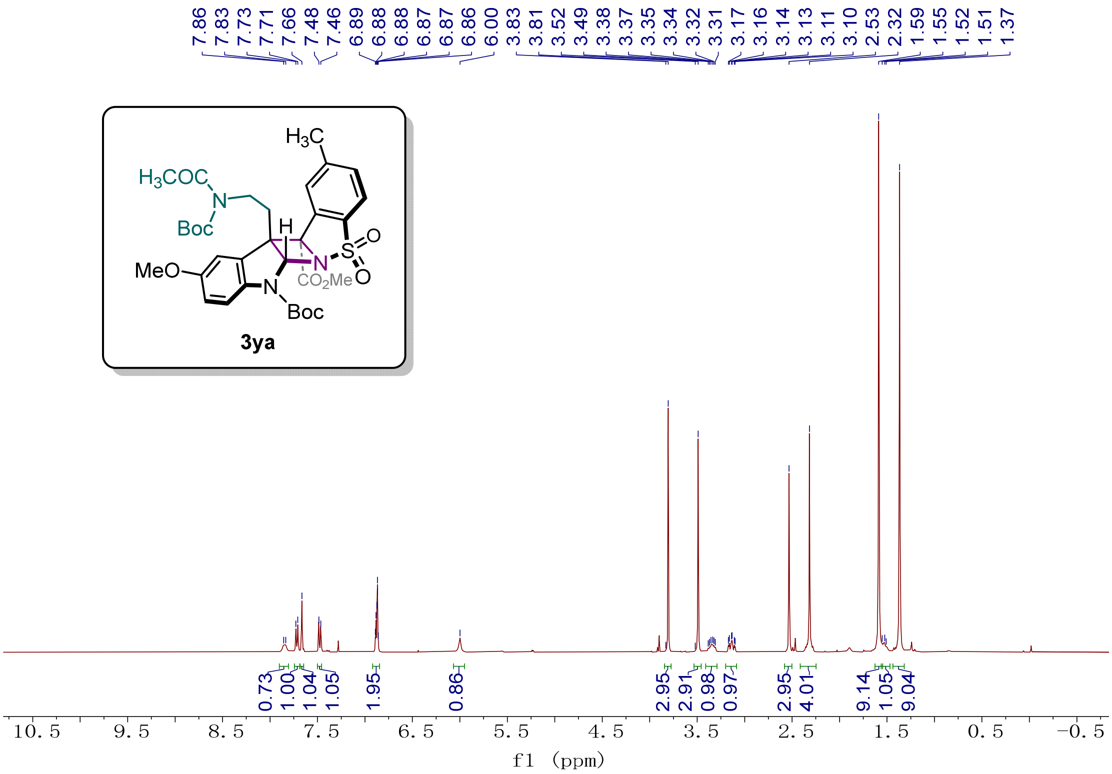

Supplementary Fig. 62. ^{1}H NMR of compound **3ya (400 MHz, CDCl₃)**

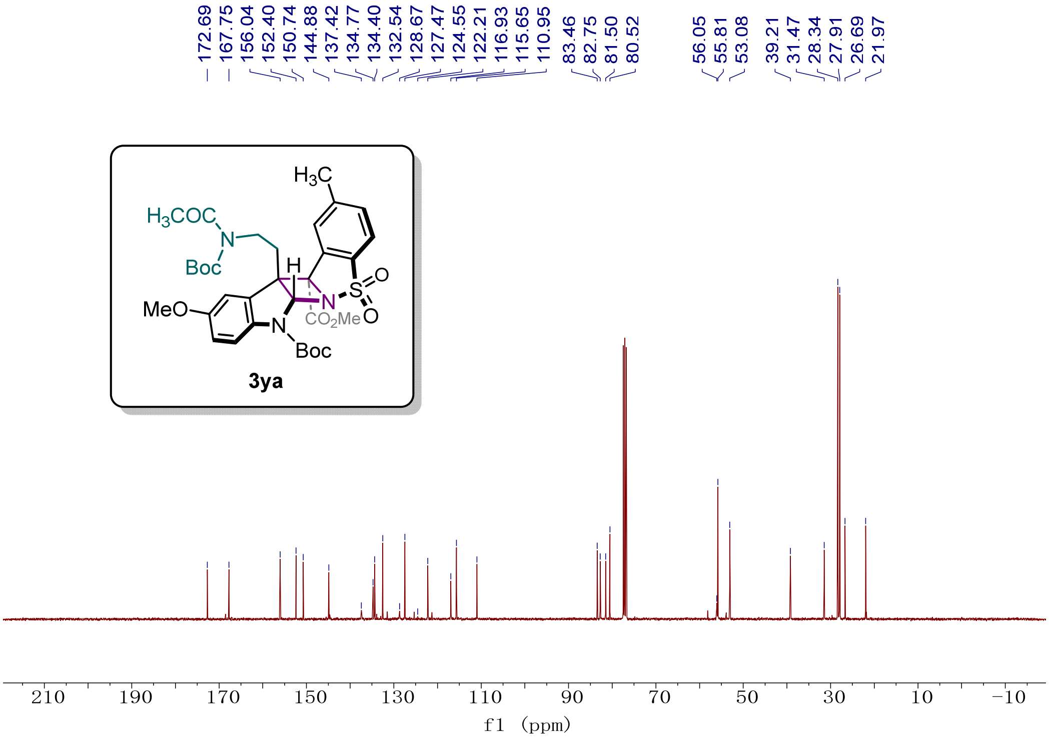

Supplementary Fig. 63. ^{13}C NMR of compound **3ya (101 MHz, CDCl₃)**

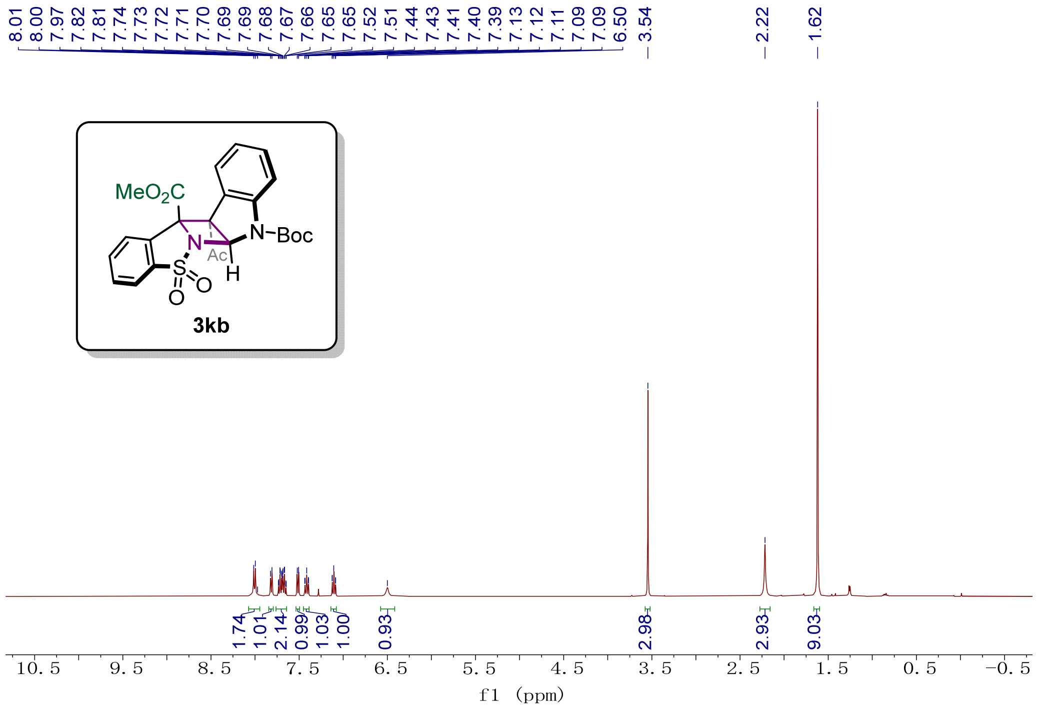

Supplementary Fig. 64. ^{1}H NMR of compound **3kb (400 MHz, $CDCl_3$)**

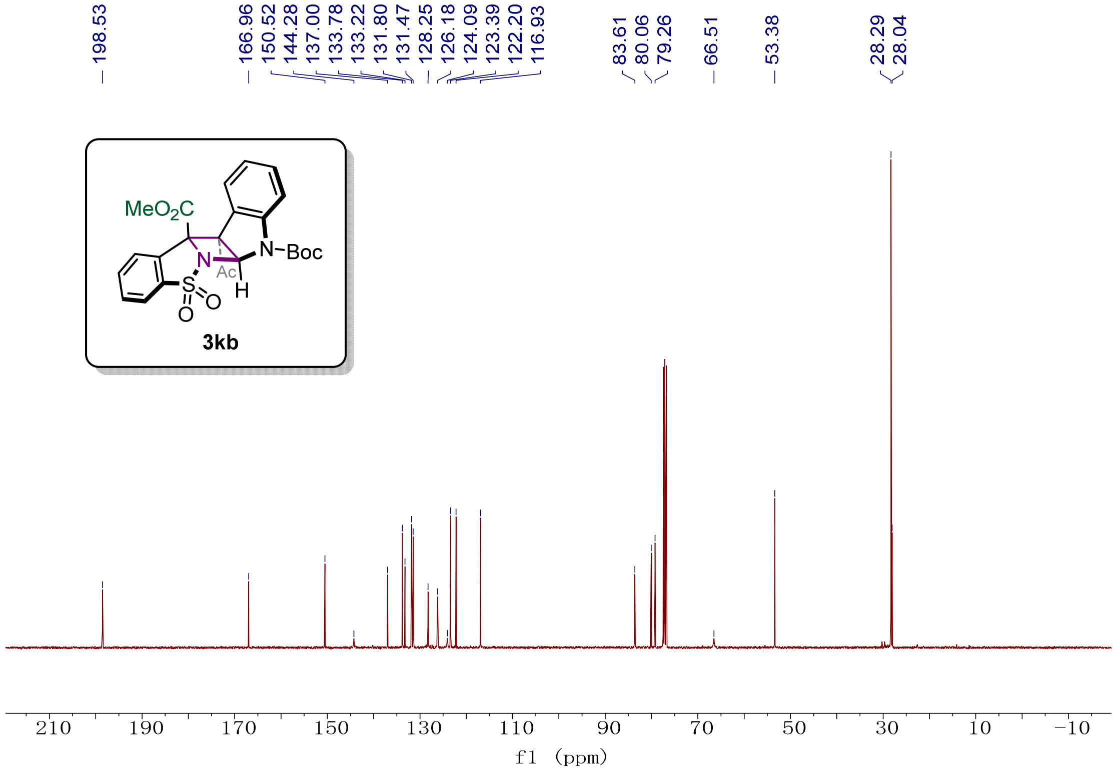

Supplementary Fig. 65. ^{13}C NMR of compound **3kb (101 MHz, $CDCl_3$)**

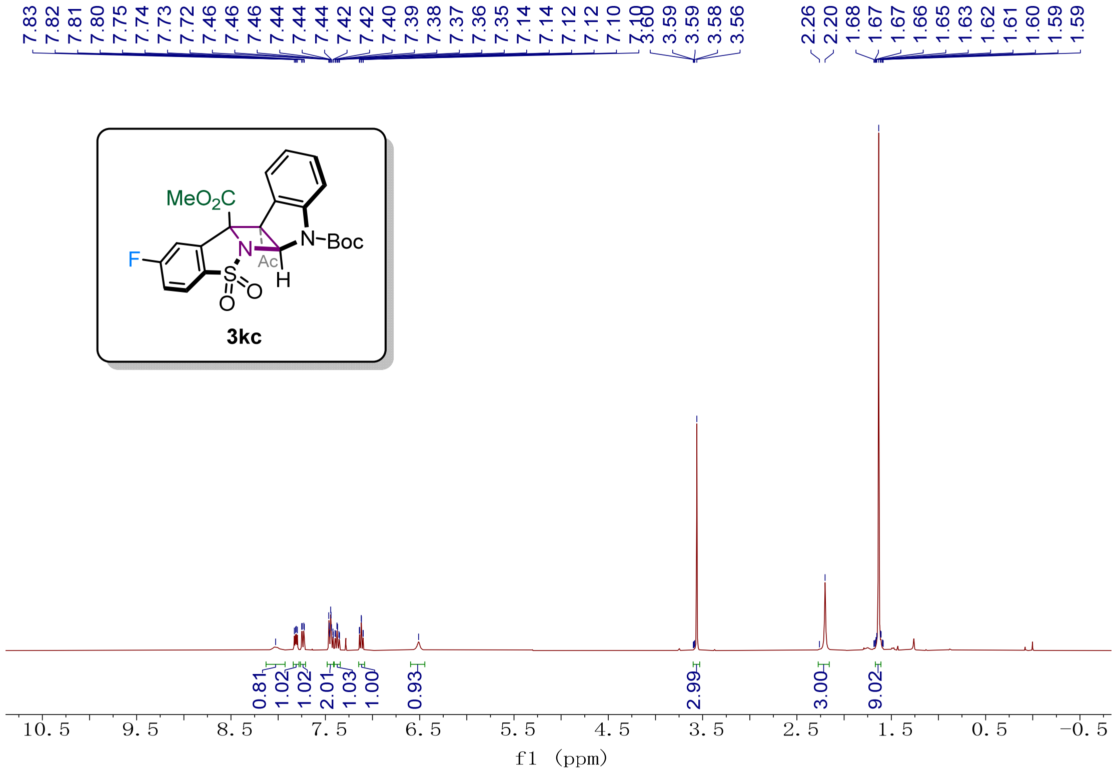

Supplementary Fig. 66. ^{1}H NMR of compound **3kc (400 MHz, CDCl$_3$)**

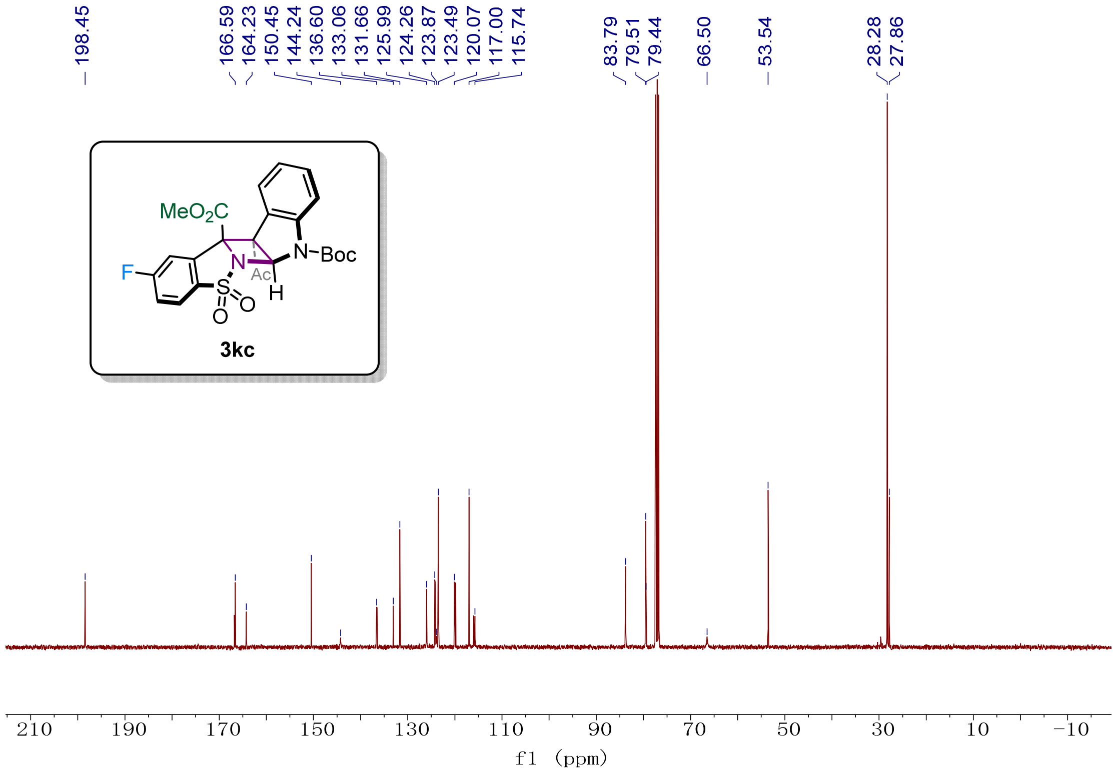

Supplementary Fig. 67. ^{13}C NMR of compound **3kc (101 MHz, CDCl$_3$)**

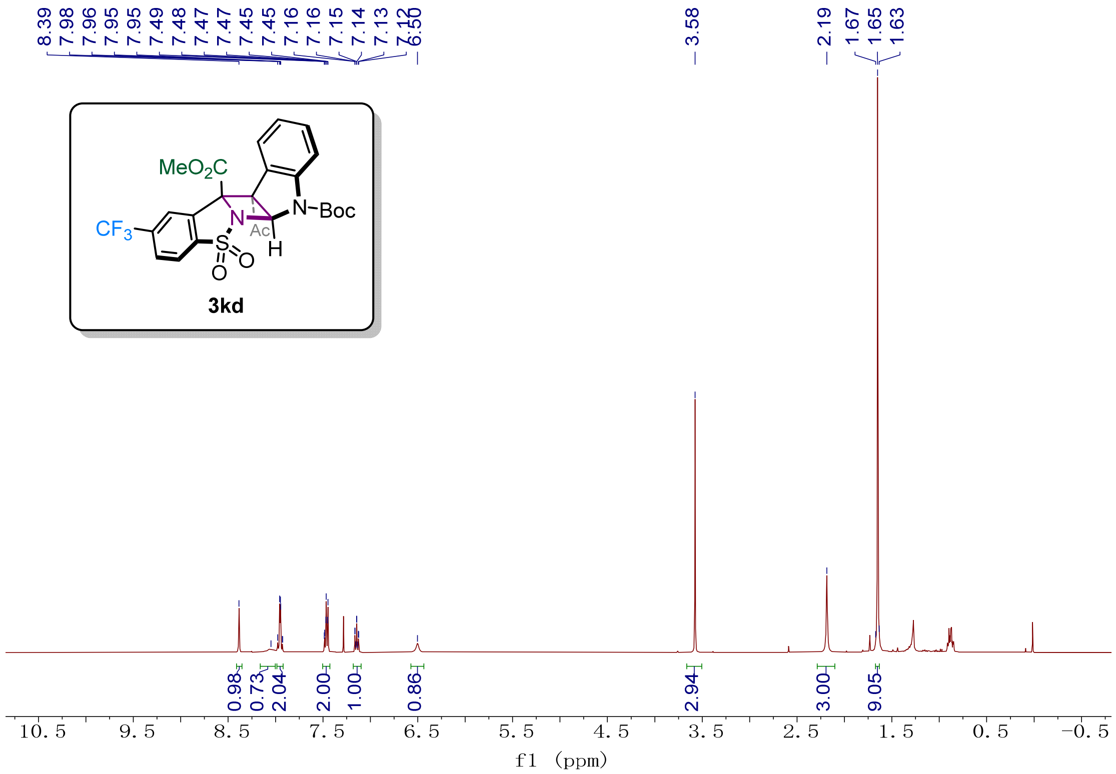

Supplementary Fig. 68. ^{1}H NMR of compound **3kd (400 MHz, CDCl$_3$)**

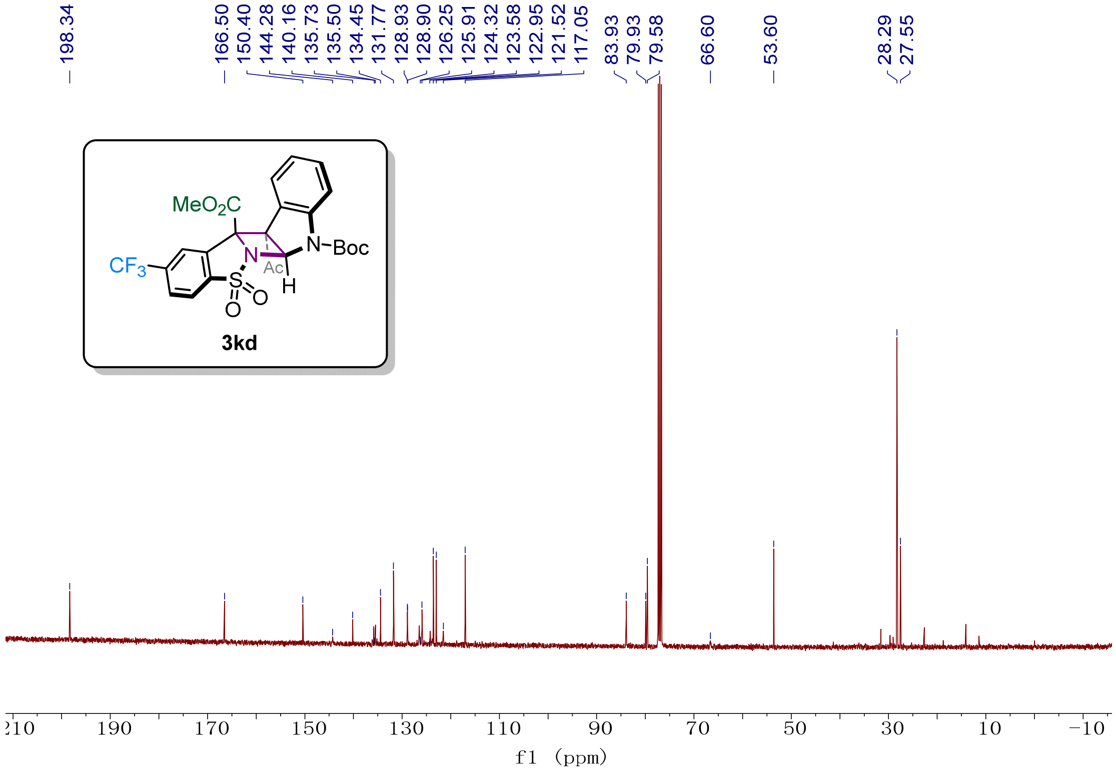

Supplementary Fig. 69. ^{13}C NMR of compound **3kd (101 MHz, CDCl$_3$)**

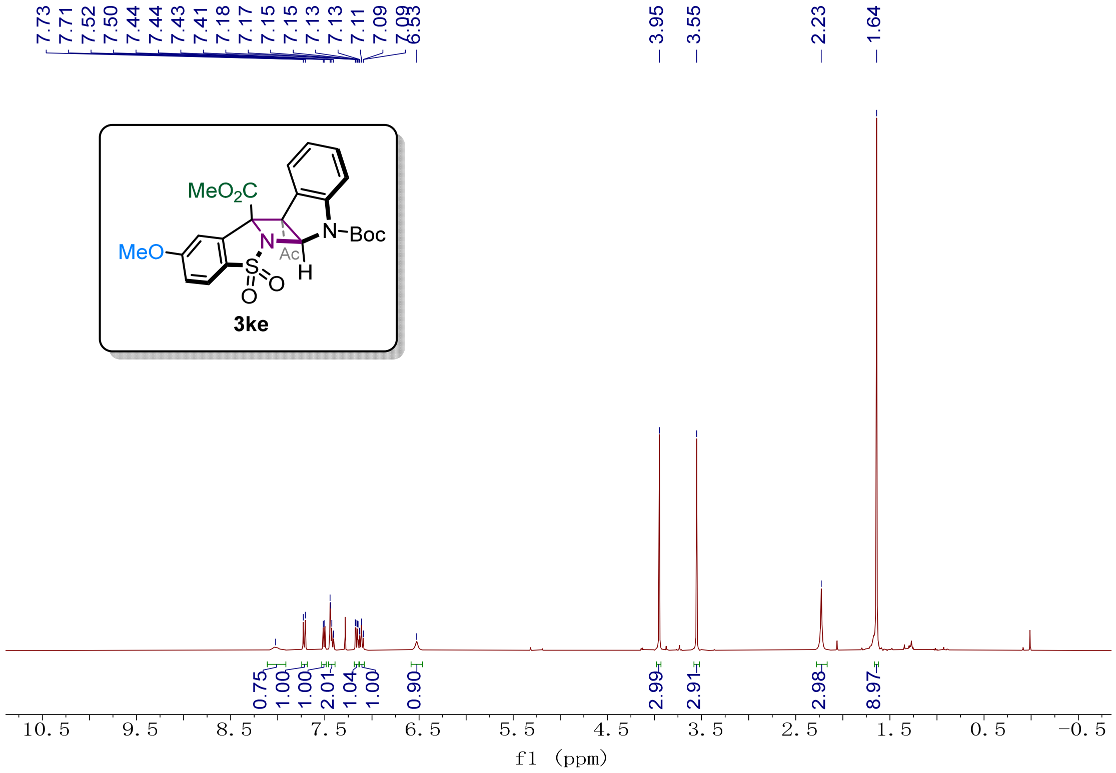

Supplementary Fig. 70. ^{1}H NMR of compound **3ke (400 MHz, CDCl$_3$)**

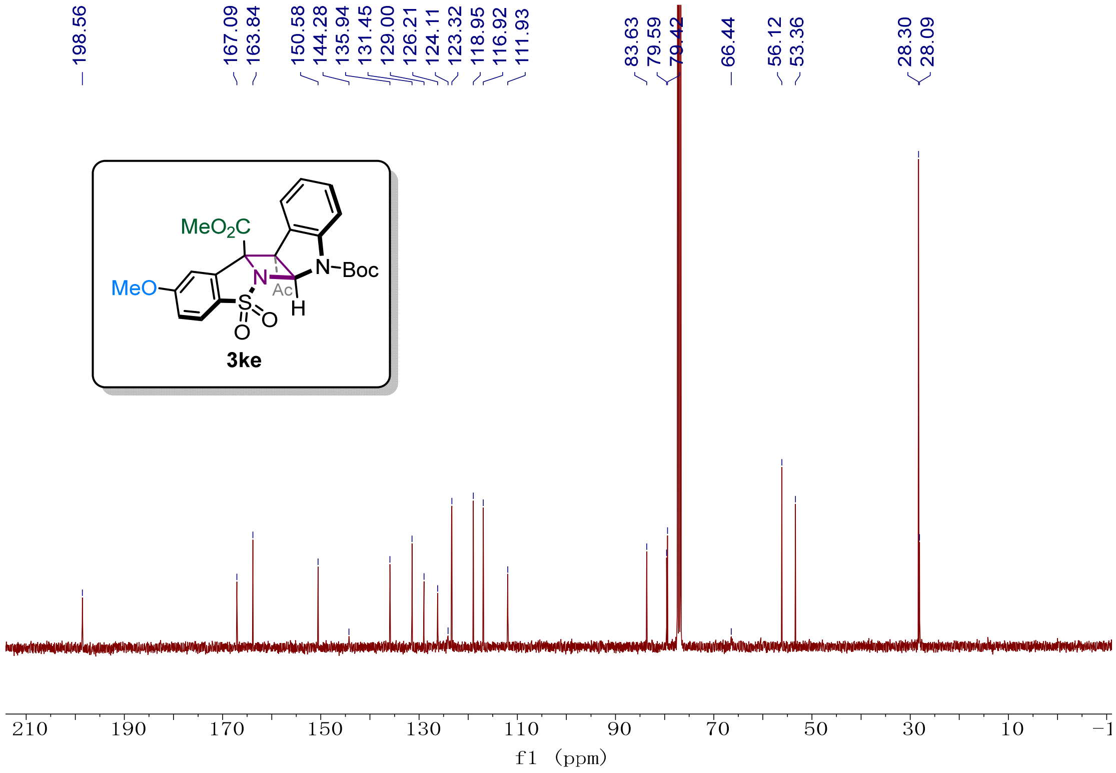

Supplementary Fig. 71. ^{13}C NMR of compound **3ke (101 MHz, CDCl$_3$)**

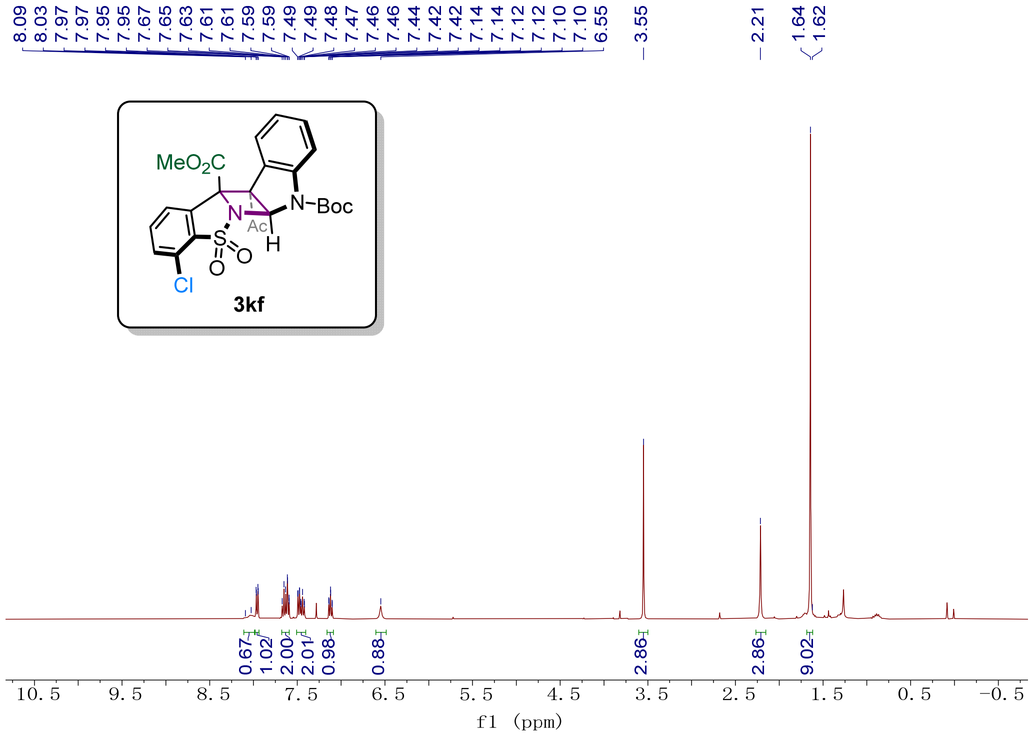

Supplementary Fig. 72. ^{1}H NMR of compound **3kf (400 MHz, CDCl$_3$)**

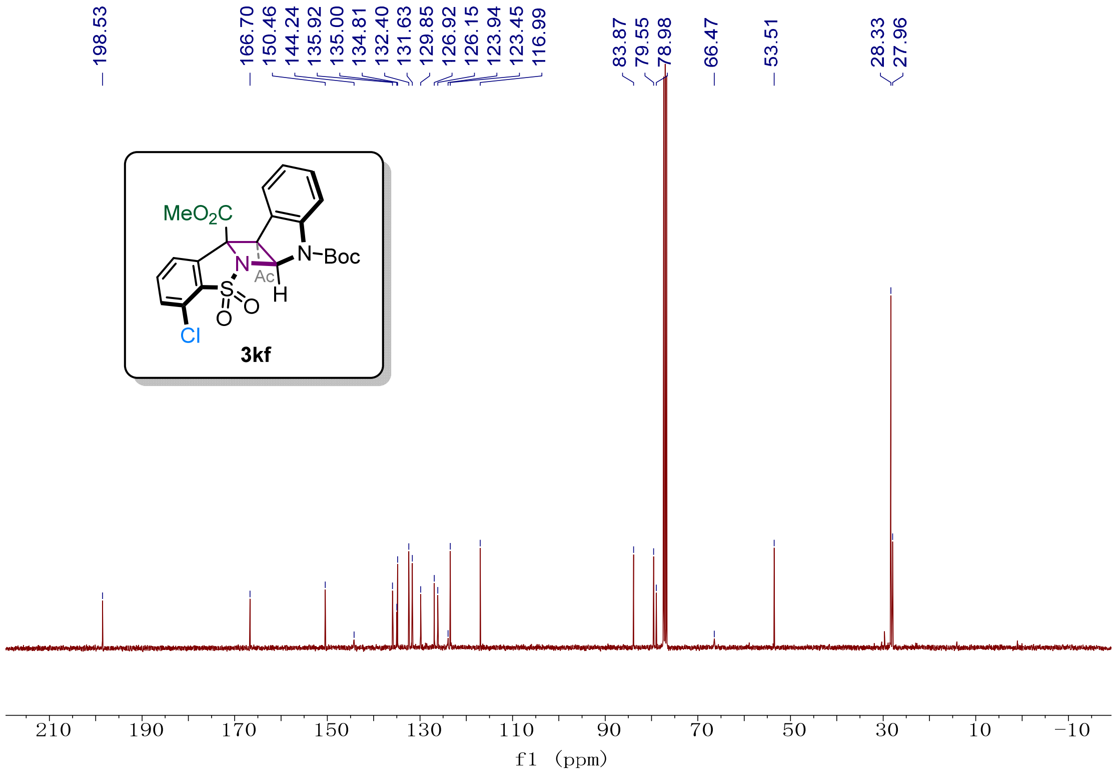

Supplementary Fig. 73. ^{13}C NMR of compound **3kf (101 MHz, CDCl$_3$)**

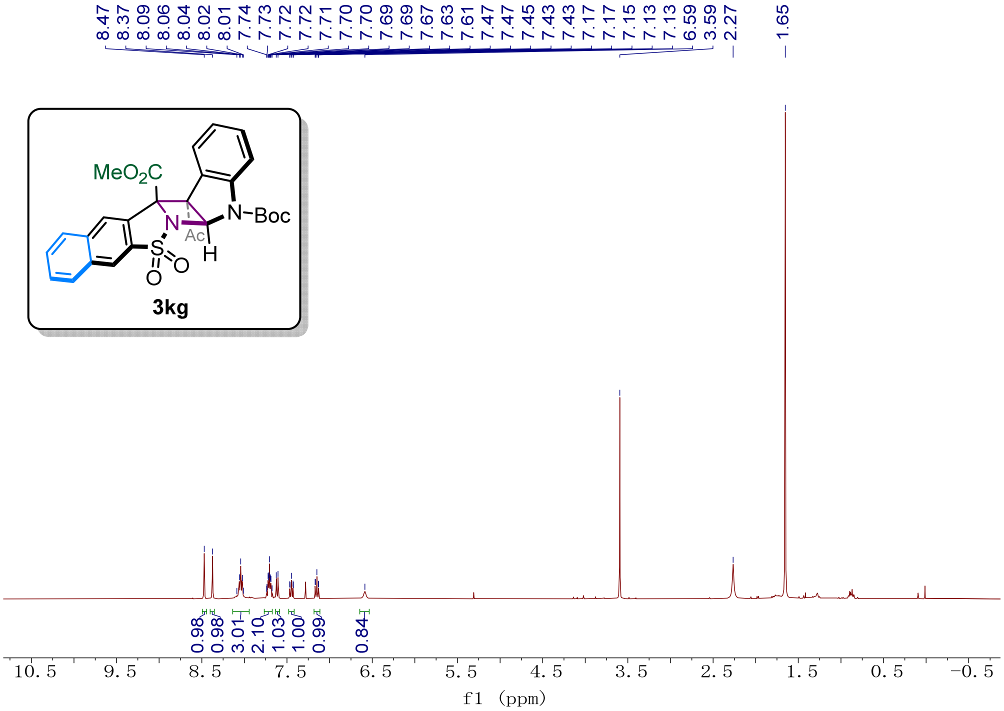

Supplementary Fig. 74. ^{1}H NMR of compound **3kg (400 MHz, $CDCl_3$)**

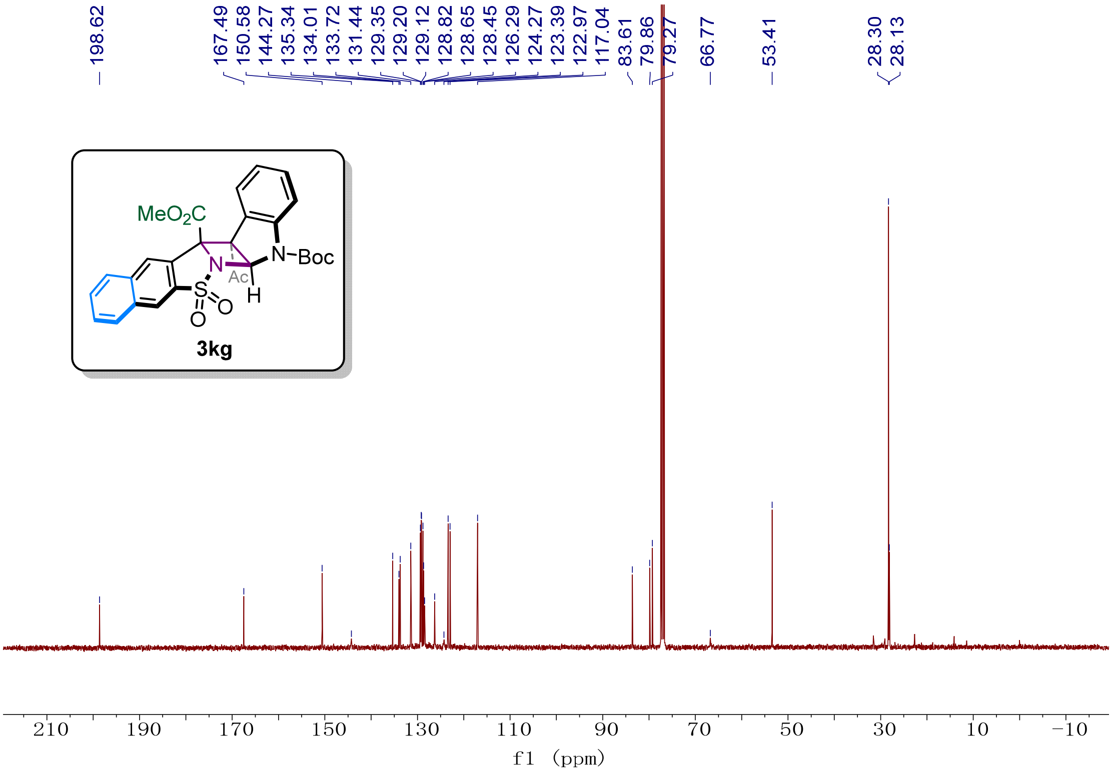

Supplementary Fig. 75. ^{13}C NMR of compound **3kg (101 MHz, $CDCl_3$)**

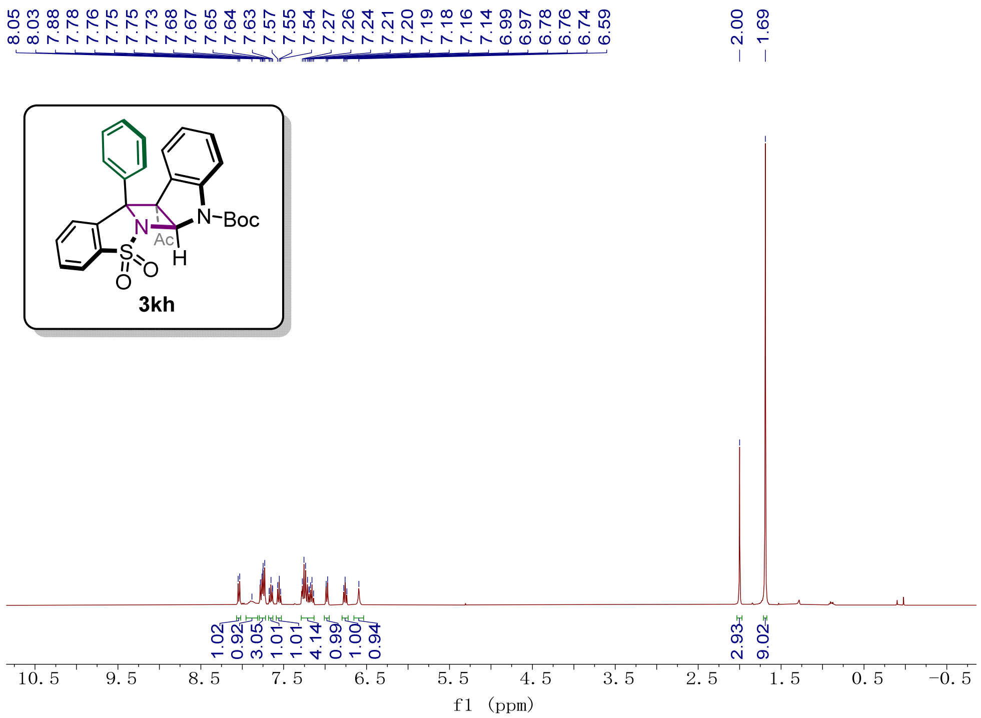

Supplementary Fig. 76. ^{1}H NMR of compound **3kh (400 MHz, CDCl$_3$)**

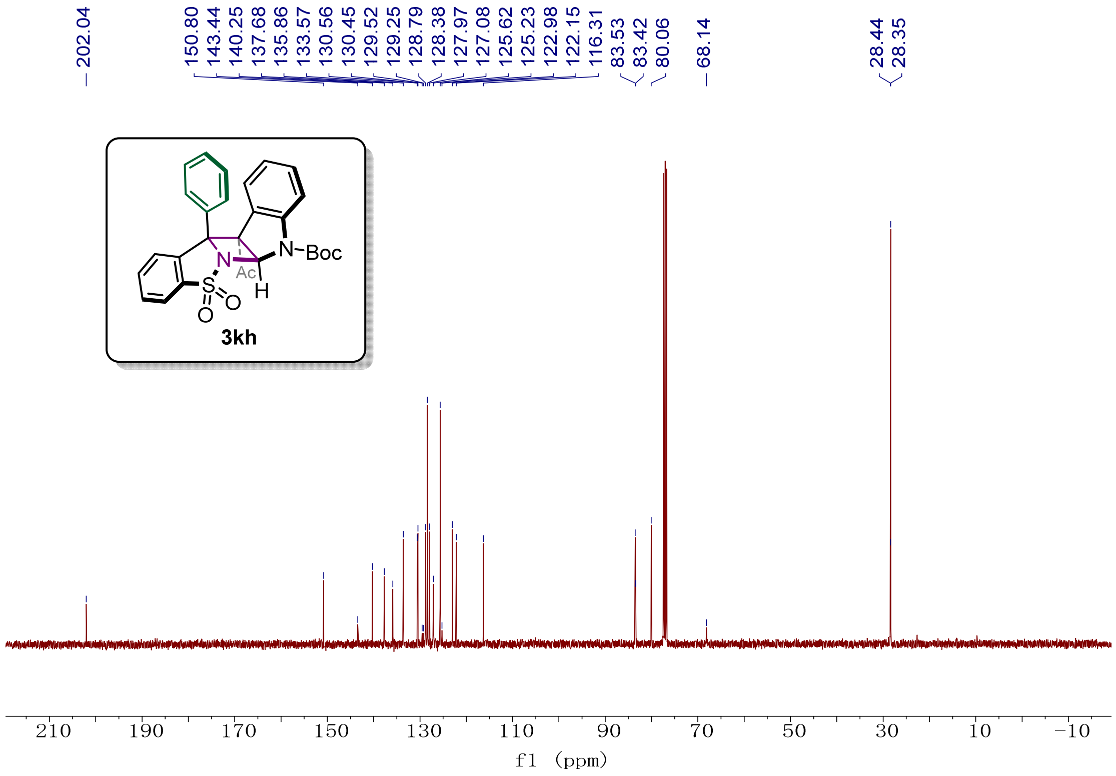

Supplementary Fig. 77. ^{13}C NMR of compound **3kh(101 MHz, CDCl$_3$)**

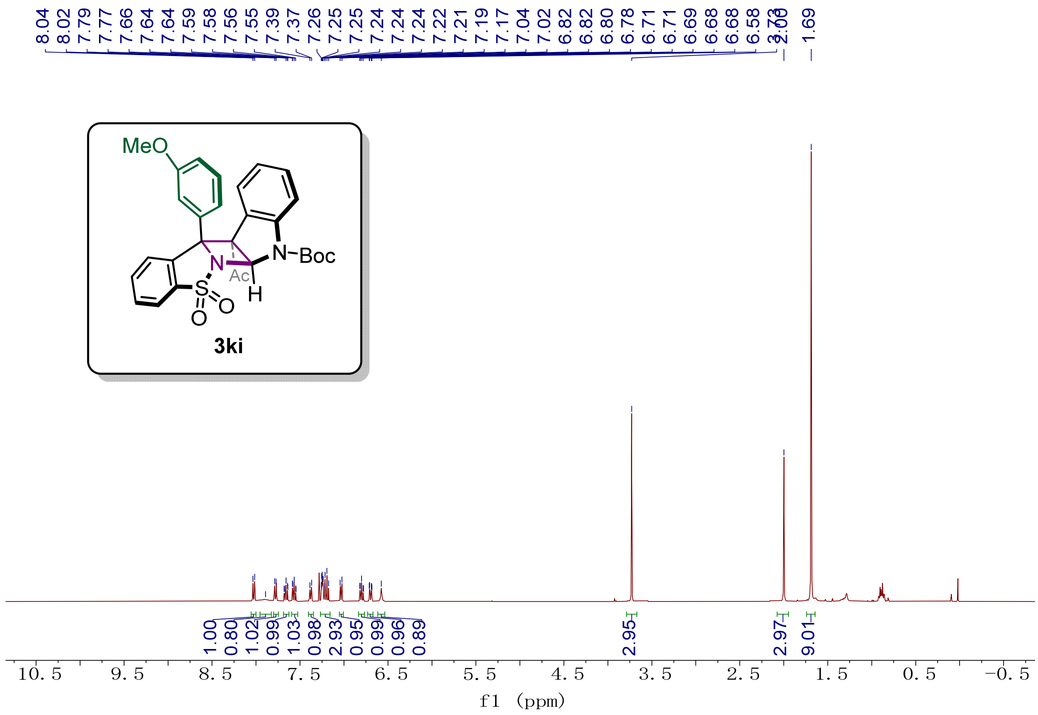

Supplementary Fig. 78. ^{1}H NMR of compound **3ki (400 MHz, CDCl$_3$)**

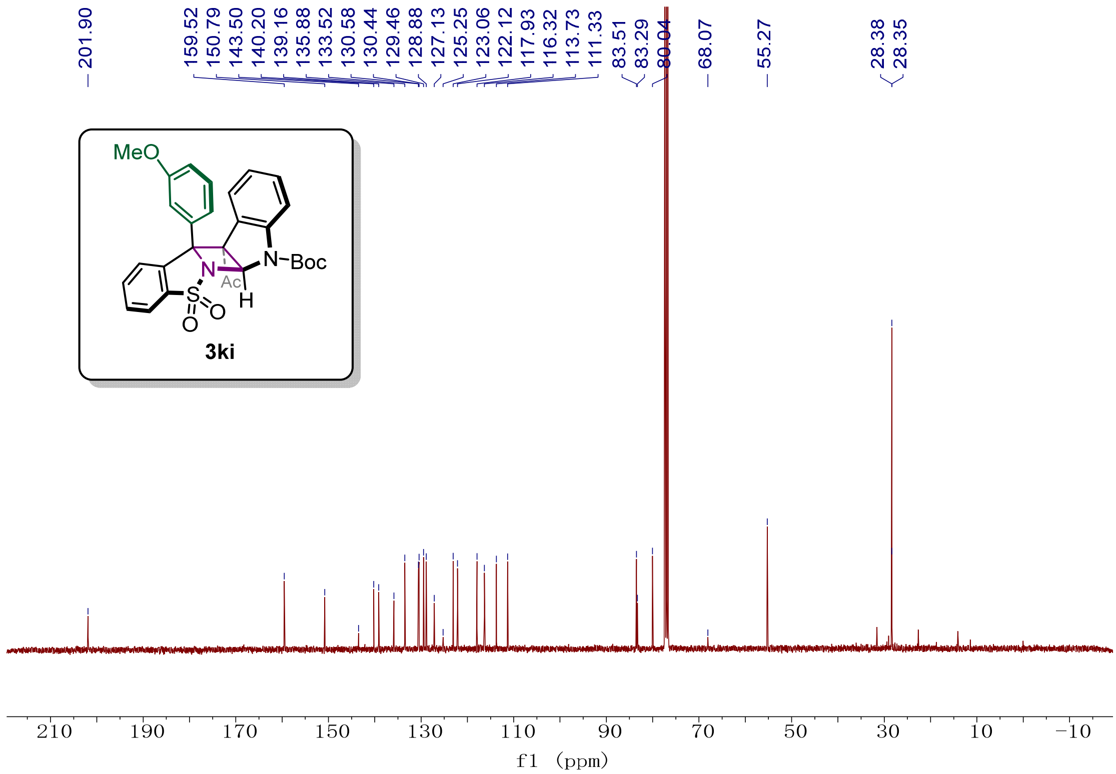

Supplementary Fig. 79. ^{13}C NMR of compound **3ki (101 MHz, CDCl$_3$)**

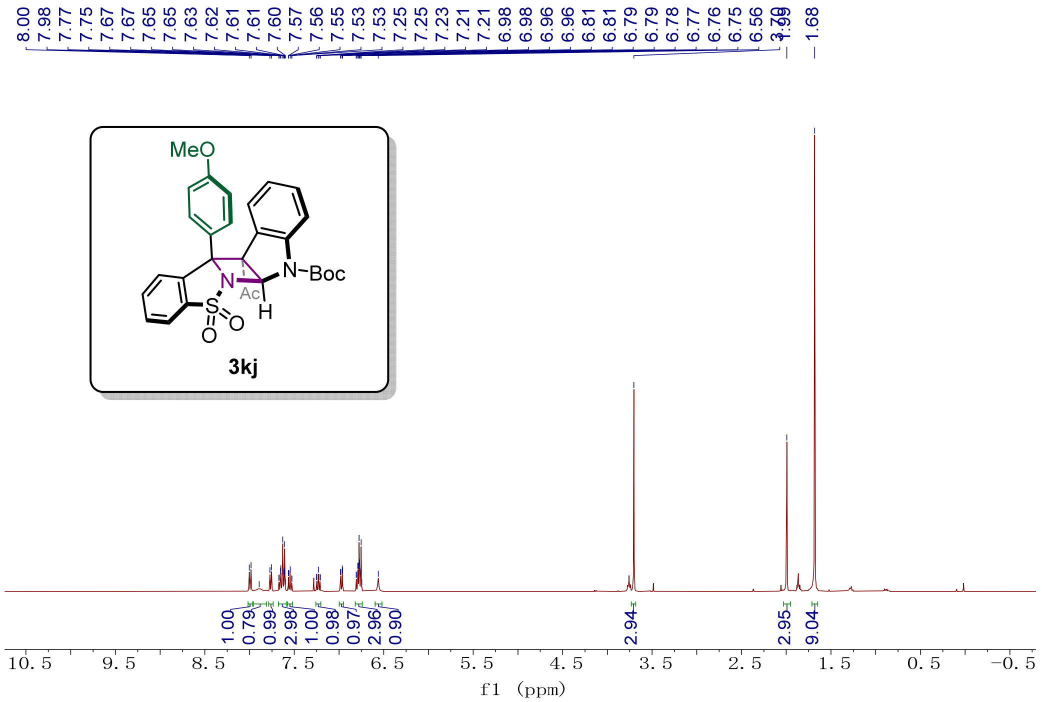

Supplementary Fig. 80. ^{1}H NMR of compound **3kj (400 MHz, CDCl$_3$)**

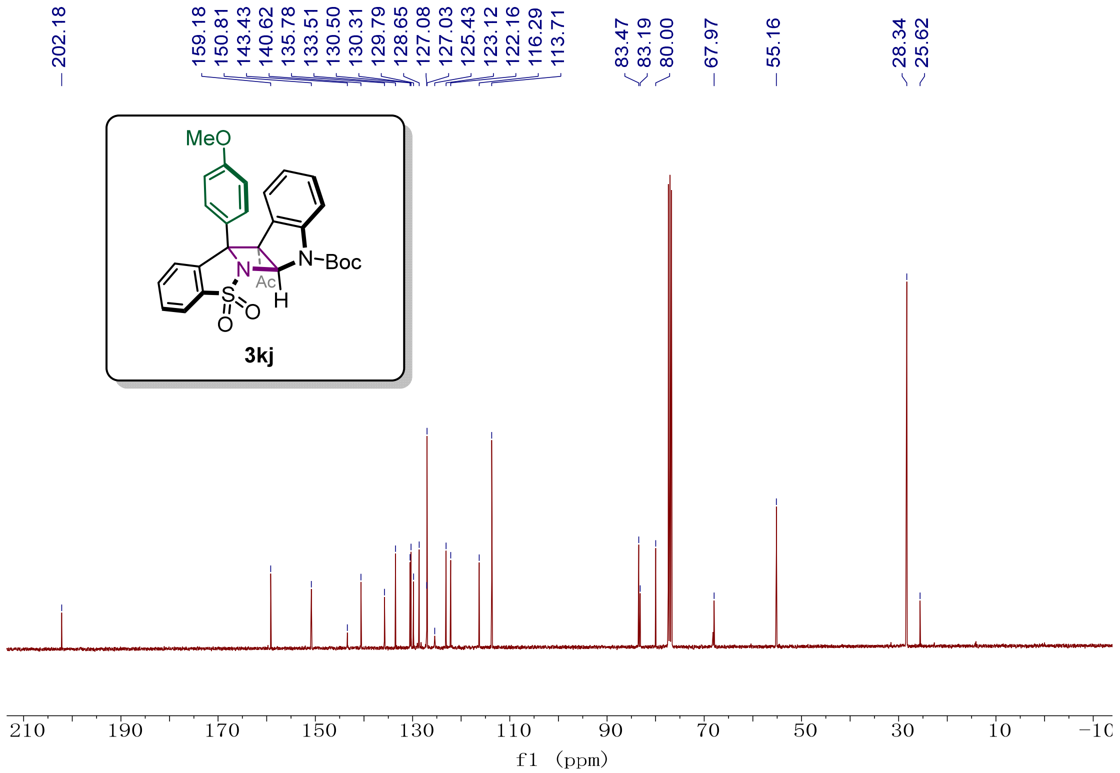

Supplementary Fig. 81. ^{13}C NMR of compound **3kj (101 MHz, CDCl$_3$)**

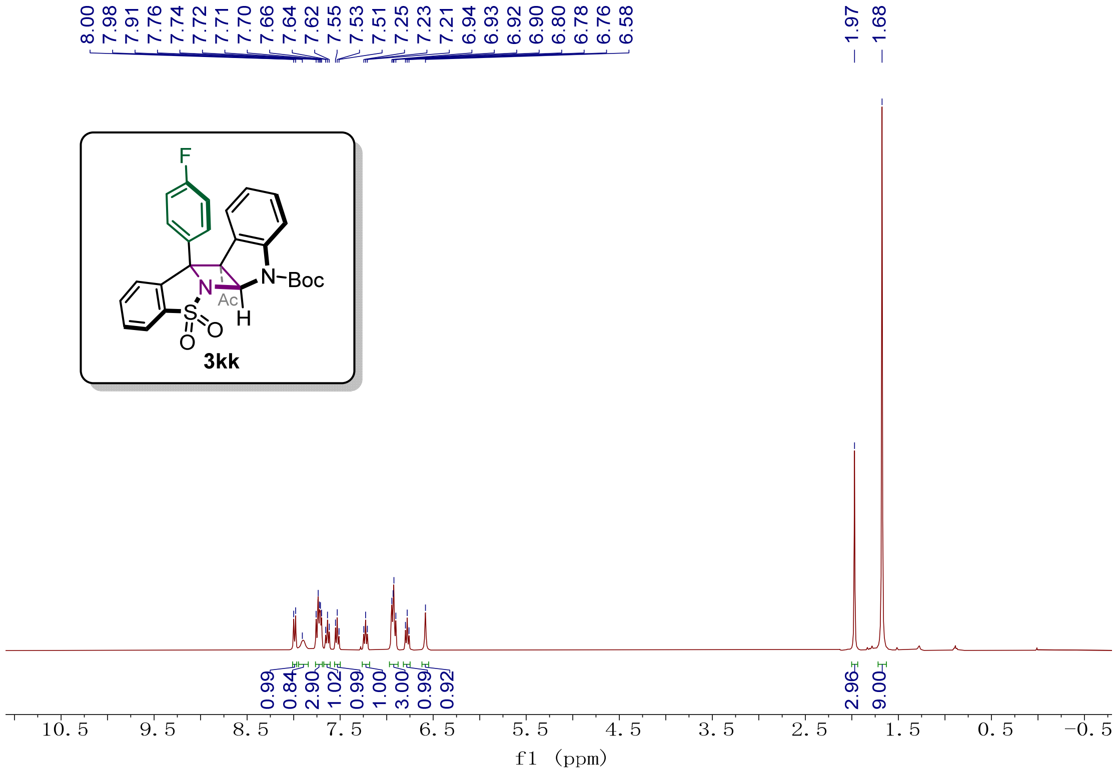

Supplementary Fig. 82. ^{1}H NMR of compound **3kk (400 MHz, CDCl$_3$)**

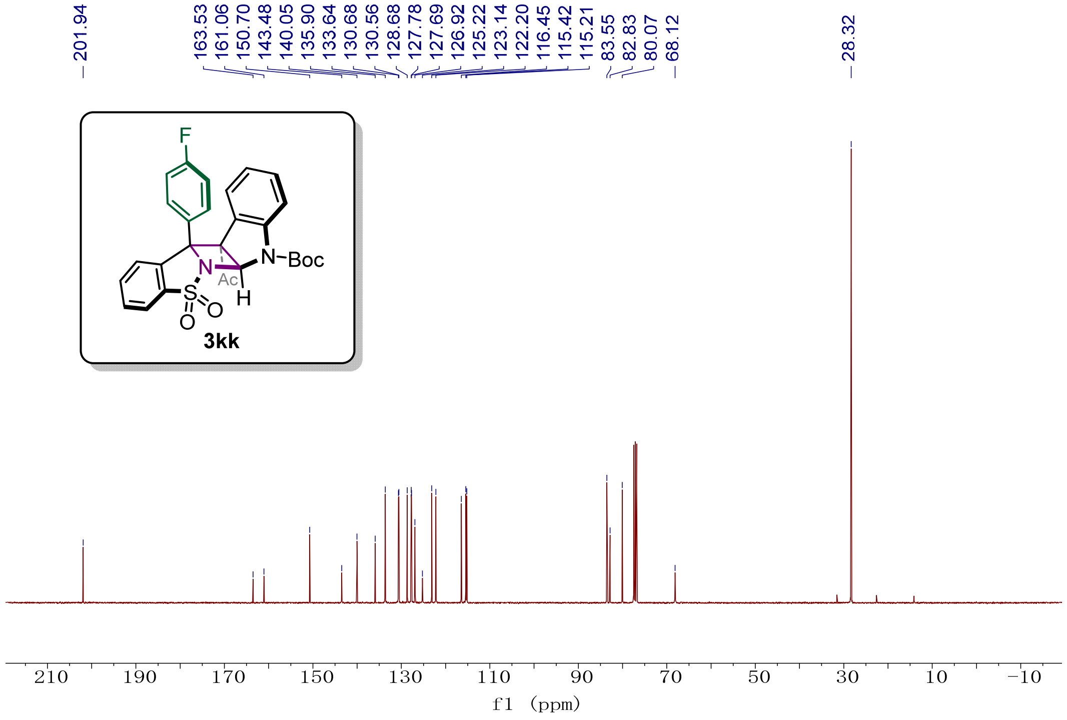

Supplementary Fig. 83. ^{13}C NMR of compound **3kk (101 MHz, CDCl$_3$)**

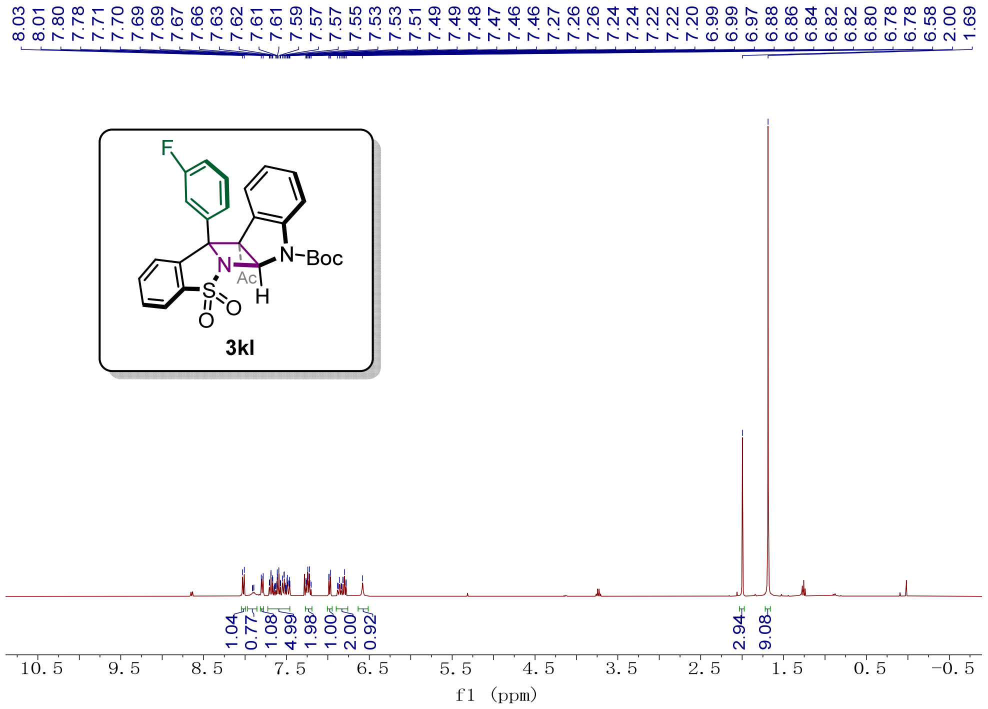

Supplementary Fig. 84. ^{1}H NMR of compound **3kl (400 MHz, CDCl$_3$)**

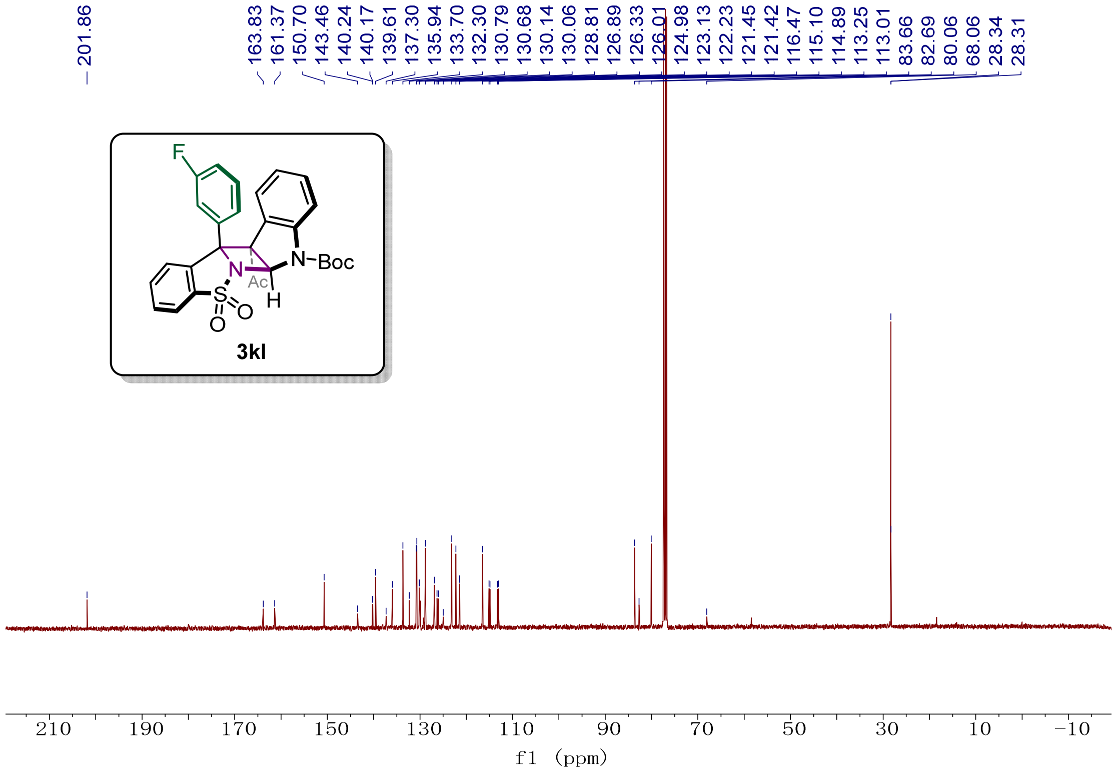

Supplementary Fig. 85. ^{13}C NMR of compound **3kl (101 MHz, CDCl$_3$)**

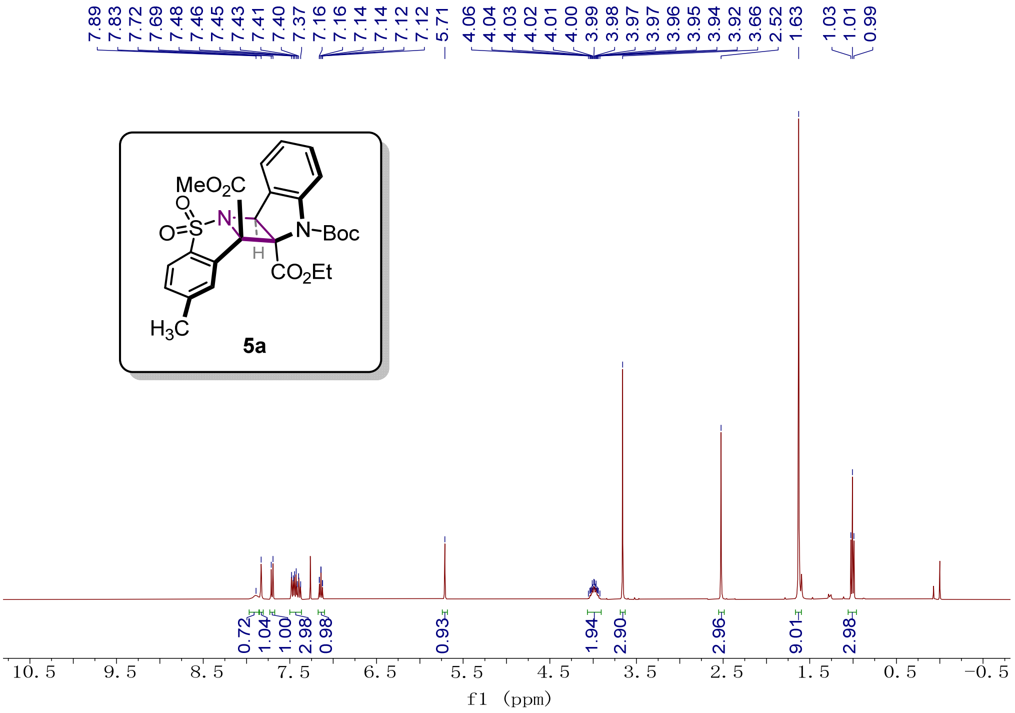

Supplementary Fig. 86. ^{1}H NMR of compound **5a (400 MHz, $CDCl_3$)**

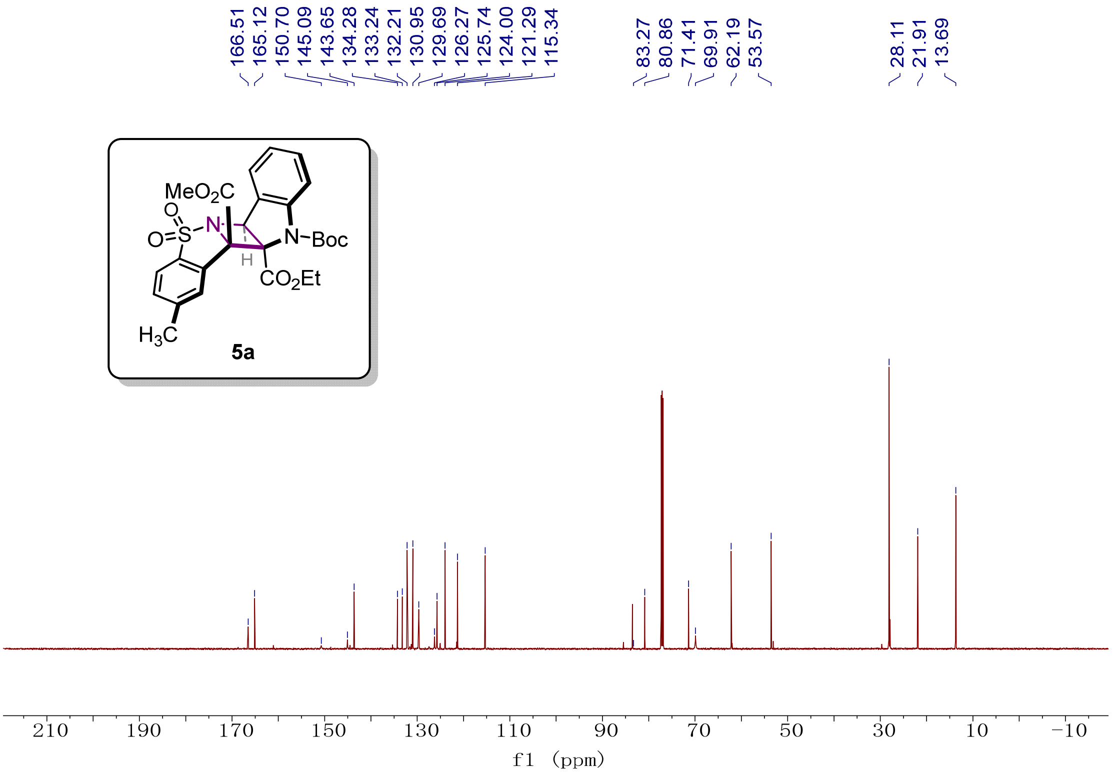

Supplementary Fig. 87. ^{13}C NMR of compound **5a (101 MHz, $CDCl_3$)**

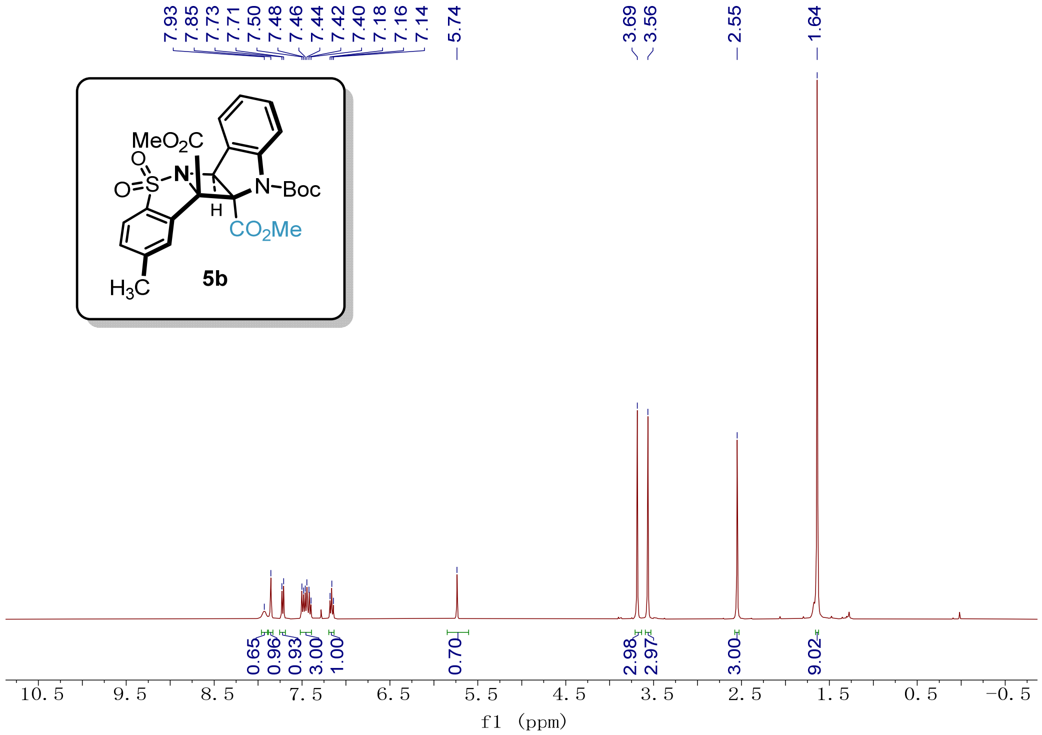

Supplementary Fig. 88. ^{1}H NMR of compound **5b (400 MHz, CDCl₃)**

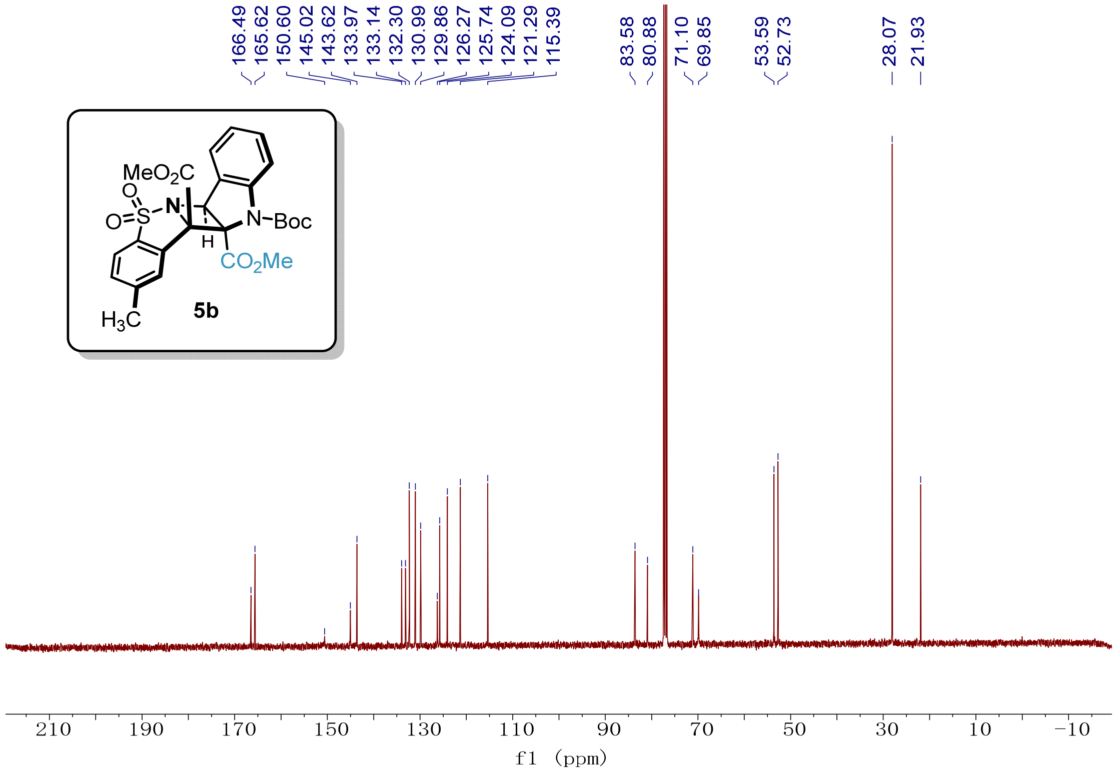

Supplementary Fig. 89. ^{13}C NMR of compound **5b (101 MHz, CDCl₃)**

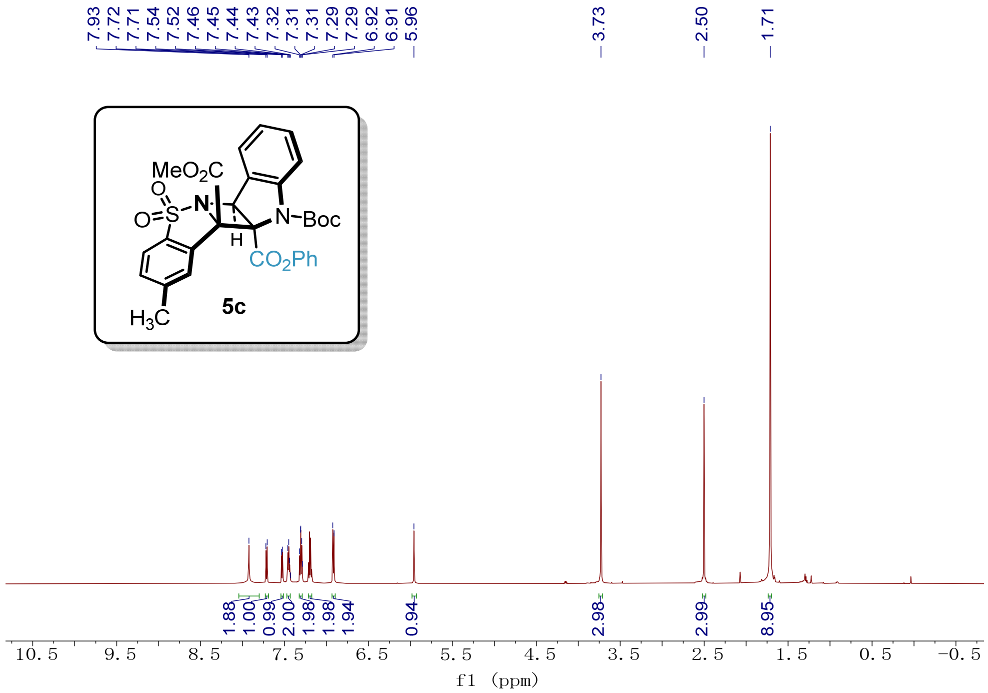

Supplementary Fig. 90. ^{1}H NMR of compound **5c (600 MHz, CDCl$_3$)**

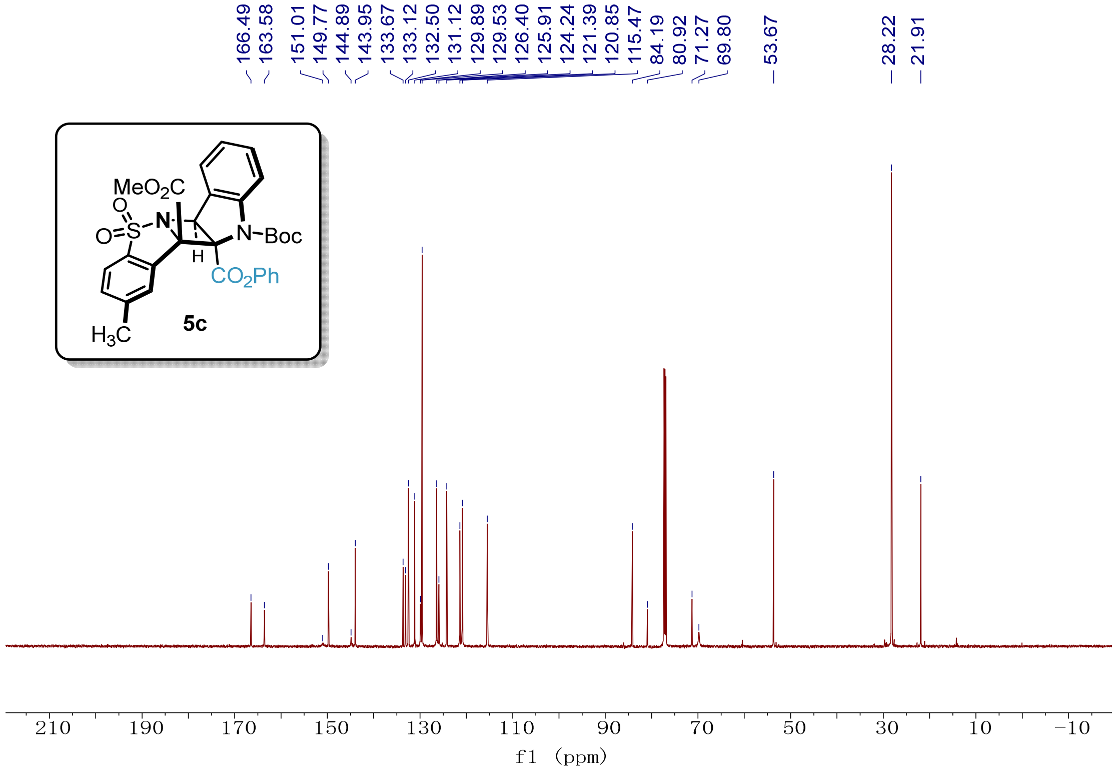

Supplementary Fig. 91. ^{13}C NMR of compound **5c (151 MHz, CDCl$_3$)**

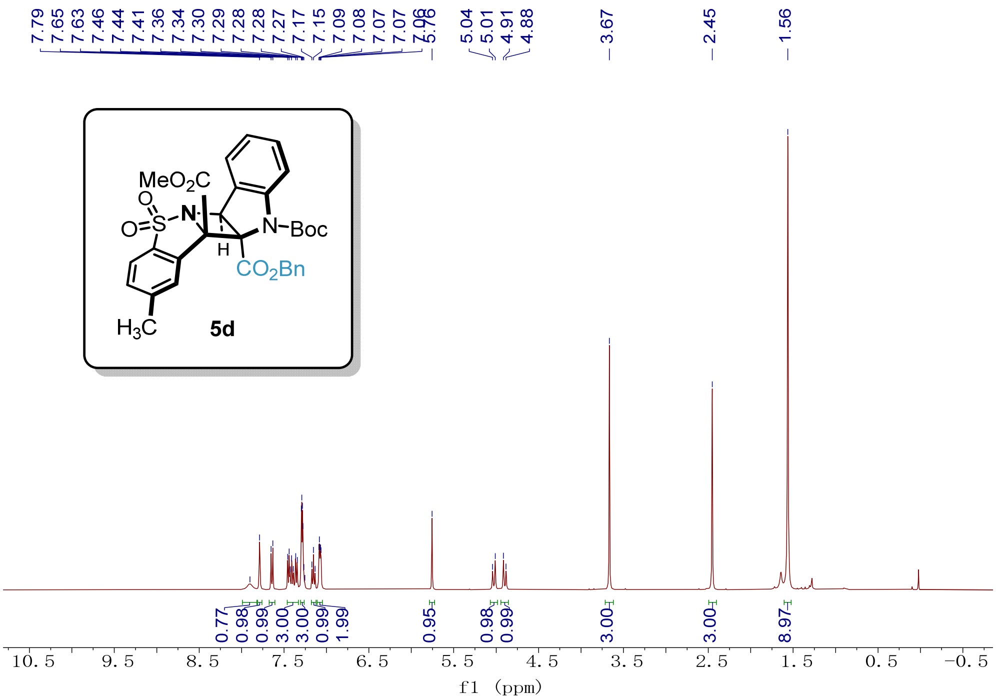

Supplementary Fig. 92. ^{1}H NMR of compound **5d (400 MHz, CDCl$_3$)**

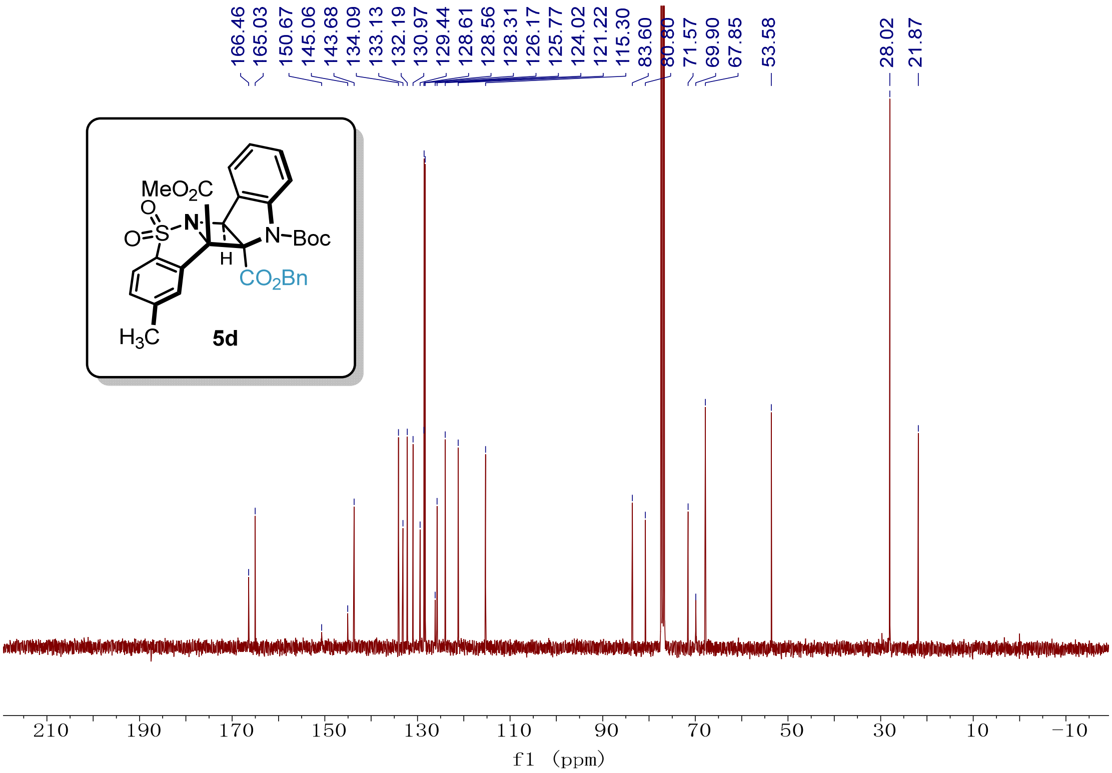

Supplementary Fig. 93. ^{13}C NMR of compound **5d (101 MHz, CDCl$_3$)**

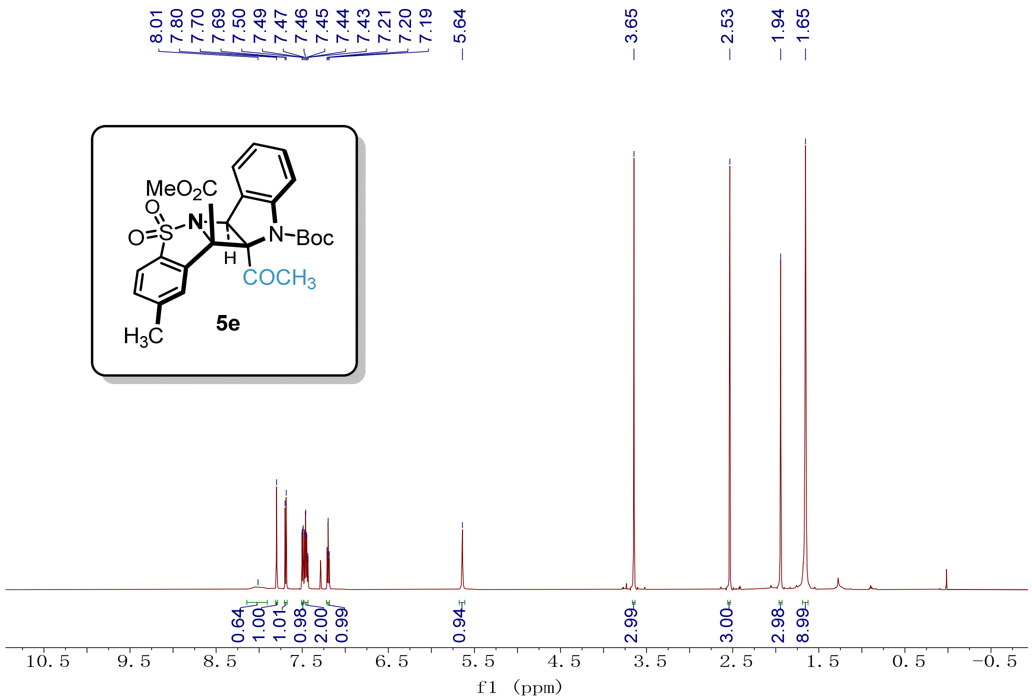

Supplementary Fig. 94. ^{1}H NMR of compound **5e (600 MHz, CDCl$_3$)**

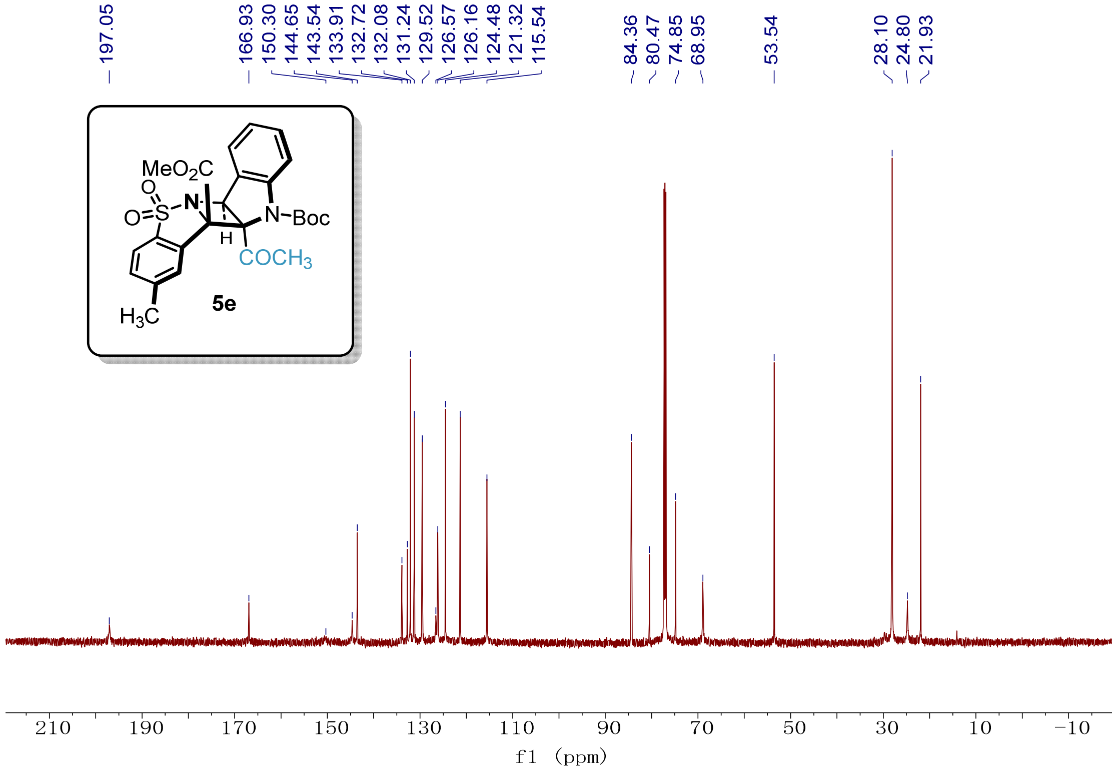

Supplementary Fig. 95. ^{13}C NMR of compound **5e (151 MHz, CDCl$_3$)**

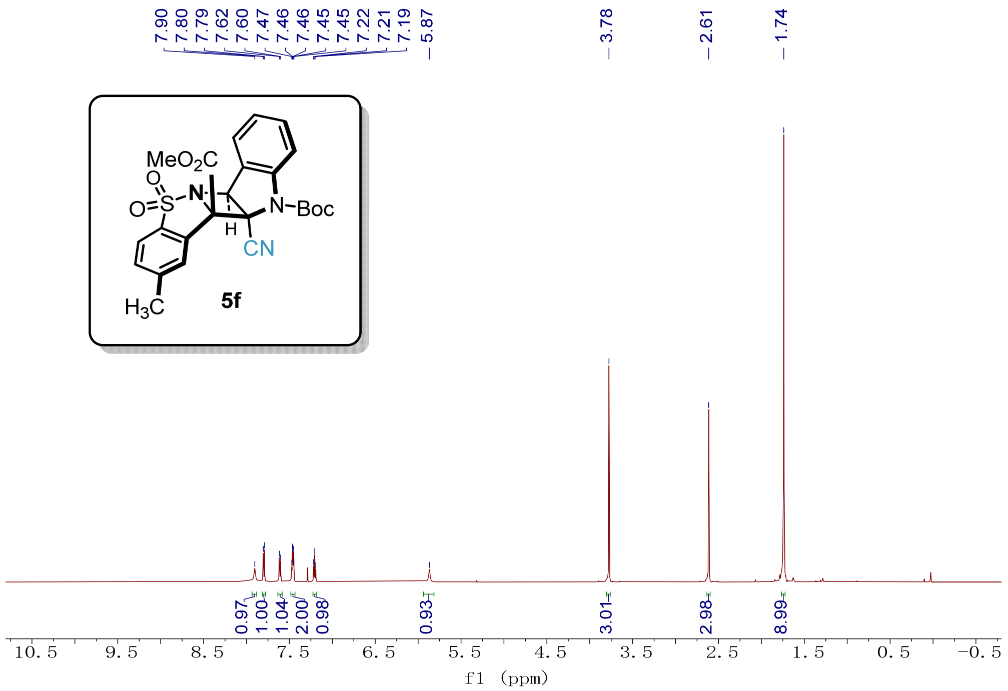

Supplementary Fig. 96. ^{1}H NMR of compound **5f (600 MHz, $CDCl_3$)**

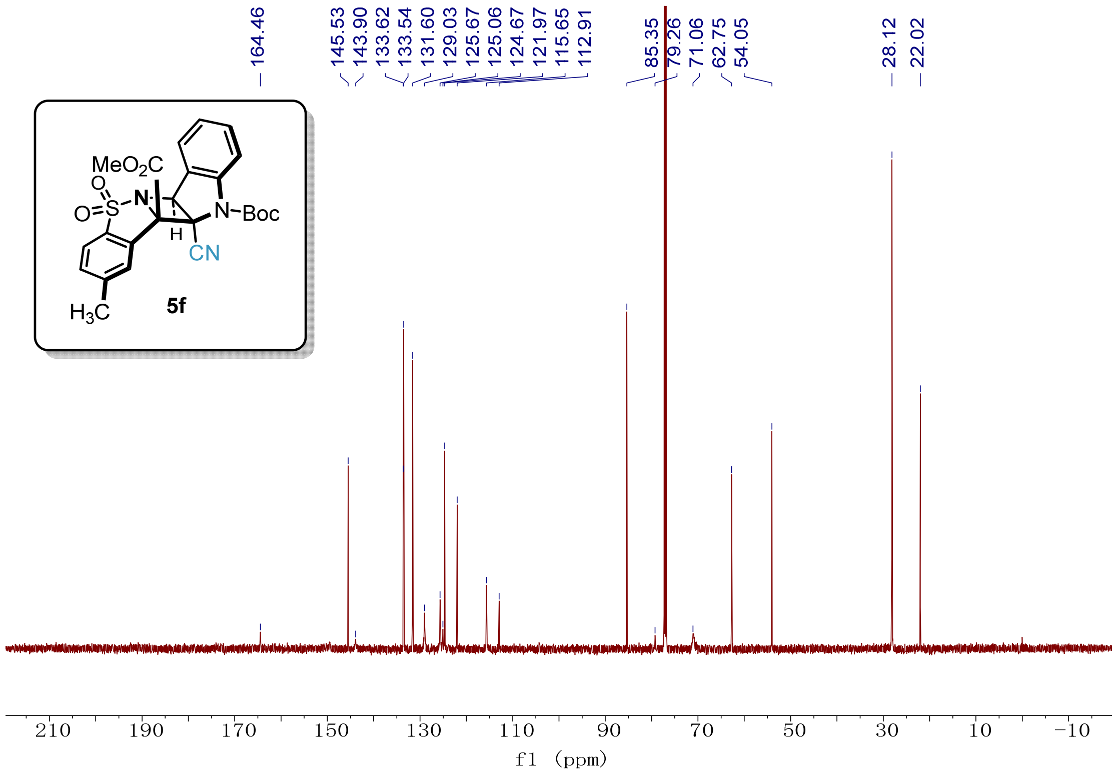

Supplementary Fig. 97. ^{13}C NMR of compound **5f (151 MHz, $CDCl_3$)**

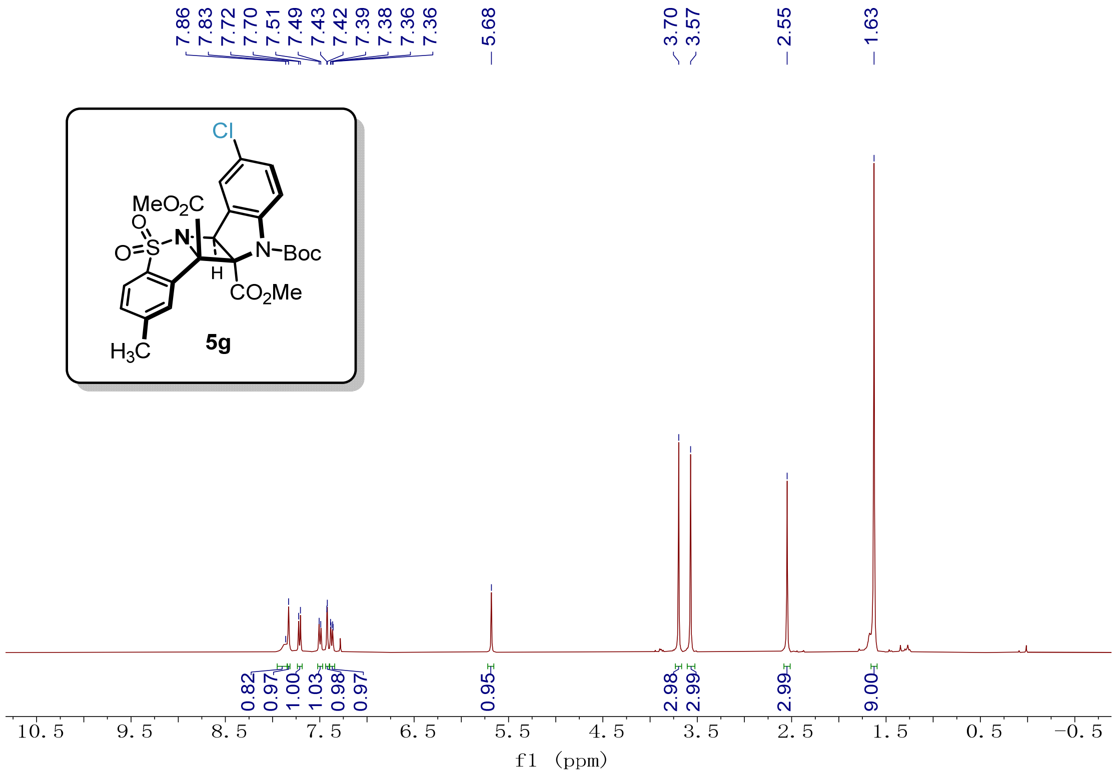

Supplementary Fig. 98. ^{1}H NMR of compound **5g (400 MHz, CDCl$_3$)**

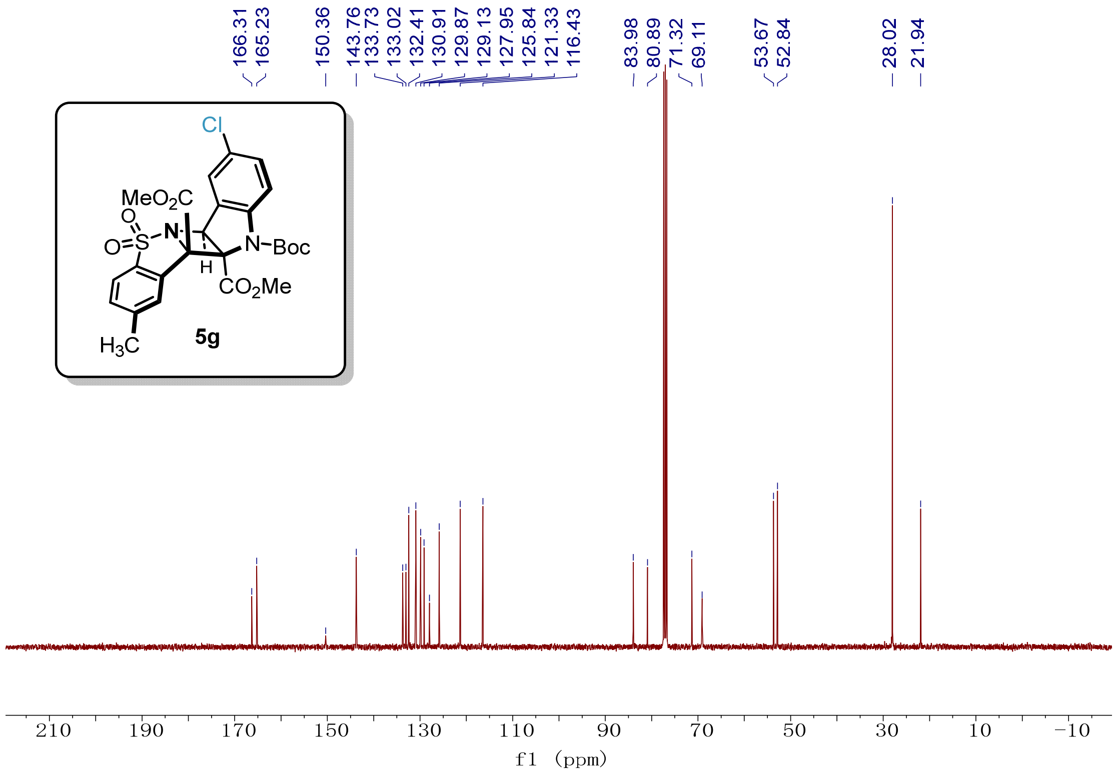

Supplementary Fig. 99. ^{13}C NMR of compound **5g (101 MHz, CDCl$_3$)**

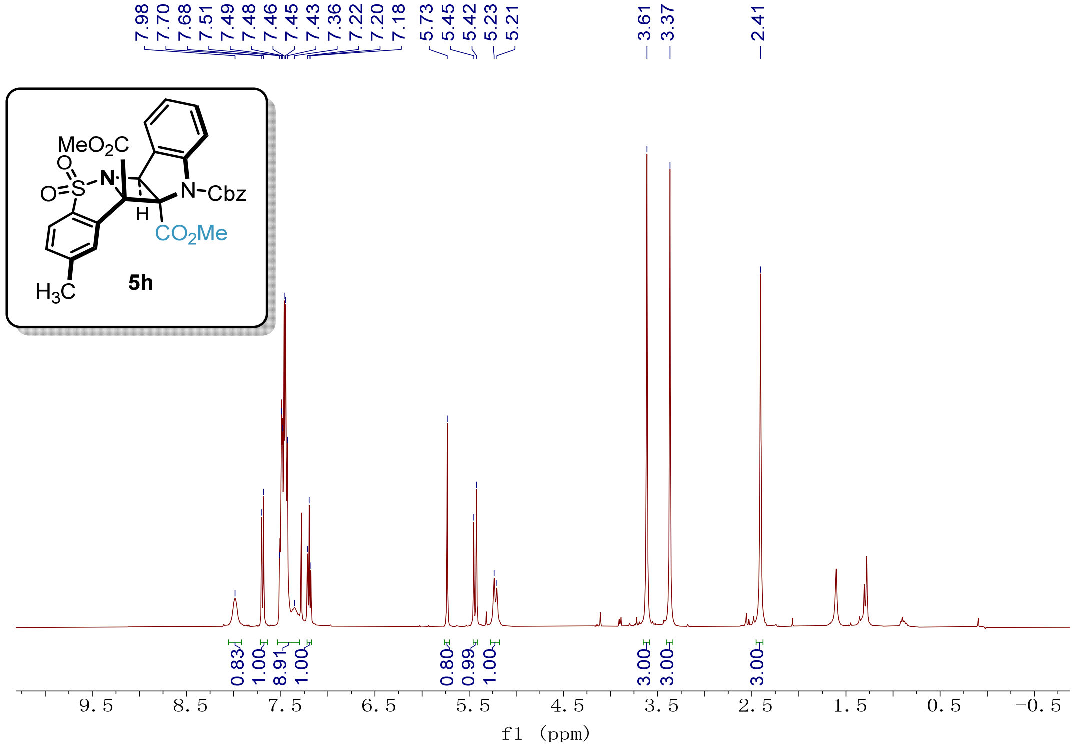

Supplementary Fig. 100. ^{1}H NMR of compound **5h (400 MHz, CDCl₃)**

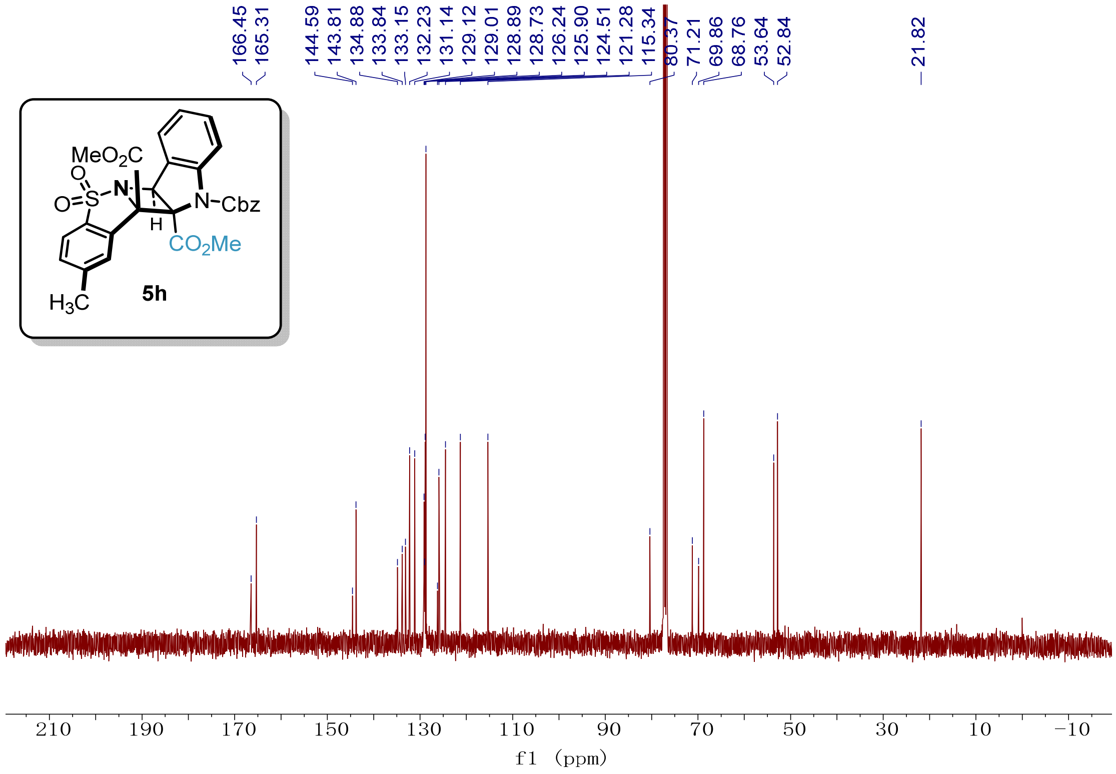

Supplementary Fig. 101. ^{13}C NMR of compound **5h (101 MHz, CDCl₃)**

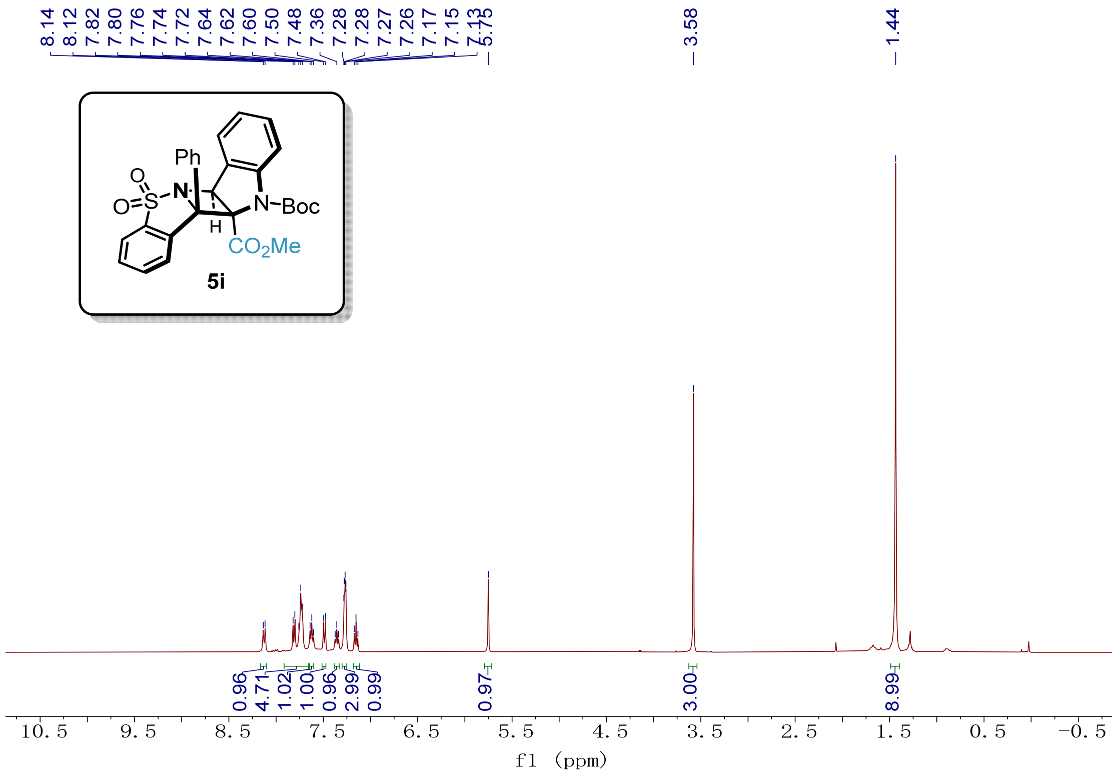

Supplementary Fig. 102. ^{1}H NMR of compound **5i (400 MHz, CDCl$_3$)**

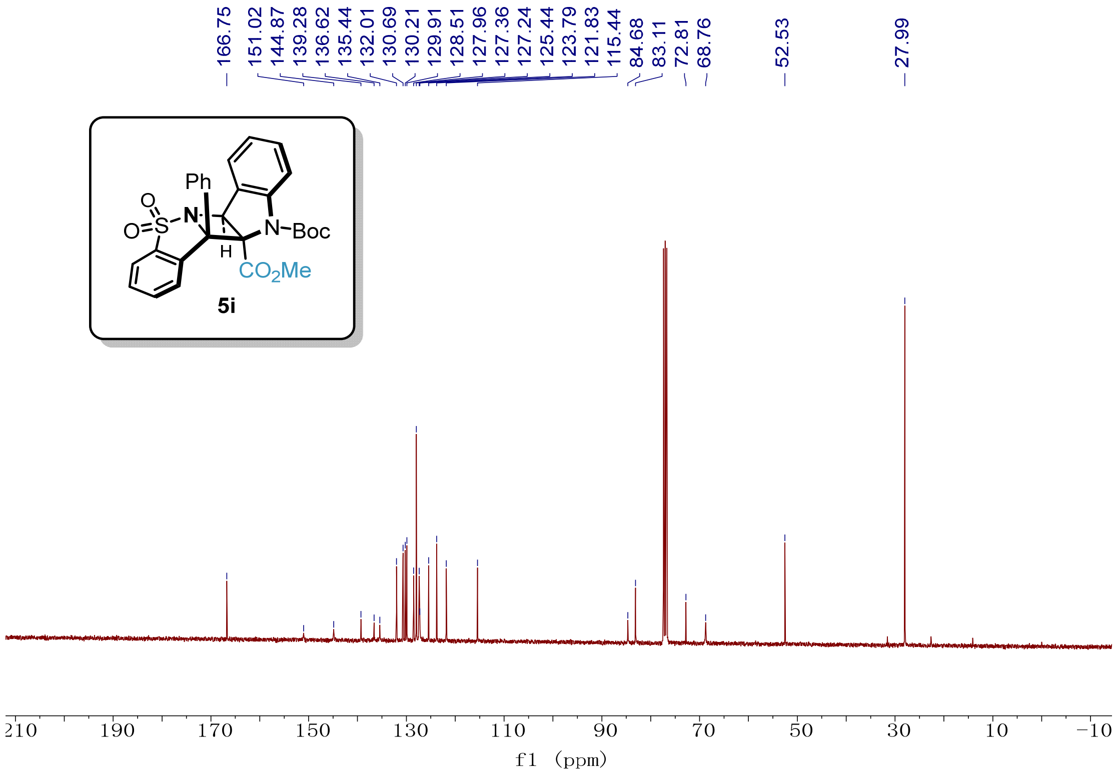

Supplementary Fig. 103. ^{13}C NMR of compound **5i (101 MHz, CDCl$_3$)**

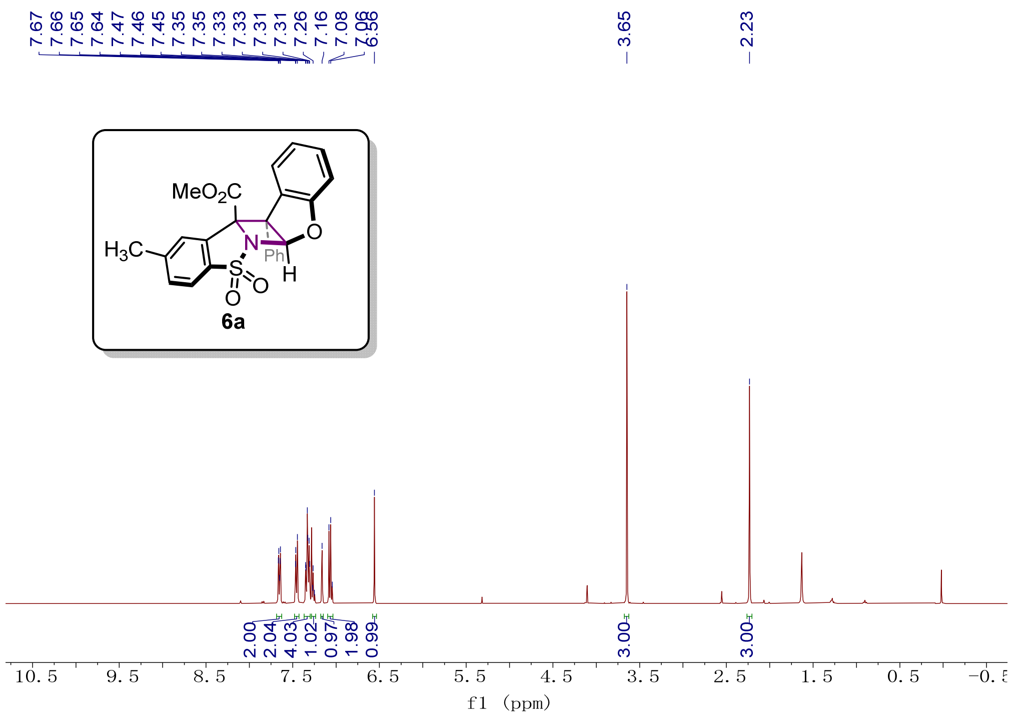

Supplementary Fig. 104. ^{1}H NMR of compound **6a (400 MHz, CDCl$_3$)**

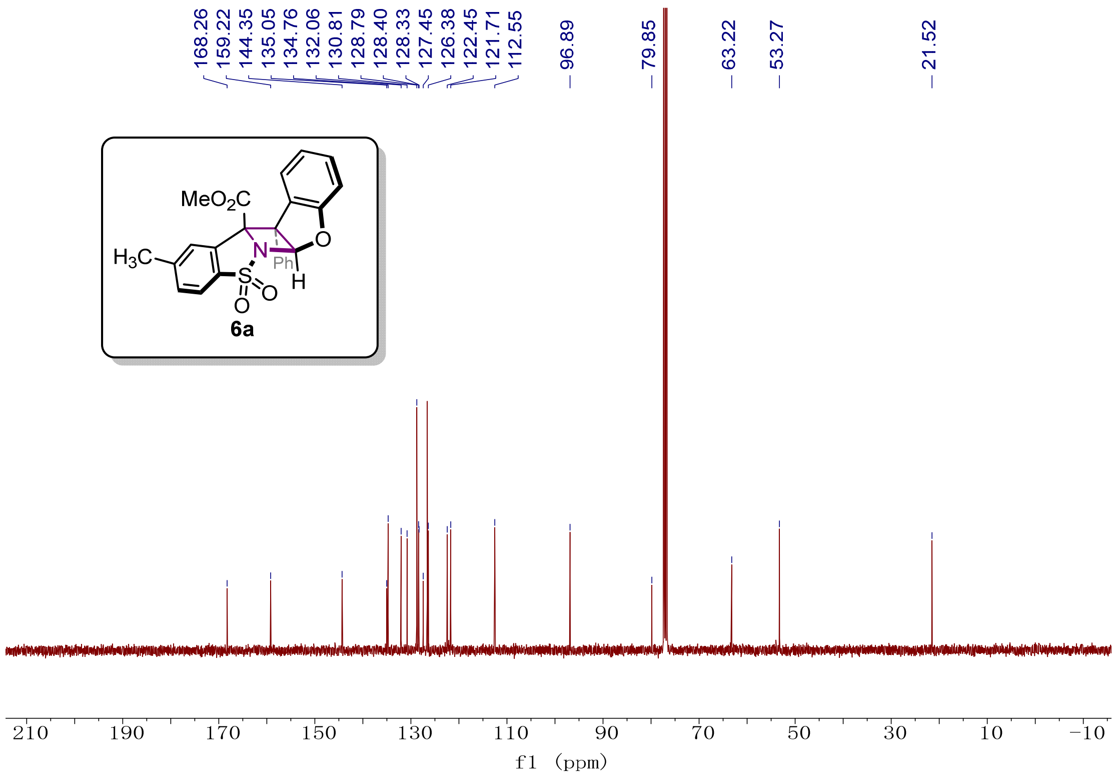

Supplementary Fig. 105. ^{13}C NMR of compound **6a (101 MHz, CDCl$_3$)**

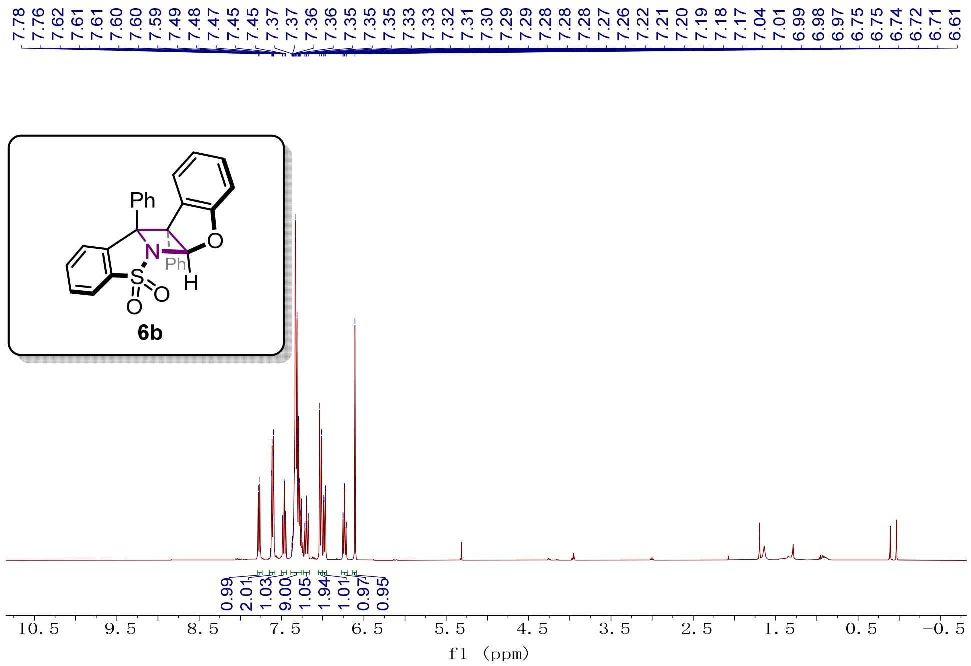

Supplementary Fig. 106. ^{1}H NMR of compound **6b (400 MHz, $CDCl_3$)**

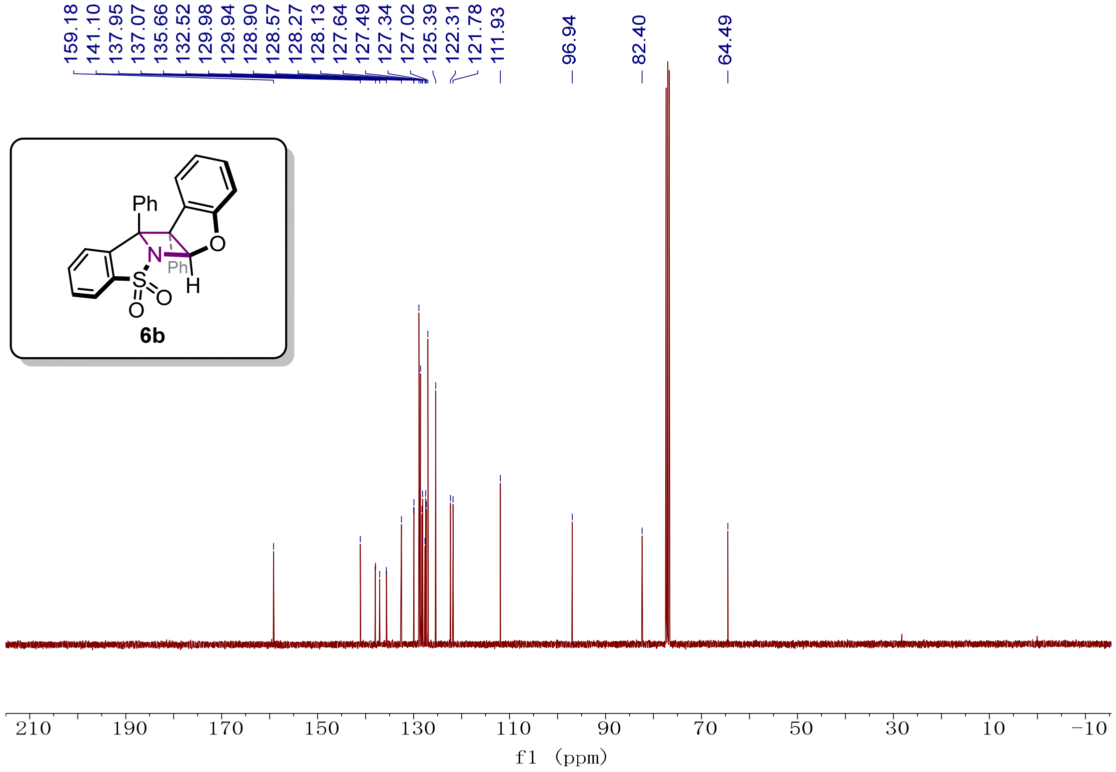

Supplementary Fig. 107. ^{13}C NMR of compound **6b (101 MHz, $CDCl_3$)**

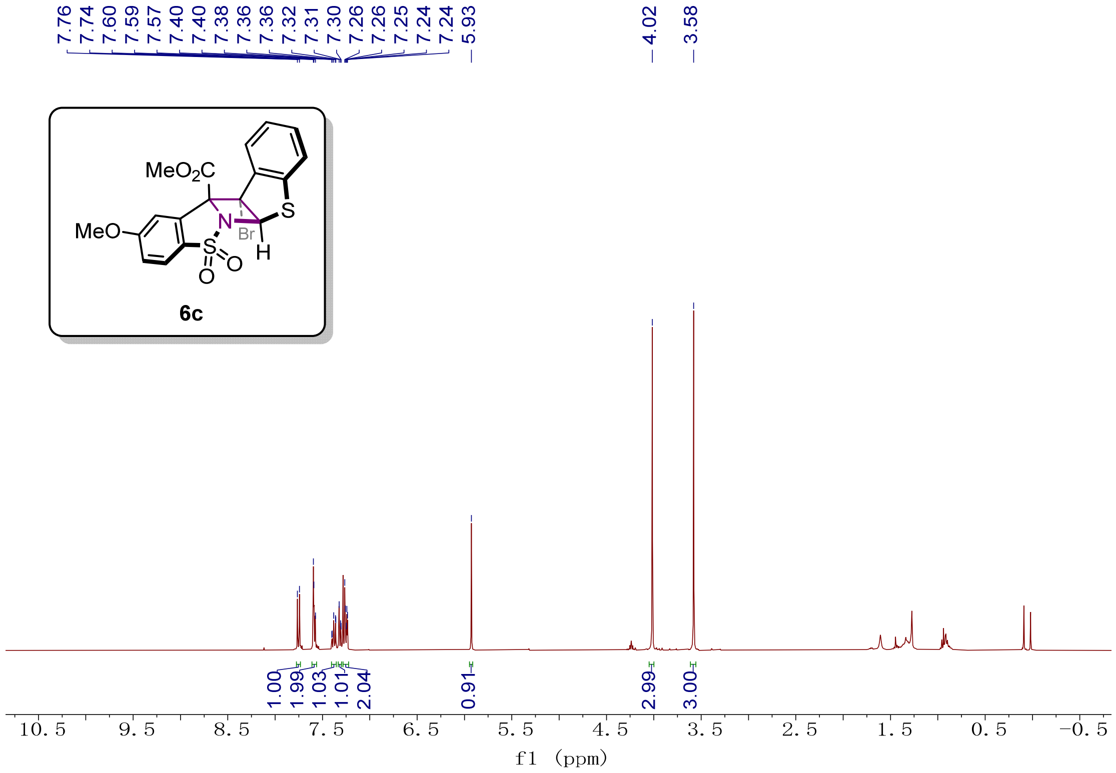

Supplementary Fig. 108. ^{1}H NMR of compound **6c (400 MHz, CDCl$_3$)**

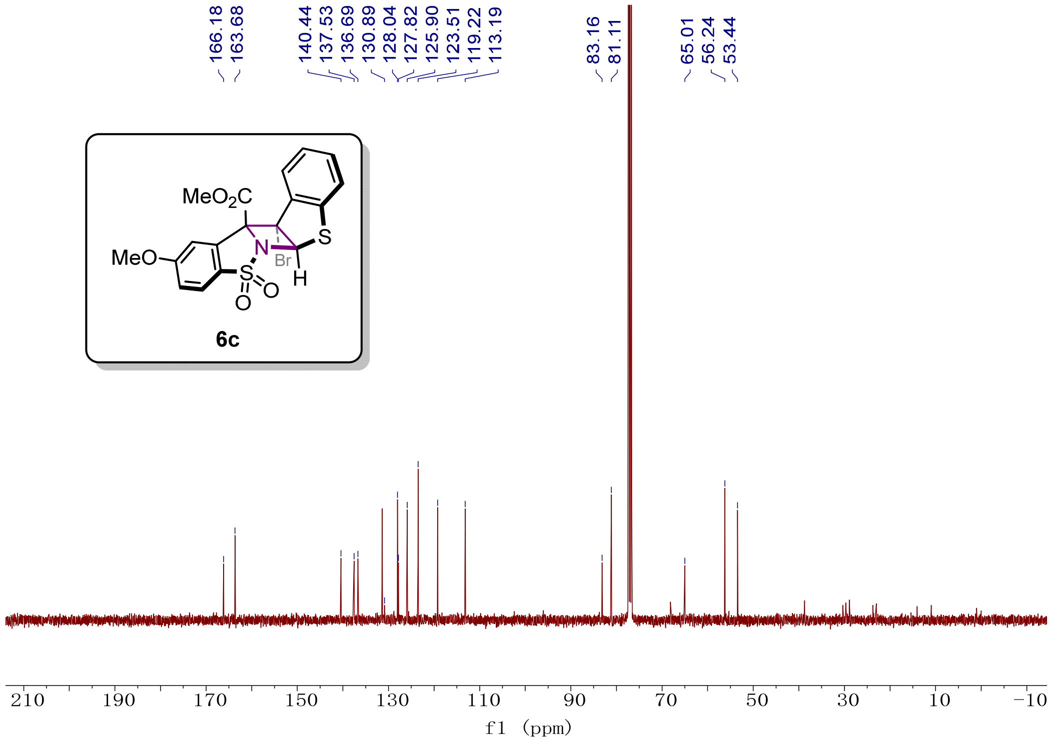

Supplementary Fig. 109. ^{13}C NMR of compound **6c (101 MHz, CDCl$_3$)**

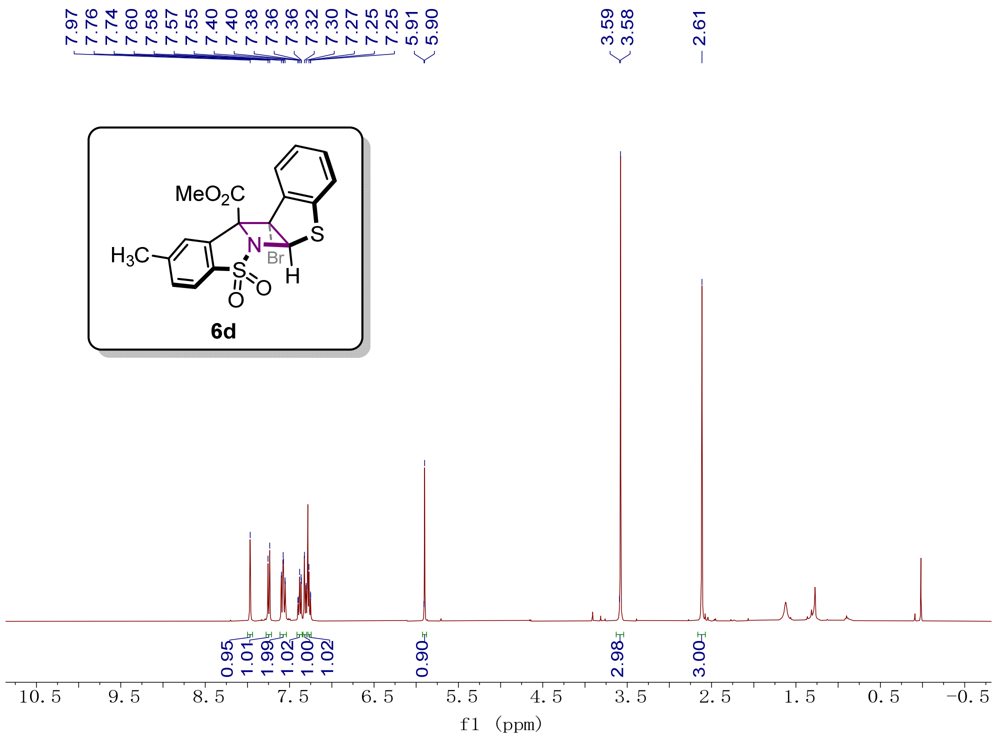

Supplementary Fig. 110. ^{1}H NMR of compound **6d (400 MHz, $CDCl_3$)**

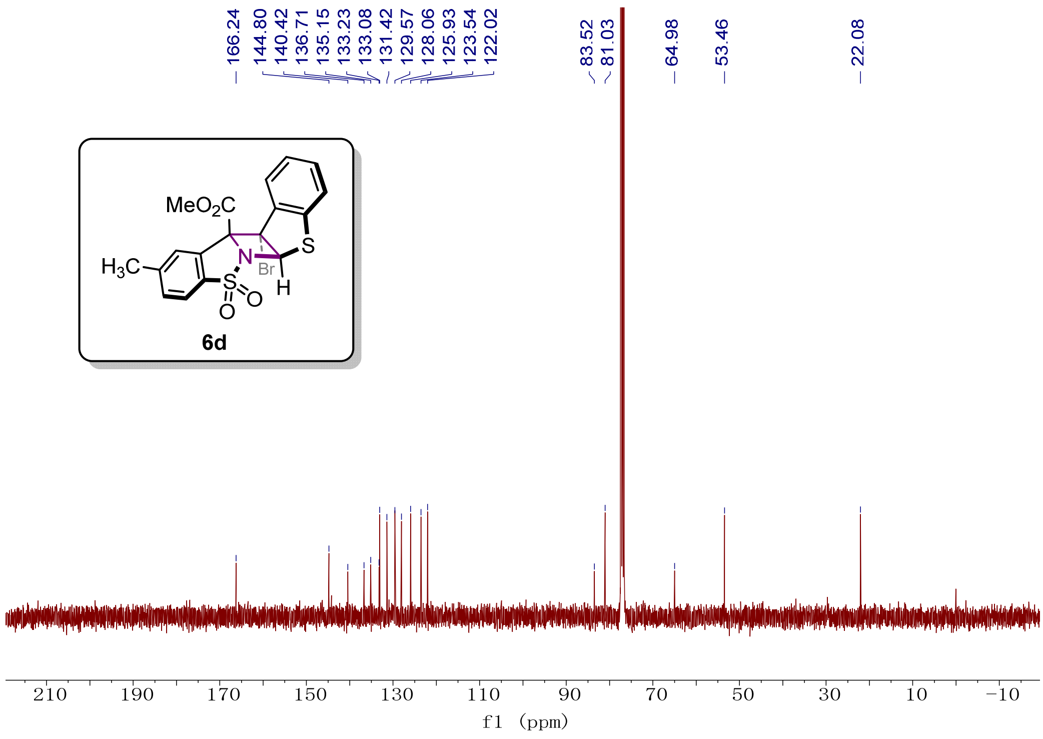

Supplementary Fig. 111. ^{13}C NMR of compound **6d (101 MHz, $CDCl_3$)**

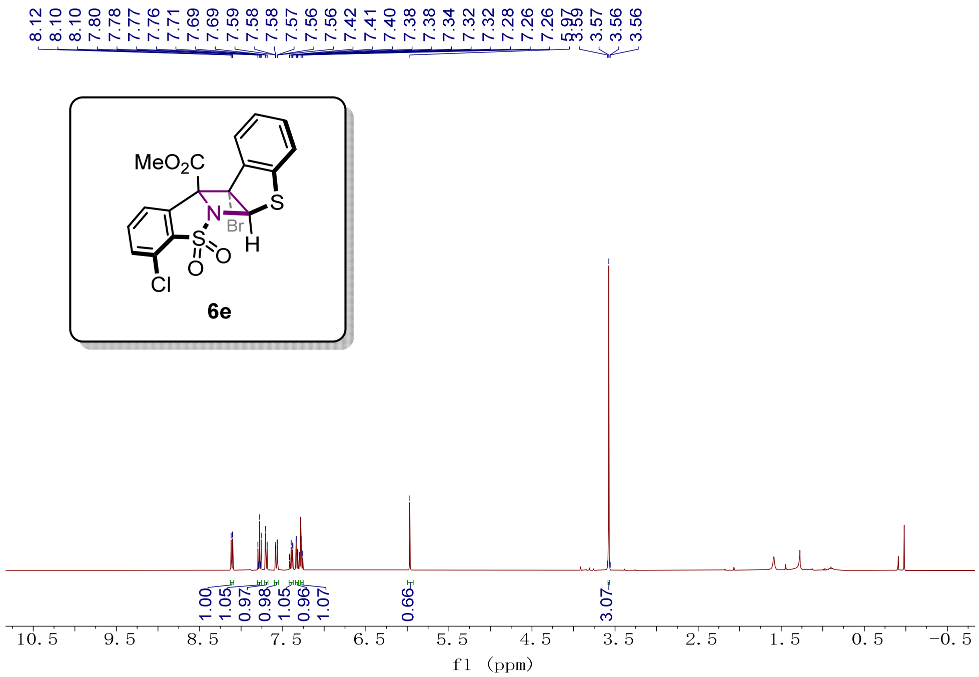

Supplementary Fig. 112. ^{1}H NMR of compound **6e (400 MHz, CDCl$_3$)**

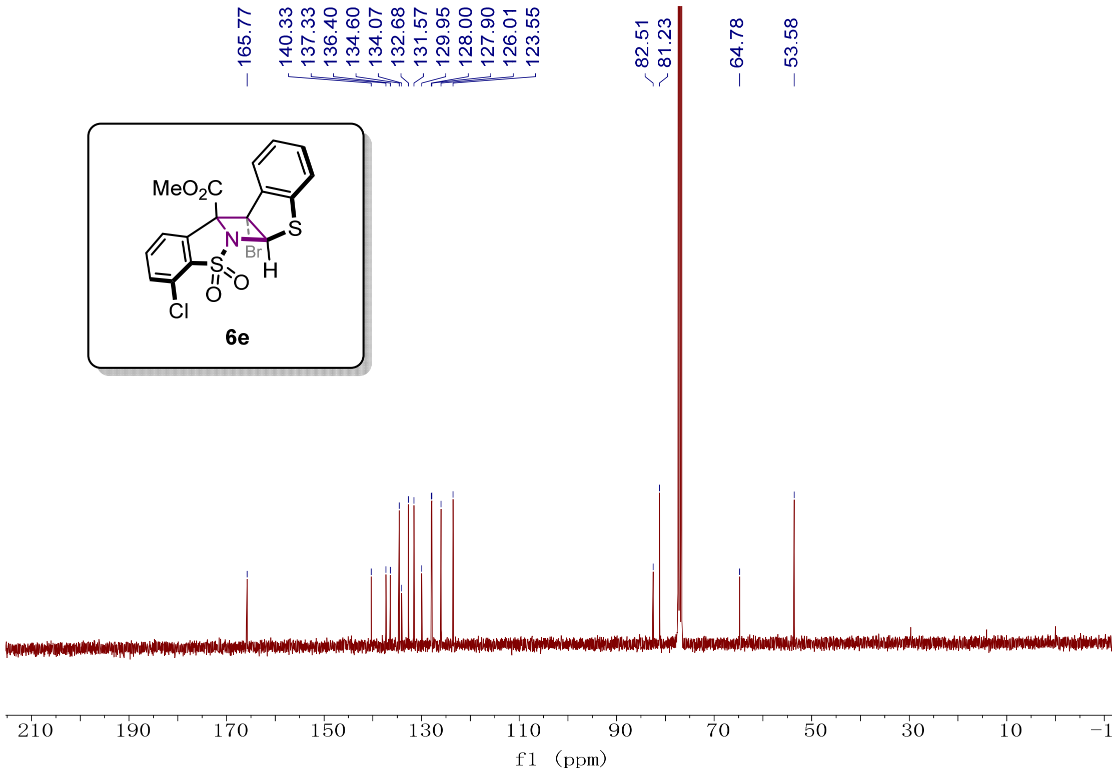

Supplementary Fig. 113. ^{13}C NMR of compound **6e (101 MHz, CDCl$_3$)**

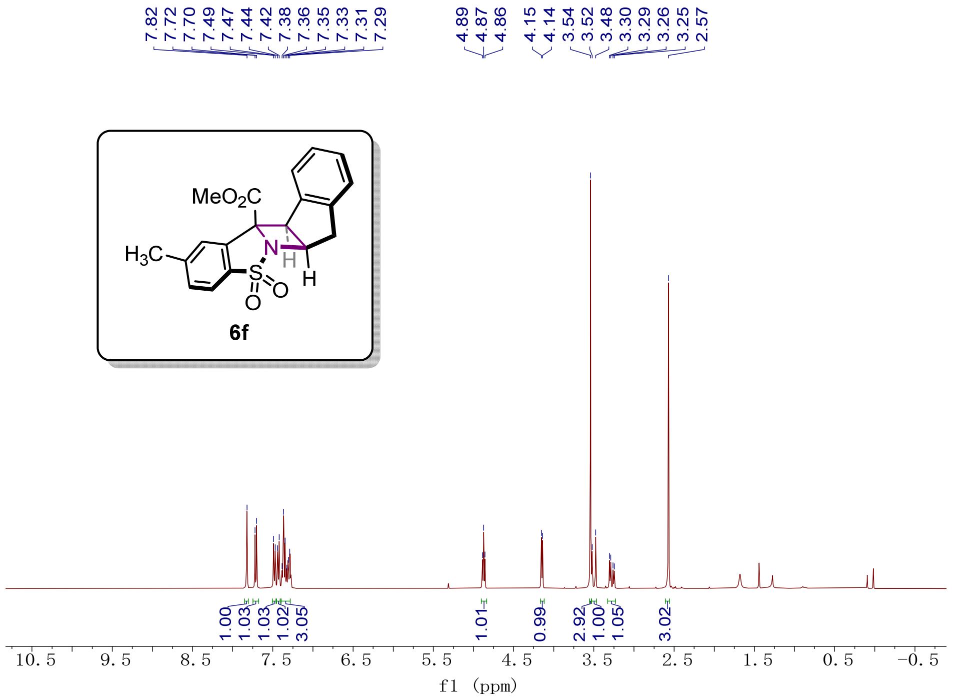

Supplementary Fig. 114. ^{1}H NMR of compound **6f (400 MHz, CDCl$_3$)**

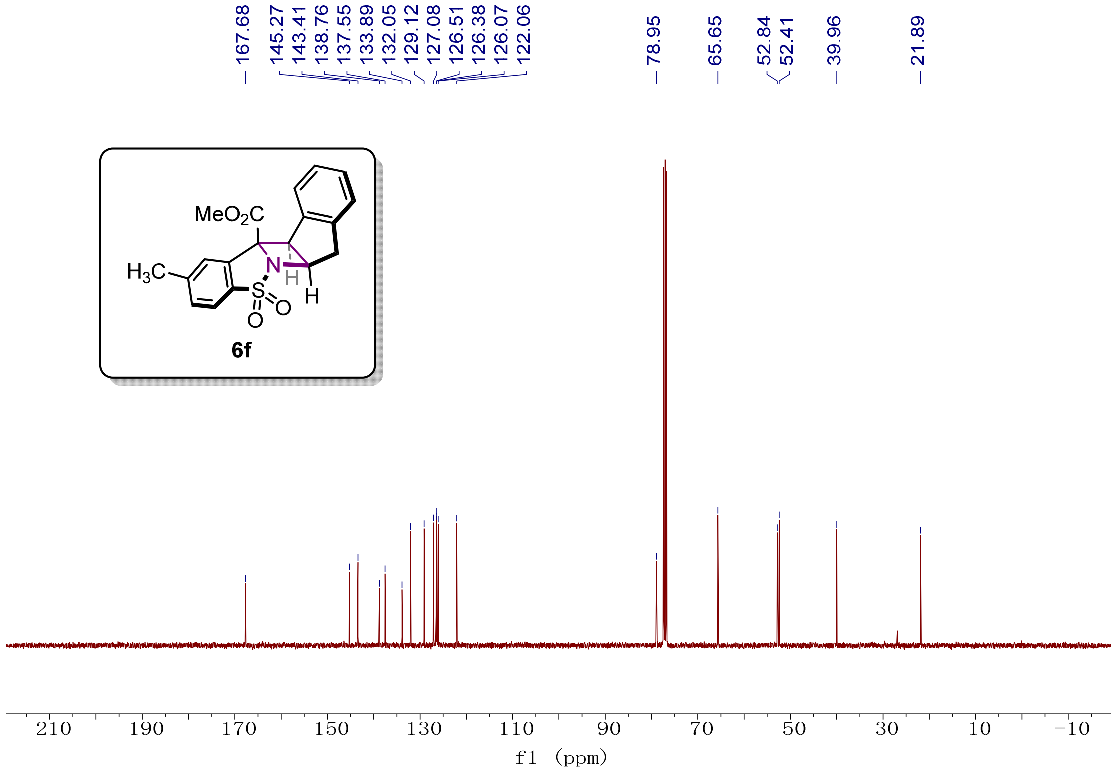

Supplementary Fig. 115. ^{13}C NMR of compound **6f (101 MHz, CDCl$_3$)**

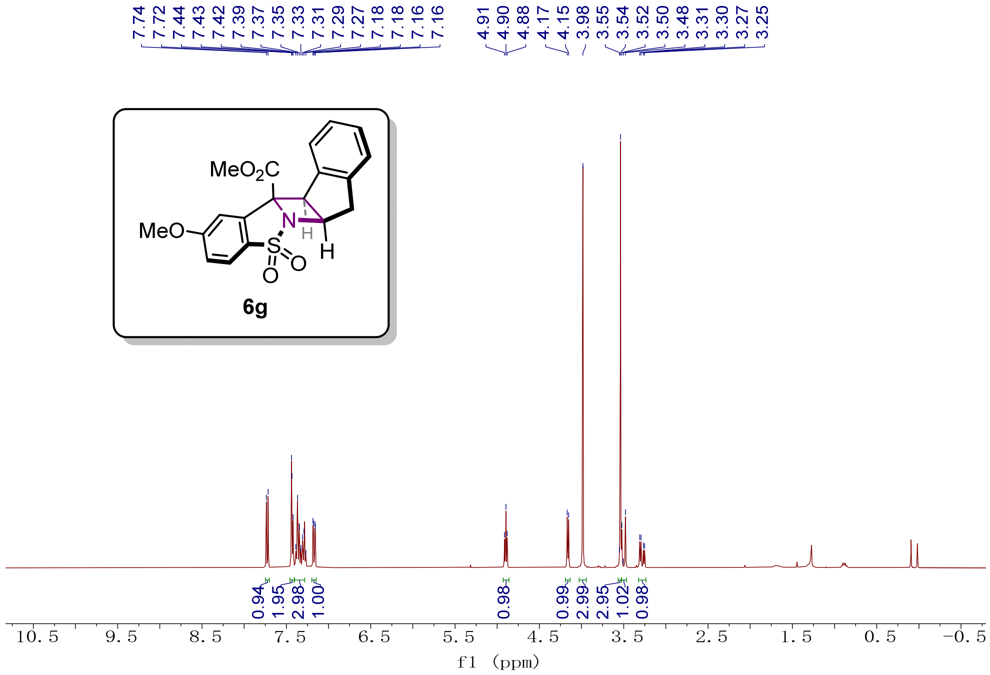

Supplementary Fig. 116. ^{1}H NMR of compound **6g (400 MHz, CDCl$_3$)**

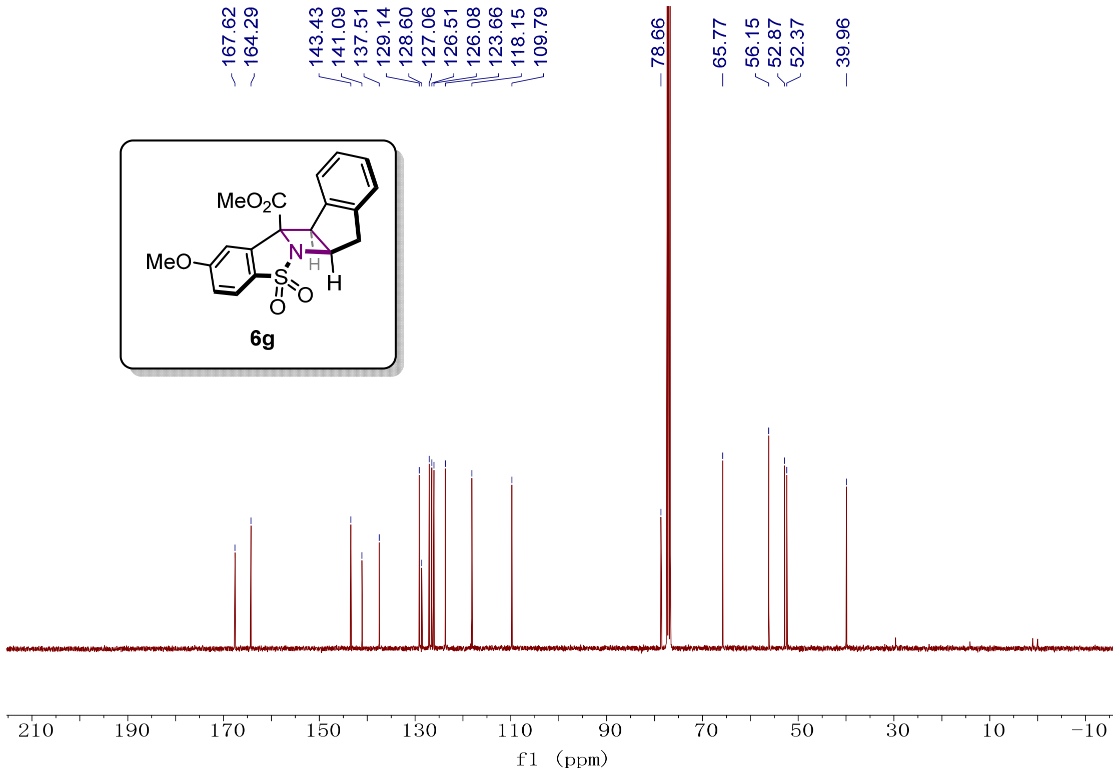

Supplementary Fig. 117. ^{13}C NMR of compound **6g (101 MHz, CDCl$_3$)**
