# Supplementary material for: Accessing ladder-shape azetidine-fused indoline pentacycles through intermolecular regiodivergent aza-Paternò–Büchi reactions
Source: Nat Commun. 2024 Feb 16;15:1431. doi: 10.1038/s41467-024-45687-0 (PMC10873392; doi:10.1038/s41467-024-45687-0)
Supplement: Supplementary file 3 — Description of Additional Supplementary Files [file 41467_2024_45687_MOESM3_ESM.pdf]

File Name: Supplementary Data 1

Description: Cartesian coordinates of optimized structures
